# Protein aggregates encode epigenetic memory of stressful encounters in individual *Escherichia coli* cells

**DOI:** 10.1371/journal.pbio.2003853

**Published:** 2018-08-28

**Authors:** Sander K. Govers, Julien Mortier, Antoine Adam, Abram Aertsen

**Affiliations:** 1 KU Leuven, Department of Microbial and Molecular Systems, Leuven, Belgium; 2 KU Leuven, Department of Computer Science, Leuven, Belgium; Massachusetts Institute of Technology, United States of America

## Abstract

Protein misfolding and aggregation are typically perceived as inevitable and detrimental processes tied to a stress- or age-associated decline in cellular proteostasis. A careful reassessment of this paradigm in the *E*. *coli* model bacterium revealed that the emergence of intracellular protein aggregates (PAs) was not related to cellular aging but closely linked to sublethal proteotoxic stresses such as exposure to heat, peroxide, and the antibiotic streptomycin. After removal of the proteotoxic stress and resumption of cellular proliferation, the polarly deposited PA was subjected to limited disaggregation and therefore became asymmetrically inherited for a large number of generations. Many generations after the original PA-inducing stress, the cells inheriting this ancestral PA displayed a significantly increased heat resistance compared to their isogenic, PA-free siblings. This PA-mediated inheritance of heat resistance could be reproduced with a conditionally expressed, intracellular PA consisting of an inert, aggregation-prone mutant protein, validating the role of PAs in increasing resistance and indicating that the resistance-conferring mechanism does not depend on the origin of the PA. Moreover, PAs were found to confer robustness to other proteotoxic stresses, as imposed by reactive oxygen species or streptomycin exposure, suggesting a broad protective effect. Our findings therefore reveal the potential of intracellular PAs to serve as long-term epigenetically inheritable and functional memory elements, physically referring to a previous cellular insult that occurred many generations ago and meanwhile improving robustness to a subsequent proteotoxic stress. The latter is presumably accomplished through the PA-mediated asymmetric inheritance of protein quality control components leading to their specific enrichment in PA-bearing cells.

## Author summary

Since accurate protein folding is crucial for cellular viability, misfolded and aggregated proteins have typically been thought of as detrimental structures with potentially harmful physiological effects. In this report, we show that this general paradigm does not appear to hold in the model bacterium *Escherichia coli*. We find that the emergence of protein aggregates is mainly linked to sublethal, proteotoxic exposures (e.g., heat stress) and that asymmetric partitioning of these aggregates among daughter cells may have benefits beyond mere waste disposal. In fact, cells that inherited an ancestral protein aggregate (formed during stress exposure many generations before) were better able to cope with subsequent exposure to proteotoxic stress. Our observations therefore reveal the existence of stress-induced, long-term, and protein aggregate–mediated “memory” in prokaryotes and highlight the potential role of protein aggregation in aiding cellular survival and adaptation in fluctuating environments.

## Introduction

Proper protein folding and maintenance of proteome integrity are essential for cell function and viability [1,2]. Nevertheless, the generation of nonnative protein conformations is inevitable to some extent because of the inherent stochastic nature of protein folding [3,4] and is often even aggravated by genetic (e.g., mutations [5]), physiological (e.g., cellular aging [6]), and environmental (e.g., heat [7] or antibiotics [8]) conditions. Such aberrant protein conformations typically expose hydrophobic residues and regions normally buried within their native structure, which drive nonfunctional intermolecular interactions leading to the formation of larger insoluble structures termed protein aggregates (PAs) [9].

In prokaryotes, the occurrence of PAs is regarded as a strictly adverse phenomenon. In the model bacterium *E*. *coli*, for example, intracellular PAs are perceived as naturally occurring, inevitable, and constantly accruing “garbage bins” [10–12]. This view is based on single cell–level observations of growing *E*. *coli* cells, in which the yellow fluorescent protein (YFP)-fused inclusion body binding protein (IbpA-YFP) was employed as a reporter for the presence of aggregated proteins [10,13]. Such PAs were observed to appear randomly during growth and to segregate asymmetrically upon cell division due to their nucleoid-enforced polar localization [10,14–16]. Moreover, PA accumulation in cells with older cell poles was shown to slow down their growth in a dosage-dependent manner, thereby giving rise to the previously observed pattern of aging and rejuvenation in growing microcolonies [10,17]. It was even theorized that this asymmetric damage segregation strategy was superior to damage repair strategies in most environmental conditions and could thus have evolved as an optimal damage riddance strategy [18–21].

This negative perception contrasts with emerging findings in eukaryotes that illustrate the potential functionality of stress-induced misfolded and aggregated proteins. The proteome-wide characterization of aggregation tendencies during thermal stress in the yeast *Saccharomyces cerevisiae* identified heat-induced aggregation and stress-granule formation as specific, reversible, and promoting cellular adaptation and survival [22,23]. Similarly, nutrient depletion of yeast cells leads to the formation of metabolite-specific, reversible protein assemblies that have been proposed to function as storage depots but at the same time might also enhance metabolic efficiency during nutrient stress [24–26]. Detailed characterization and quantification of the *Caenorhabditis elegans* proteome along its lifespan showed extensive proteome remodeling and suggested the sequestration of proteins in insoluble aggregates to be a protective strategy directed toward maintaining proteome integrity during aging [27]. In fact, the term quinary structures has been coined to describe such functional (stress-induced) large protein assemblies, as they lack the fixed stoichiometry specific to quaternary assemblies [28,29]. However, direct phenotypic evidence of their beneficial impact has remained limited. In addition, it remains unclear to what extent the formation of such structures affects and shapes future behavior, after alleviation of the stress conditions.

Given these emerging examples of functional protein aggregation in eukaryotes, we set forward to revisit the occurrence and potential impact of (stress-induced) protein aggregation in prokaryotes using *E*. *coli* as a model system. Using validated fluorescent PA reporters, we could demonstrate that intracellular PAs only emerge as a response to sublethal proteotoxic stresses, after which these structures become asymmetrically segregated and gradually disaggregated. Remarkably, rather than being fitness compromised, we found that cells asymmetrically inheriting an ancestral PA became significantly more resistant to a subsequent stress compared to their isogenic, PA-free siblings. Our results therefore reveal that PAs can serve as epigenetically inheritable memory elements that enable long-term cross-generational reminiscence of previous cellular insults.

## Results

### Previously reported PA occurrence pattern was an artefact of label-induced clustering

**Fig 1:**
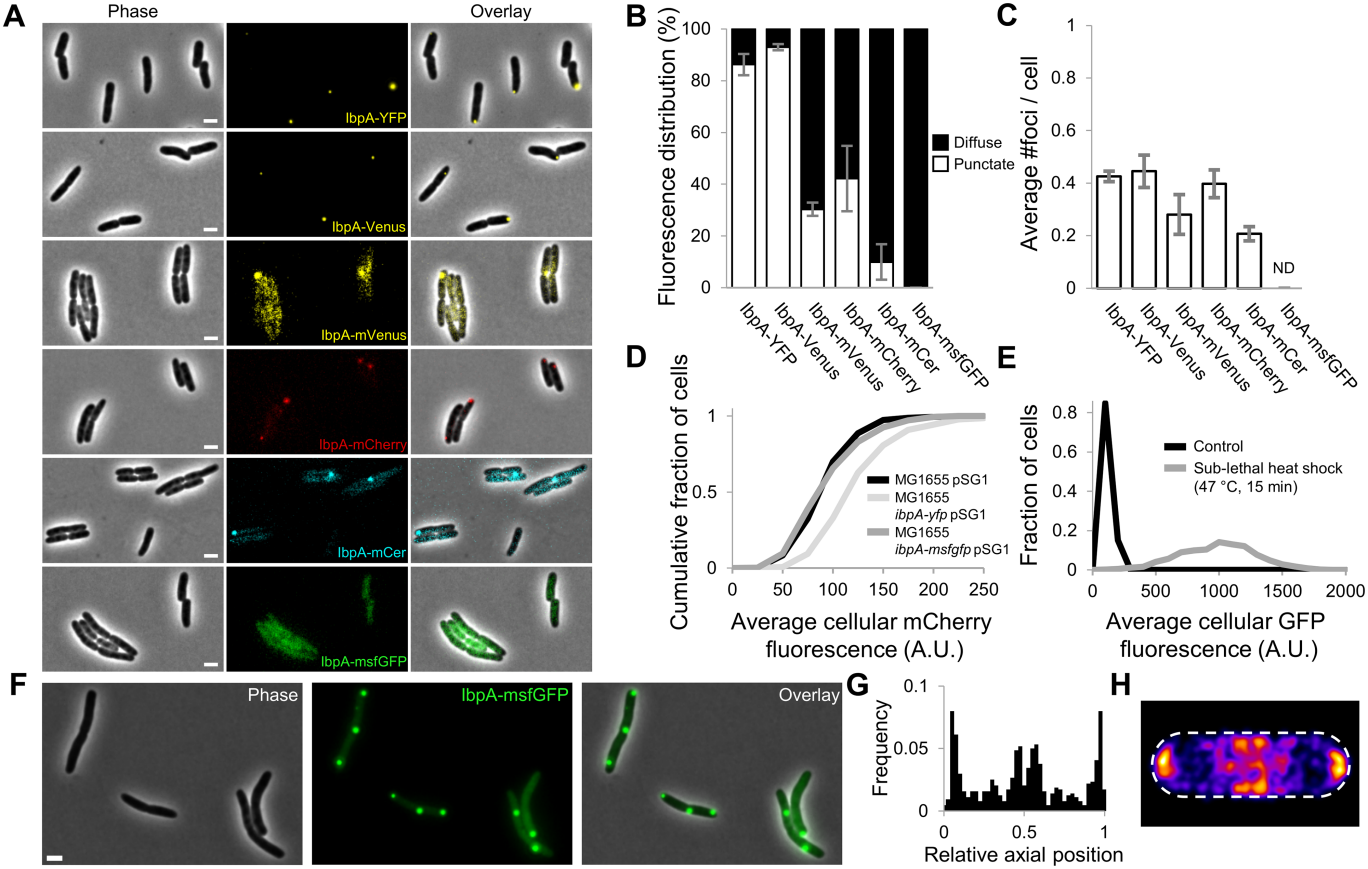
Label-induced mislocalization of IbpA in exponential phase *E*. *coli* cells. (A) Representative phase contrast, epifluorescence (reporting IbpA expression/production and localization), and superimposed images of *E*. *coli* MG1655 cells containing the indicated IbpA fluorescent fusion proteins. Scale bars correspond to 2 μm. (B) Measured distribution of punctate and diffuse fluorescence intensity for the indicated fusion proteins. The fluorescence intensity distribution of at least 25 individual cells was determined per independent experiment. (C) The average number of observed foci per cell for the indicated fusion proteins. Per independent experiment, at least 100 cells were examined to determine the average number of cellular foci. ND = no foci could be detected. For both (B) and (C), the means of 3 independent experiments are shown, with error bars representing the standard deviation between experiments. (D) Cumulative distribution of average cellular mCherry fluorescence of MG1655 pSG1, MG1655 *ibpA-yfp* pSG1, and MG1655 *ibpA-msfgfp* pSG1 cells, derived from 3 independent experiments (*n* ≥ 80 cells per independent experiment). pSG1 is a low-copy plasmid containing a transcriptional P_*ibpA*_*-mCherry* reporter. A K-S test (*p*-value = 3.97 × 10^−19^) indicated significantly increased P_*ibpA*_ expression in MG1655 *ibpA-yfp* cells, whereas this was not the case for MG1655 *ibpA-msfgfp* cells (K-S test; *p*-value = 0.15). (E) Histograms showing the distribution of average cellular GFP fluorescence of control and heat-treated (47 °C, 15 min) MG1655 *ibpA-msfgfp* cells, derived from 3 independent experiments (*n* ≥ 129 per independent experiment). Elevated IbpA levels could be detected in the heat-treated samples (K-S test; *p*-value = 2.23 × 10^−223^). (F) Representative phase contrast, GFP epifluorescence (reporting IbpA expression/production and localization), and superimposed images of MG1655 *ibpA-msfgfp* cells exposed to a sublethal heat shock (47 °C, 15 min). Scale bar corresponds to 2 μm. (G) Binned histograms showing the PA distribution along the relative axial position of MG1655 *ibpA-msfgfp* cells (*n* = 641 cells) exposed to a sublethal heat shock (47 °C, 15 min). (H) Heat map displaying the distribution of PA localization in these cells (*n* = 457 cells). The numerical data underlying this figure can be found in S1 Data. A.U., arbitrary unit; GFP, green fluorescent protein; IbpA, inclusion body binding protein A; K-S, Kolmogorov-Smirnov; mCer, monomeric cerulean; msfGFP, monomeric superfolder GFP; PA, protein aggregate; P_*ibpA*_, *ibpA* promoter; YFP, yellow fluorescent protein.

The *E*. *coli* IbpA protein belongs to the conserved family of ATP-independent small heat shock proteins that readily associate with misfolded and aggregated proteins [30–32]. Previously, *E*. *coli* strains expressing an IbpA-YFP fusion protein have been used to microscopically visualize intracellular PAs, which appeared as punctate and polarly located fluorescent foci inside the cytoplasm of healthy, living cells [10,15,33]. However, given the potential of many commonly used fluorescent proteins to cause label-induced oligomerization and trivial foci formation, this IbpA-YFP reporter has been suggested to yield a biased view on intracellular PA dynamics [34,35]. In order to examine this bias more closely and look for a more reliable PA reporter, we created a set of nearly identical *E*. *coli* MG1655 strains only differing in the fluorescent reporter that was translationally fused to the 3′-end of the native chromosomal *ibpA* gene (Fig 1A, S1A Fig, and S1 Table). The observation that IbpA fusion proteins constructed with monomeric fluorescent protein derivatives (i.e., IbpA-mVenus, IbpA-mCherry, IbpA-monomeric cerulean [mCer], and IbpA-monomeric superfolder green fluorescent protein [msfGFP]) gave rise to lower fractions of punctate cellular fluorescence compared to reporters constructed with nonmonomeric forms (i.e., IbpA-YFP and IbpA-Venus) indeed confirms that the self-aggregating tendency of these fusion proteins can lead to an overestimation of the natural PAs present inside the cell (Fig 1A–1C). In the same vein, inclusion body binding protein B (IbpB)—the other small heat shock protein encoded in the *ibp* operon that associates with PAs through its interaction with IbpA [32]—displayed similar variability in localization when fused to different fluorescent proteins (S1D–S1F Fig), albeit to a lesser degree.

### Validation of IbpA-msfGFP as a reliable reporter for monitoring intracellular PA dynamics

Interestingly, the IbpA-msfGFP reporter (i.e., the fusion equipped with the most monomeric fluorescent label; [35,36]) displayed a strictly diffuse cytosolic fluorescence in exponential phase (Fig 1A–1C) and only exhibited a punctate localization in some cells (12.6%) of a stationary phase population (S1A–S1C Fig). Furthermore, this reporter also displayed wild-type expression levels of the *ibpA* promoter (P_*ibpA*_; which is highly sensitive toward perturbations of protein homeostasis [37–39]), while P_*ibpA*_ activity was markedly increased in the biased MG1655 IbpA-YFP reporter (Fig 1D). Nevertheless, exposure of the MG1655 IbpA-msfGFP reporter to a sublethal heat shock led to significantly increased *ibpA* expression (Fig 1E) and the emergence of multiple foci (mainly localized in the polar and mid-cell regions), confirming its ability to sense and report protein aggregation (Fig 1F–1H and S2A and S2B Fig). Moreover, sublethal treatment of this reporter strain with streptomycin—conditions known to induce mistranslation and subsequent protein misfolding [8,10]—induced a similar response in which emerging IbpA-msfGFP foci colocalized with the streptomycin-induced inclusion bodies (visible in the phase contrast images; S2C–S2E Fig). Inclusion body formation driven by the production of recombinant protein also instigated a similar up-regulation and colocalization phenotype (S2F–S2I Fig), further forwarding IbpA-msfGFP as a reliable reporter for monitoring native intracellular PA dynamics.

### PA formation is limited to sublethal stressful encounters

**Fig 2:**
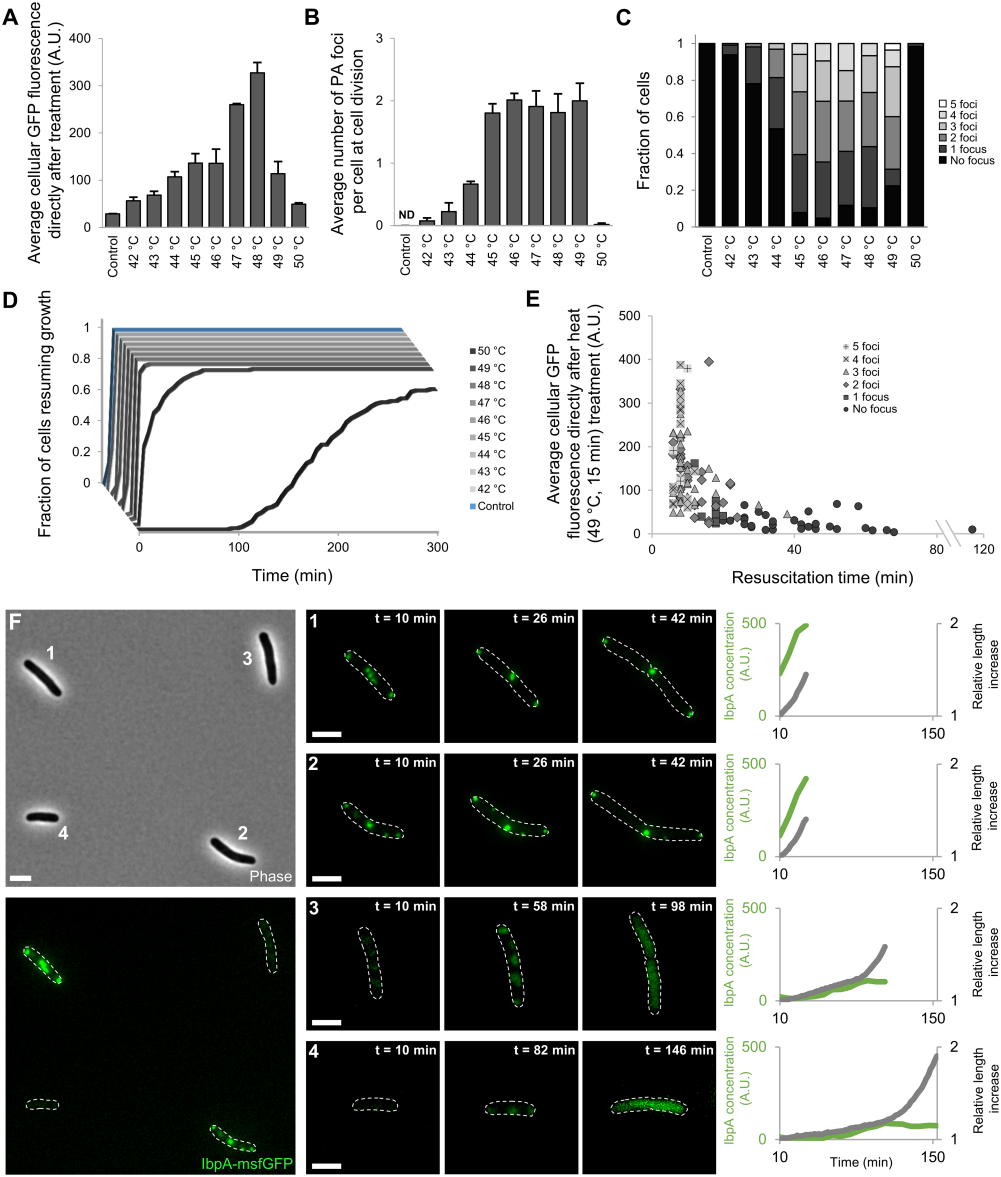
PA formation is limited to sublethal temperatures. (A) Average IbpA-msfGFP concentration in MG1655 *ibpA-msfgfp* populations exposed to the indicated temperatures (15 min), measured immediately after heat treatment. (B) Average number of PA foci at cell division in those same populations. The number of PA foci was scored at cell division to ensure consistent comparison between conditions, given that occasional fusion and/or resolution of PAs before resumption of growth and division could be observed. For both (A) and (B), the means of 3 independent experiments are shown with error bars representing the standard deviation. ND = no foci could be detected. (C) Bar graphs illustrating the distribution of the number of observed PA foci per cell after exposure to the indicated temperatures. (D) Cumulative resuscitation time distributions of MG1655 *ibpA-msfgfp* cells after indicated heat treatments (15 min), derived from 3 independent experiments. The time individual resuscitating cells needed to resume growth was determined and binned to create the cumulative lag-time distributions. For (A-D), the total number of observed cells per independent experiment varies per applied temperature (*n* ≥ 32, *n* ≥ 43, *n* ≥ 45, *n* ≥ 39, *n* ≥ 33, *n* ≥ 33, *n* ≥ 29, *n* ≥ 25, *n* ≥ 40, and *n* ≥ 32 cells for control cells and populations exposed to 42 °C, 43 °C, 44 °C, 45 °C, 46 °C, 47 °C, 48 °C, 49 °C, and 50 °C, respectively). (E) Scatter plot illustrating the negative correlation (Spearman’s ρ = −0.7118, *p*-value = 2.98 × 10^−20^) between the initial IbpA concentration and resuscitation times of individual cells (*n* = 123) exposed to 49 °C (15 min). (F) Representative image illustrating the apparent bifurcation phenotype observed in MG1655 *ibpA-msfgfp* populations exposed to 49 °C (15 min). Left, representative phase contrast and GFP epifluorescence (reporting IbpA expression/production and localization) image of cells directly after the heat treatment. Middle, representative GFP epifluorescence images at indicated times after exposure to 49 °C (15 min). For each row, the brightness of the images was adjusted to allow for better visualization of the intracellular pattern. Cell outlines are shown in white. Scale bars correspond to 2 μm. Right, graphs displaying the evolution of relative cell length (gray lines) and total cellular IbpA concentration (green lines) of the cells depicted in the left and middle images. Cellular behavior was tracked up until the first division event. The numerical data underlying this figure can be found in S1 Data. A.U., arbitrary unit; GFP, green fluorescent protein; IbpA, inclusion body binding protein A; msfGFP, monomeric superfolder GFP; PA, protein aggregate.

After validating IbpA-msfGFP as a truthful marker for the presence of natively occurring PAs, we set forward to further probe their occurrence and physiological impact. To this end, we exposed exponentially growing MG1655 *ibpA-msfgfp* cells to a range of sublethal heat shocks (temperatures higher than 50 °C led to the inactivation of a substantial fraction of cells and were subsequently not considered in this analysis) and monitored their response and subsequent outgrowth by time-lapse fluorescence microscopy (TLFM). While both IbpA up-regulation and IbpA-msfGFP foci formation originally increased with the severity of the heat shock, this coordinated behavior displayed remarkable alterations at higher sublethal temperatures. The number of visible PA foci per cell leveled off at temperatures higher than 45 °C, and IbpA expression reached a maximum at 47–48 °C (Fig 2A–2C). Given the observed foci account for most of the cellular fluorescence, this indicates that in between this temperature range (45–48 °C), larger but not more PAs were formed. After exposures to higher temperatures (49–50 °C), both the expression level and the number of PAs declined, and a significant delay in cellular growth resumption could also be observed (Fig 2D).

Within populations exposed to the same temperature, a large variability in the extent of protein aggregation (both in the number of visible PAs as in the amount of IbpA-msfGFP fluorescence) could be observed with cells containing 0 to 5 distinct IbpA-msfGFP foci at cell division after exposure to the sublethal heat treatment (Fig 2C). Populations exposed to higher sublethal temperatures (48–49 °C) displayed a remarkable bifurcation phenotype (Fig 2E and 2F). Although a significant fraction of cells behaved similarly as recorded for lower sublethal temperatures, displaying clear IbpA up-regulation (which persisted during initial growth resumption) and foci formation, others exhibited neither of these characteristics (Fig 2F). This fraction of cells did display belated IbpA up-regulation but not to the same extent as their PA-forming counterparts. Moreover, an initial “patchy” IbpA-msfGFP localization pattern could be observed in these cells, which appeared to be resolved concomitantly with IbpA up-regulation as cells resumed growth and division (Fig 2F). Interestingly, whereas the PA-forming fraction of cells readily resumed growth, this other non-PA-forming fraction displayed significantly longer resuscitation times (Fig 2E and 2F). Whereas only a limited number of cells (10.5%) displayed this behavior after exposure to 48 °C, this fraction, as well as their average resuscitation time, became significantly larger (22.5%) in populations exposed to 49 °C (Fig 2D and 2E). At higher temperatures (50 °C), this bifurcation disappeared, and the behavior of (surviving) cells shifted completely toward attenuated IbpA up-regulation without PA formation combined with longer resuscitation times (Fig 2D). Although the mechanisms underlying the observed bifurcation and phenotypic shift remain elusive, our observations highlight an apparent plasticity in the formation of PAs. This suggests that PA formation might be more than an inevitable artefact of exposures to elevated temperatures, in which case PAs would be observed in surviving cells after exposure to all temperatures above a given threshold.

### Asymmetric inheritance of PAs formed after sublethal proteotoxic encounters

**Fig 3:**
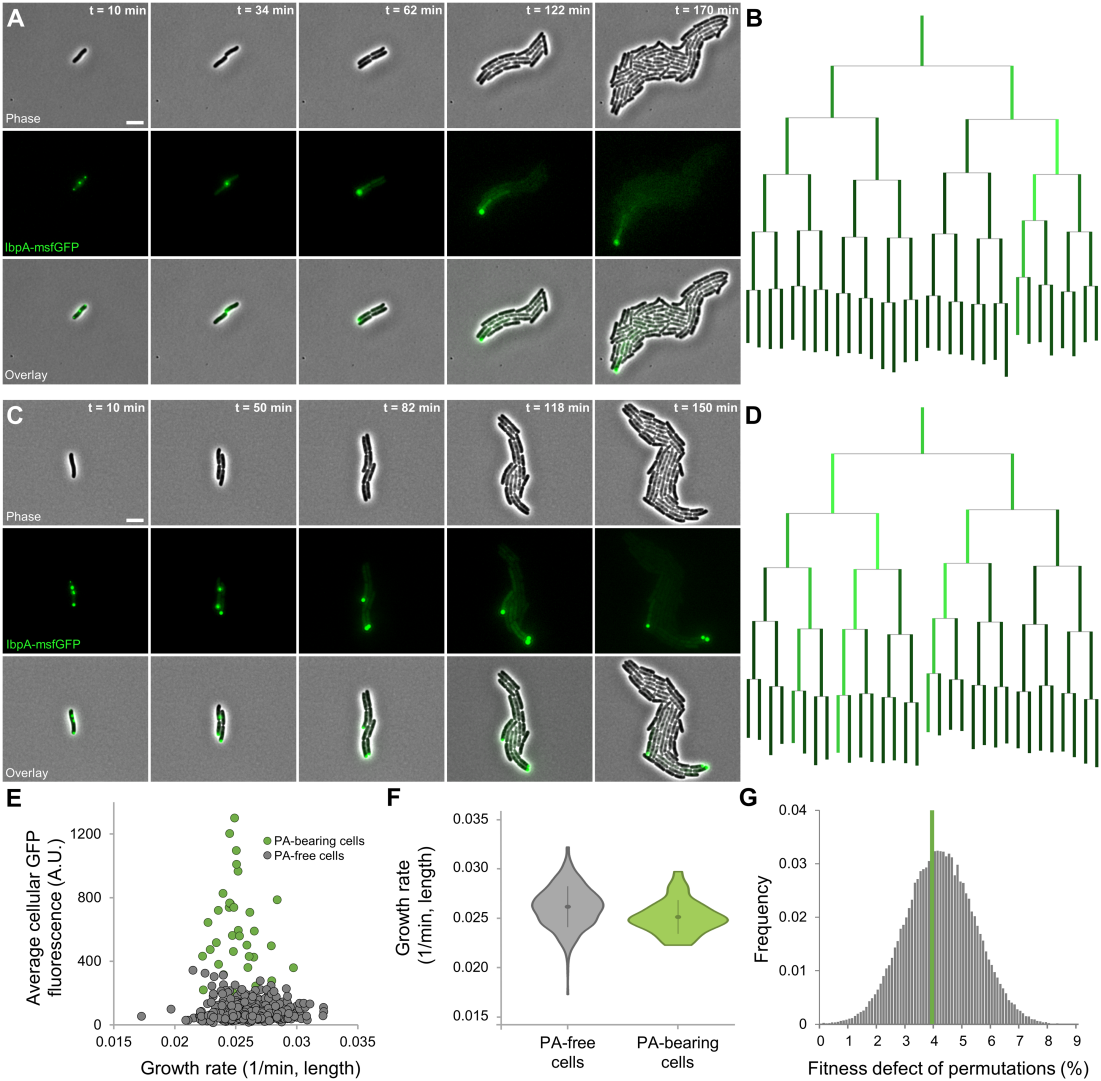
Asymmetric segregation of native PAs raised by exposure to sublethal heat shock. (A and C) Representative phase contrast, GFP epifluorescence (reporting IbpA concentration and localization), and superimposed images of a TLFM image sequence of two MG1655 *ibpA-msfgfp* cells growing into microcolonies after exposure to a sublethal heat shock (47 °C, 15 min). Scale bars correspond to 5 μm. (B and D) Corresponding lineage trees in which the length of lines connecting cells to their progeny is proportional to the relative growth rate of that cell, and the color of these lines is proportional to the relative average GFP fluorescence of that cell. At each division except the first—in which the identity of the poles is not known, and cells are positioned randomly—the cell inheriting the old pole is placed on the left side of the division pair. (E) Correlation between average cellular GFP fluorescence and growth rate of individual fourth- and fifth-generation MG1655 *ibpA-msfgfp* cells (*n*_microlonies_ = 10, *n*_cells_ = 478) after exposure to a sublethal heat shock (47 °C, 15 min). Color indicates whether the respective cell was PA-bearing or PA-free. No strong correlation could be observed between both variables (Pearson’s r = 0.1269, *p*-value = 8.31 × 10^−2^), not even within PA-bearing cells (Pearson’s r = 0.0927, *p*-value = 3.43 × 10^−1^, *n* = 34). (F) Violin plots comparing the distribution of growth rates of the same PA-free and PA-bearing cells (*n* = 444 and *n* = 34, respectively). Although a significant difference could be detected between the average growth rate of both classes (3.97%, Student *t* test, *p*-value = 1.67 × 10^−3^), a permutation test revealed that this could be attributed to the underlying age structure of PA-bearing cells (*p*-value = 0.56). (G) Distribution of fitness defects observed for permutations (100,000) comparing randomly selected PA-free cells (of similar age as the observed PA-bearing cells) to all other PA-free cells. Green line indicates the empirically observed fitness defect for PA-bearing cells (3.97%). The numerical data underlying this figure can be found in S1 Data. A.U., arbitrary unit; GFP, green fluorescent protein; IbpA, inclusion body binding protein A; msfGFP, monomeric superfolder GFP; PA, protein aggregate; TLFM, time-lapse fluorescence microscopy.

To further examine the impact of PAs on cellular physiology, we exposed cells to a sublethal heat treatment leading to maximal PA formation while minimally compromising physiology and subsequent proliferation (47 °C, 15 min). We quantitatively characterized the growth of these cells over multiple generations in detail with TLFM. As the cells resumed growth, PAs typically remained intact, became localized in the cell poles, and segregated asymmetrically while cells grew out into microcolonies (Fig 3A–3D, S1 and S2 Movies). Although the existence of so-called cellular aging could be detected in these microcolonies (9.02% decrease in cellular growth rate of cells inheriting the oldest cell pole compared to the rest of the population; S2J Fig), stochastic partitioning of PAs during the first generation post heat treatment led to only 6 out of a total of 40 observed oldest cells containing a PA, suggesting both phenomena might not be associated with each other. Subsequent examination of aging in PA-free cells indeed confirmed this phenomenon to occur independently of protein aggregation (9.36% decrease in cellular growth rate; S2J Fig). Moreover, a permutation test revealed that the limited average fitness defect of PA-bearing cells does not differ from that of PA-free cells with a similar age structure (*p*-value = 0.56; Fig 3E–3G), indicating PAs themselves impart no significant growth defect on their individual host cells. This finding was further strengthened by the lack of correlation between cellular growth rate and average cellular GFP concentration for PA-bearing cells (Fig 3E).

To assure that the phenomenon of asymmetrically inherited PAs indeed represents native behavior and is not a trivial consequence of the fluorescent labeling of IbpA with a monomeric fluorescent protein, we equipped wild-type MG1655 cells (thus containing an unlabeled native copy of *ibpA*) with a vector (pBAD33-*ibpA-msfgfp*) in which expression of the IbpA-msfGFP fusion protein was placed under an arabinose-inducible promoter. These cells were grown in repressing conditions (0.2% glucose) to exponential phase, exposed to a sublethal heat treatment (47 °C, 15 min), and subsequently monitored on arabinose-containing (0.15%) agarose pads by TLFM (S3 Fig). As cells grew under these inducing conditions, a gradual increase in cellular fluorescence could be observed, followed by the appearance of one or multiple distinct foci that remained present during the rest of recording (S3A Fig). While a similar increase in cellular fluorescence could also be observed in control cells not subjected to a sublethal heat treatment, this increase was not accompanied by the manifestation and stable inheritance of distinct fluorescent foci (S3B Fig). Together, these findings indicate that the presence of fluorescently labeled IbpA is not a prerequisite for the formation of asymmetrically segregating PAs (which become apparent upon induction of the IbpA-msfGFP fusion protein), demonstrating that the latter is indeed a natively occurring phenomenon after exposure to a sublethal heat treatment. Remarkably, higher induction levels of the fusion protein led to the irregular formation (i.e., continuously, not only initially when cellular fluorescence increased) of multiple fluorescent foci in both heat-treated and control cells (S3C Fig). This suggests that increased expression of IbpA itself, in line with previous observations describing the emergence of PAs upon overproduction of IbpA [40,41], might contribute to the formation of these structures in proteotoxic stress conditions (as these are known to induce *ibpA* expression).

We noticed that the asymmetric segregation of PAs was accompanied by an IbpA-msfGFP concentration gradient stemming from the PA-bearing cell(s) and progressively diminishing in its closest relatives (Fig 3A–3D). To examine whether this concentration gradient could be the consequence of increased *ibpA* expression in PA-bearing cells, we performed an identical experiment with MG1655 *ibpA-msfgfp* pSG1 cells, which allow the concurrent monitoring of IbpA promoter activity, concentration, and localization (S4A Fig). Although these cells behaved similarly after exposure to a sublethal heat shock in terms of PA formation, localization, and asymmetric segregation (S4B Fig), no increased mCherry signal could be detected in PA-bearing cells (as compared to that of their PA-free counterparts; S4C Fig). This indicates that the observed concentration gradient is likely not the consequence of a transcriptional *ibpA* response to the presence of these PAs but presumably finds it origin in the gradual disaggregation of the existing PA. In agreement, a small fraction of intracellular PAs was even observed to disappear after a certain amount of time, presumably because of their complete disaggregation.

Intriguingly, the above described phenomenon was not only instigated by a sublethal heat treatment. Exposure to other sublethal environmental stressors impacting proteostasis, such as streptomycin or hydrogen peroxide, gave rise to similar PA-associated phenotypic behavior (S5 Fig). As such, the formation and subsequent asymmetric segregation of these structures appears to be a conserved phenomenon throughout a wide range of sublethal environmental conditions affecting cellular proteostasis.

### PAs increase survival probability of individual cells upon encountering a second, more severe heat shock

**Fig 4:**
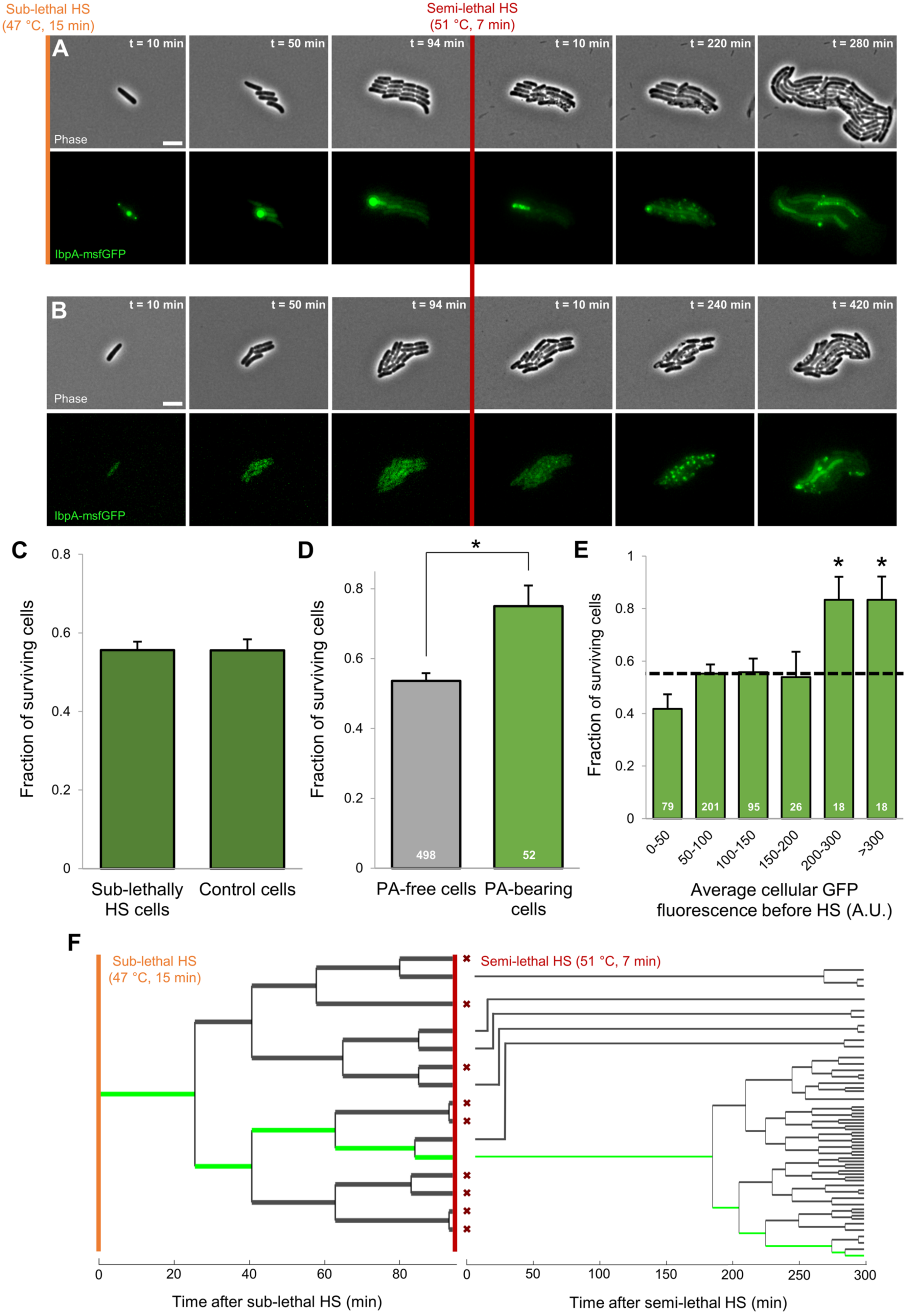
Presence of PAs increases survival probability of individual cells within microcolonies upon semilethal HS. (A-B) Representative phase contrast and GFP epifluorescence (reporting IbpA expression/production and localization) images of a TLFM image sequence of growing MG1655 *ibpA-msfgfp* cells before and after application of an HS (51 °C, 7 min) killing approximately half of the cells. Shown are (A) MG1655 *ibpA-msfgfp* cells exposed to a prior sublethal HS (47 °C, 15 min) and (B) unstressed MG1655 *ibpA-msfgfp* cells. Scale bars correspond to 5 μm. (C) Average survival probability of sublethally heat-shocked (47 °C, 15 min) and unstressed control MG1655 *ibpA-msfgfp* cells (*n*_microlonies_ = 38, *n*_cells_ = 550 and *n*_microlonies_ = 22, *n*_cells_ = 315, respectively) upon exposure to a more lethal HS (51 °C, 7 min). No significant difference could be detected in survival frequency (Student *t* test, *p*-value = 0.98). Survival was defined as the ability of a cell to resume growth and subsequent division within 8 h after treatment. (D) Average survival probability of PA-free and PA-bearing MG1655 *ibpA-msfgfp* cells exposed to a prior sublethal HS (*n* = 498 and *n* = 52 cells, respectively) upon exposure to a second, more lethal HS (51 °C, 7 min). Asterisk indicates a significant difference in survival frequency between both cellular classes (Student *t* test, *p*-value = 1.33 × 10^−3^). (E) For PA-containing microcolonies, the fraction of cells surviving the second HS (51 °C, 7 min) is binned by the average cellular fluorescence before this HS. The dotted line indicates average survival of all cells; asterisks indicate bins that significantly differ (Fisher’s exact test, *p*-values = 1.50 × 10^−2^). Numbers in white indicate the number of cells included in each bin. Error bars for (C)–(E) indicate bootstrapped estimates of the standard error of the mean fraction of surviving cells. (F) Lineage tracing for the microcolony displayed in (A) illustrating growth, division, and survival of cells throughout the course of the entire experiment. Green lines indicate the PA-bearing cells. Red crosses indicate cells that did not survive the semilethal HS. At each division except the first—in which the identity of the poles is not known, and cells are positioned randomly—the cell inheriting the old pole is placed on the bottom side of the division pair. The numerical data underlying this figure can be found in S1 Data. GFP, green fluorescent protein; HS, heat shock; IbpA, inclusion body binding protein A; msfGFP, monomeric superfolder GFP; PA, protein aggregate; TLFM, time-lapse fluorescence microscopy.

Since PAs thus appear as asymmetrically segregating remnants of a previously encountered proteotoxic stress, we wondered to what extent the presence of these structures (and the resulting concentration gradient) could influence survival upon a subsequent stress exposure. To this end, we challenged a total of 38 lineages stemming from sublethally heat-shocked cells (47 °C, 15 min) to a subsequent semilethal heat shock (51 °C, 7 min; Fig 4A), an assay in which we have previously described cellular survival to behave as a stochastic trait free of predispositions [42]. The heterogeneity in PA formation gave rise to microcolonies harboring a variable number of PAs, with an average of 1.41 PA foci per microcolony at the moment of the second heat treatment. Average cellular survival within these microcolonies (i.e., 55.6%) did not significantly differ from that within microcolonies stemming from unstressed cells not exposed to a prior sublethal heat shock (Fig 4A–4C), indicating the previously mounted heat shock response had faded to levels unable to affect the overall survival frequency [43,44]. Interestingly, however, comparison of the average survival of PA-free cells to that of PA-bearing cells clearly revealed the latter to display a significantly higher survival frequency (Student *t* test, *p*-value = 1.33 × 10^−3^; Fig 4D). Moreover, by binning the frequency of survival by average cellular IbpA-msfGFP fluorescence before the second heat shock, it also became obvious that cells bearing the highest initial IbpA concentrations (i.e. typically containing a larger PA) tend to display significantly higher survival probabilities (from an approximately 1/2 to 4/5 chance to survive; Fig 4E). Even within sister-cell pairs consisting of a PA-free and PA-bearing cell, a similar differentiation could already be observed (survival frequencies of 58.3% and 74.4% for PA-free and PA-bearing cells, respectively), further underscoring the close link of this increased robustness to PA inheritance.

Another potential explanation for these observations could stem from a confounding factor underlying both increased robustness and PA inheritance. Given the asymmetric nature of the latter phenomenon, increased cell age could be such a biasing property. Although PA-bearing cells were, on average, indeed older than the remainder of the population (average cell age of 1.80 versus 3.44 generations for PA-free and PA-bearing cells, respectively), no overall tendency for older cells to display increased survival probabilities could be detected (S6A Fig). Moreover, by exploiting the stochastic partitioning of PAs during the first generation post sublethal heat treatment, which gave rise to PA-bearing and PA-free old pole cells, we could directly disentangle the effect of both phenomena on survival. In line with our previous findings in microcolony-level semilethal survival assays [42], we found no evidence for any age-related bias in survival frequencies of PA-free cells (S6B Fig), further strengthening the role of PAs as deterministic factors that increase robustness of individual cells in an otherwise stochastic assay.

In line with our previous observations with more severe heat shocks (Fig 2), no new, distinct PAs emerged in cells surviving the semilethal heat shock, although in cells already containing a PA, this structure itself mostly remained present (Fig 4A). Again, an initial “patchy” localization pattern and a belated up-regulation of IbpA together with its apparent dissolution could be observed (Fig 4A). A similar observation could be made in surviving cells of microcolonies consisting of unstressed cells not exposed to a prior heat shock (Fig 4B).

Taken together, these results indicate that heat-induced PAs are able to increase the survival probability of individual cells upon exposure to a subsequent heat shock. As such, PAs appear to function as asymmetrically segregating, epigenetic memory elements (i.e., reminiscing previous torments) that impose phenotypic heterogeneity in isogenic microcolonies by improving the robustness of the PA-inheriting siblings. Moreover, detailed lineage tracing after the semilethal heat shock revealed that the increase in robustness might not only manifest itself in terms of higher survival probabilities but also in terms of decreased resuscitation times. Surviving PA-bearing cells appeared to resume growth and division faster than other surviving cells, leading to an enrichment of their progeny in the emerging population (Fig 4F).

### Development and validation of a novel synthetic prokaryotic PA model system

**Fig 5:**
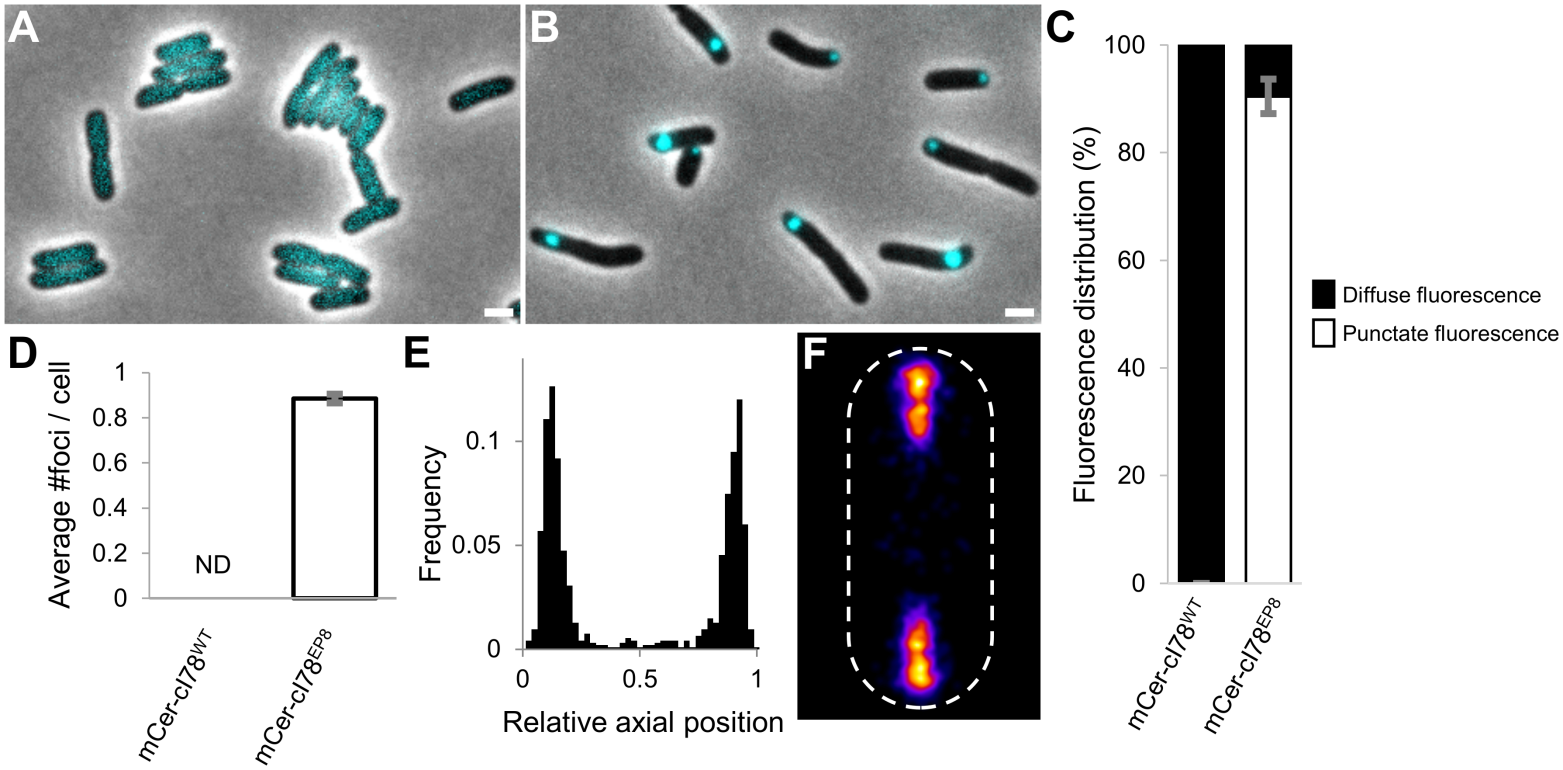
Development of a novel synthetic PA model system in *E*. *coli*. (A-B) Representative microscopic images of exponential (A) MG1655 Δ*lacY* pTrc99A-*mCer-cI78*^*WT*^ and (B) MG1655 Δ*lacY* pTrc99A-*mCer-cI78*^*EP8*^ cells. Phase-contrast images are superimposed with CFP epifluorescence images (reporting mCer-cI78 localization), and the scale bar corresponds to 2 μm. (C) Measured distribution of punctate and diffuse fluorescence intensity for the indicated fusion proteins. The means of 3 independent experiments are shown, with error bars representing the standard deviation between experiments. The fluorescence intensity distribution of 15 individual cells was determined per experiment. (D) The average number of observed foci per cell for the indicated fusion proteins. The means of 3 independent experiments are shown, with error bars representing the standard deviation between experiments. Per experiment, at least 50 cells were examined to determine the average number of cellular foci. ND = no foci could be detected. (E) Binned histogram showing foci distribution along the relative axial position of MG1655 Δ*lacY* pTrc99A-*mCer-cI78*^*EP8*^ cells (*n* = 949 cells). (F) Heat map displaying the localization pattern of foci in these cells (*n* = 3,233 cells). The numerical data underlying this figure can be found in S1 Data. CFP, cyan fluorescent protein; mCer, monomeric cerulean; PA, protein aggregate.

To independently confirm that intracellular PAs constitute an asymmetrically transmissible form of epigenetic memory that drives heat resistance, we set forward to develop a novel synthetic PA model system that alleviates the need for an external stress factor to induce PA formation. More specifically, we started with a fragment of the lambda prophage repressor protein (cI), a protein known for its potential to misfold and aggregate by the introduction of a limited number of mutations [45]. To ensure the inertness of the repressor within the *E*. *coli* cytoplasm, its N-terminal DNA-binding domain was first removed [46]. The obtained fragment, dubbed cI78^WT^ (as it does not include the first 77 amino acids of the full-length repressor protein), was fused N-terminally to the mCerulean3 fluorescent protein (yielding mCer-cI78^WT^) and placed under the control of an isopropyl β-D-1-thiogalactopyranoside (IPTG)-inducible promoter on a pTrc99A expression vector (yielding pTrc99A-*mCer-cI78*^*WT*^*)*. This construct was subsequently transformed into an MG1655 Δ*lacY* strain to allow the titratable expression of the fusion protein [47]. Upon induction, this fluorescent fusion protein displayed a diffuse cytosolic fluorescence, indicating the protein remained completely soluble (Fig 5A). To isolate constitutively aggregating mCer-cI78 variants, we screened for mutations in cI78, introduced by error-prone PCR, that resulted in an alteration of this diffuse fluorescent localization pattern. As such, we were able to identify 1 mutant (cI78^EP8^; harboring 1 synonymous and 3 nonsynonymous mutations) displaying strict punctate and polarly located fluorescence (Fig 5B–5F), characteristic of disrupted folding and subsequent aggregation.

To further validate this isolated mutant as an aggregating protein, we examined whether it displayed other typical properties of prokaryotic PAs. First, we investigated whether its polar localization was nucleoid-enforced by equipping our MG1655 Δ*lacY* pTrc99A-*mCer-cI78*^*EP8*^ strain with a *hupA-Venus* fusion, allowing the fluorescent visualization of the nucleoid [48], as well as by examining the behavior of mCer-cI78^EP8^ in the absence of a nucleoid (by employing a Δ*recA* derivative of MG1655 Δ*lacY* pTrc99A-*mCer-cI78*^*EP8*^, which occasionally gives rise to anucleate cells [49]). In agreement with characteristic PA behavior, mCer-cI78^EP8^ foci localized to nucleoid-free regions of the cell and were retained in the cell poles by nucleoid occlusion (evidenced by foci freely roaming throughout the entire cytoplasm in anucleate cells; S7A–S7C Fig). Second, we equipped mCer-cI78^WT^- and mCer-cI78^EP8^-expressing strains with pSG1 and confirmed that *ibpA* expression under inducing conditions indeed increased in the latter (S7D Fig). In addition, we equipped MG1655 *ibpA-msfgfp* cells with a pTrc99A-*mCherry-cI78*^*EP8*^ construct (in which the mCerulean3 fluorescent protein was replaced by mCherry because of its spectral incompatibility with msfGFP) and examined whether IbpA was able to recognize the misfolding and aggregating cI78^EP8^ variant. From this, a clear colocalization pattern between the IbpA-msfGFP and mCherry-cI78^EP8^ foci could be observed, indicating the latter indeed represents a misfolded and aggregating protein species (recognized by IbpA; S7E and S7F Fig).

We subsequently investigated whether the production of the mCer-cI78^EP8^ aggregating protein, as is the case with other misfolding protein species [10,50], affected cellular fitness. Whereas no difference in fitness could be detected in uninduced conditions (without the addition of IPTG), cells actively producing the mCer-cI78^EP8^ variant grew significantly slower than the cells producing the mCer-cI78^WT^ variant, with the fitness disadvantage attributable to PAs increasing with the amount of insoluble protein produced (S7G Fig). Moreover, in fully induced conditions (1 mM IPTG), a clear negative correlation between microcolony fluorescence (indicative of the amount of insoluble protein present) and microcolony growth rate could be observed for cells expressing the aggregating mCer-cI78^EP8^ variant (S7H Fig). A similar negative correlation could not be detected in cells expressing the soluble cI78^WT^ variant (S7I Fig), indicating the fitness defect was indeed attributable to misfolding and aggregation of the mCer-cI78^EP8^ protein. Please note that the observed fitness defect under these conditions, in which the misfolding protein is actively being produced, is fundamentally different from the previously reported absence of a fitness defect for host cells of asymmetrically segregating PAs after exposure to a sublethal heat treatment (Fig 3G). Whereas the former represents cells that are continuously challenged by misfolding proteins, environmental proteotoxic stress conditions have been relieved in the latter, and the PAs only remain present as remnants of a previously encountered sublethal proteotoxic insult.

### Synthetic PAs encode artificial memory and confer increased heat resistance

**Fig 6:**
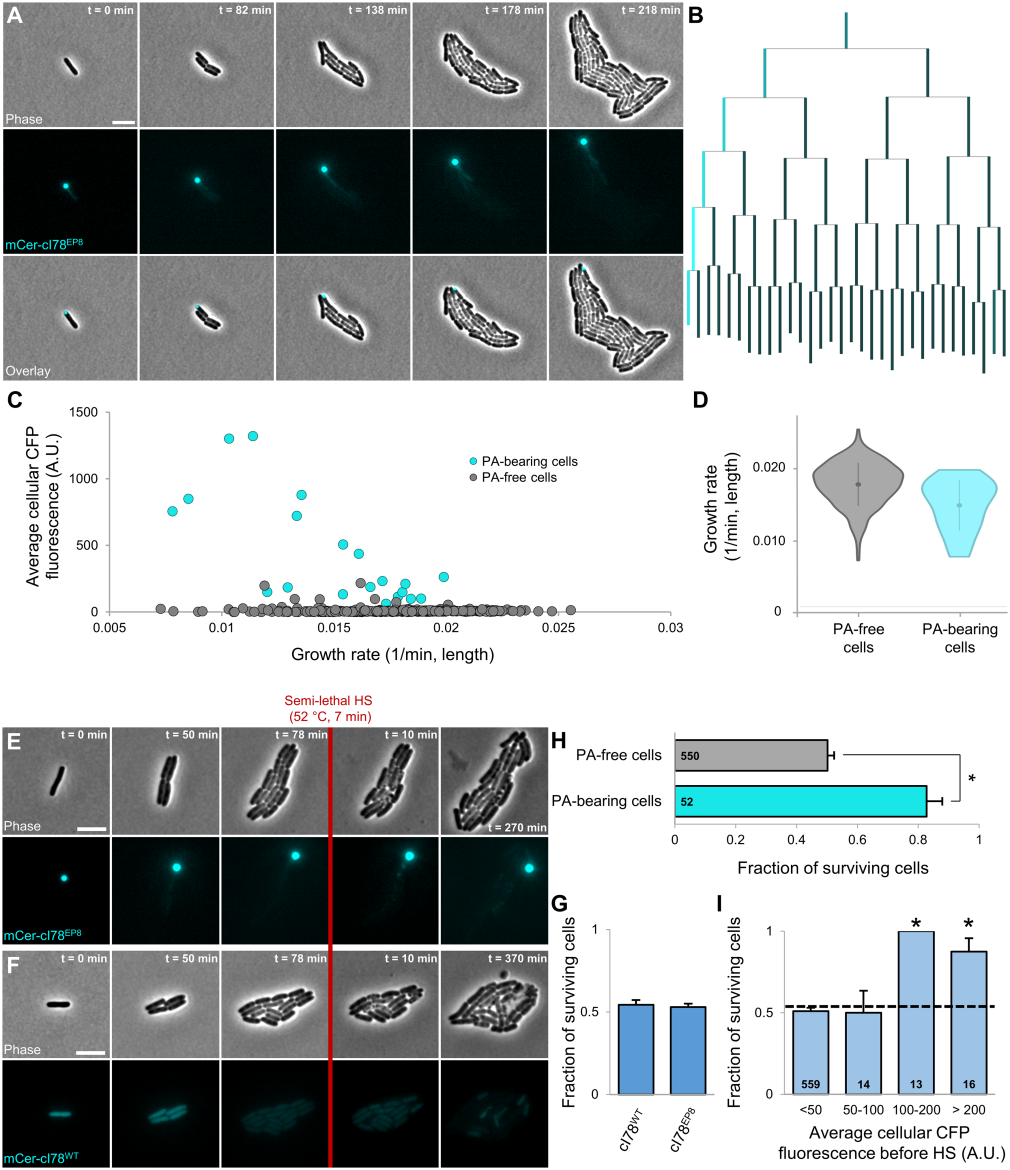
Asymmetric segregation and concurrent disaggregation of synthetic PAs encode artificial memory. (A) Representative phase contrast, CFP epifluorescence (reporting mCer-cI78^EP8^ concentration and localization), and superimposed images of a TLFM image sequence of MG1655 Δ*lacY* pTrc99A-*mCer-cI78*^*EP8*^ cells growing after induction of the aggregation-prone and PA-forming mCer-cI78^EP8^ was halted. Scale bars correspond to 5 μm. (B) Corresponding lineage tree in which the length of lines connecting cells to their progeny is proportional to the relative growth rate of that cell, and the color of these lines is proportional to the relative average CFP fluorescence of that cell. At each division except the first—in which the identity of the poles is not known, and cells are positioned randomly—the cell inheriting the old pole is placed on the left side of the division pair. (C) Correlation between average cellular CFP fluorescence and growth rate of individual fourth- and fifth-generation MG1655 Δ*lacY* pTrc99A-*mCer-cI78*^*EP8*^ cells (*n*_microlonies_ = 22, *n*_cells_ = 384) after induction was halted. Color indicates whether the respective cell was PA-bearing or PA-free. Overall, no strong correlation could be observed between both variables (Pearson’s r = 0.2886, *p*-value = 2.33 × 10^−8^), although within PA-bearing cells, a stronger correlation could be observed (Pearson’s r = 0.7242, *p*-value = 3.37 × 10^−4^, *n* = 20). (D) Violin plots comparing the distribution of growth rates of the same PA-free and PA-bearing cells as in (C) (*n* = 364 and *n* = 20 cells, respectively). (E-F) Representative phase contrast and CFP epifluorescence images of a TLFM image sequence of growing (E) MG1655 Δ*lacY* pTrc99A-*mCer-cI78*^*EP8*^ and (F) MG1655 Δ*lacY* pTrc99A-*mCer-cI78*^*WT*^ cells after induction was halted, before and after application of an HS (52 °C, 7 min) killing approximately half of the cells. Scale bars correspond to 5 μm. (G) Average survival probability of MG1655 Δ*lacY* pTrc99A-*mCer-cI78*^*WT*^ and MG1655 Δ*lacY* pTrc99A-*mCer-cI78*^*EP8*^ cells (*n*_microlonies_ = 21, *n*_cells_ = 437 and *n*_microlonies_ = 47, *n*_cells_ = 602, respectively). No significant difference could be detected in survival frequency (Student *t* test, *p*-value = 0.69). (H) Survival probability of PA-free and PA-bearing MG1655 Δ*lacY* pTrc99A-*mCer-cI78*^*EP8*^ (*n* = 550 and *n* = 52 cells, respectively) upon exposure to the HS (52 °C, 7 min). Asterisk indicates a significant difference in survival frequency between both cellular classes (Student *t* test, *p*-value = 2.83 × 10^−7^). (I) For PA-containing microcolonies MG1655 Δ*lacY* pTrc99A-*mCer-cI78*^*EP8*^ cells, the fraction of cells surviving the HS (52 °C, 7 min) is binned by the average cellular fluorescence before this HS. The dotted line indicates average survival of all cells; asterisks indicate bins that significantly differ (Fisher’s exact test, *p*-values = 3.00 × 10^−4^ and 8.80 × 10^−3^). Numbers in black indicate the number of cells included in each bin. Error bars for (H)–(I) indicate bootstrapped estimates of the standard error of the mean fraction of surviving cells. The numerical data underlying this figure can be found in S1 Data. A.U., arbitrary unit; CFP, cyan fluorescent protein; HS, heat shock; mCer, monomeric cerulean; PA, protein aggregate; TLFM, time-lapse fluorescent microscopy.

Using this newly developed and validated model system, we subsequently examined whether synthetic PAs (consisting of an aggregating, inert *E*. *coli* protein) indeed confer increased heat resistance. In a first step, MG1655 Δ*lacY* pTrc99A-*mCer-cI78*^*EP8*^ cells were grown to exponential phase in AB medium with 0.2% glycerol in the presence of 1 mM IPTG (to induce the production of mCer-cI78^EP8^), harvested, and washed into AB medium with 0.2% glucose (impeding further induction of mCer-cI78^EP8^ production), after which their growth was monitored by TLFM. Similarly as in *MG1655 ibpA-msfgfp* cells after a sublethal heat shock, the synthetic PAs not only segregated asymmetrically throughout the emerging microcolonies but were also accompanied by a mCer-cI78^EP8^ concentration gradient originating from the PA-bearing cells (Fig 6A and 6B and S3 Movie). In this case, given that production of the fluorescent protein has been halted, it is clear that the emerging concentration gradient is a consequence of protein disaggregation rather than a specific response to the presence of PAs. Although PA-bearing cells, on average, displayed a significant fitness defect (16.3%; Fig 6A–6D), this effect could not easily be discerned from cellular aging, as both phenomena were similar in magnitude (16.1% decrease in cellular growth rate of cells inheriting the oldest cell poles compared to the rest of the population; S2K Fig), and a larger fraction of old pole cells also contained a PA (13 out of 24 observed old pole cells). Nevertheless, a direct comparison of the growth rates of PA-bearing old pole cells with their PA-free counterparts yielded no significant difference (Student *t* test, *p*-value = 8.25 × 10^−2^), indicating that the contribution of PAs to the observed cellular aging was again negligible.

**Fig 7:**
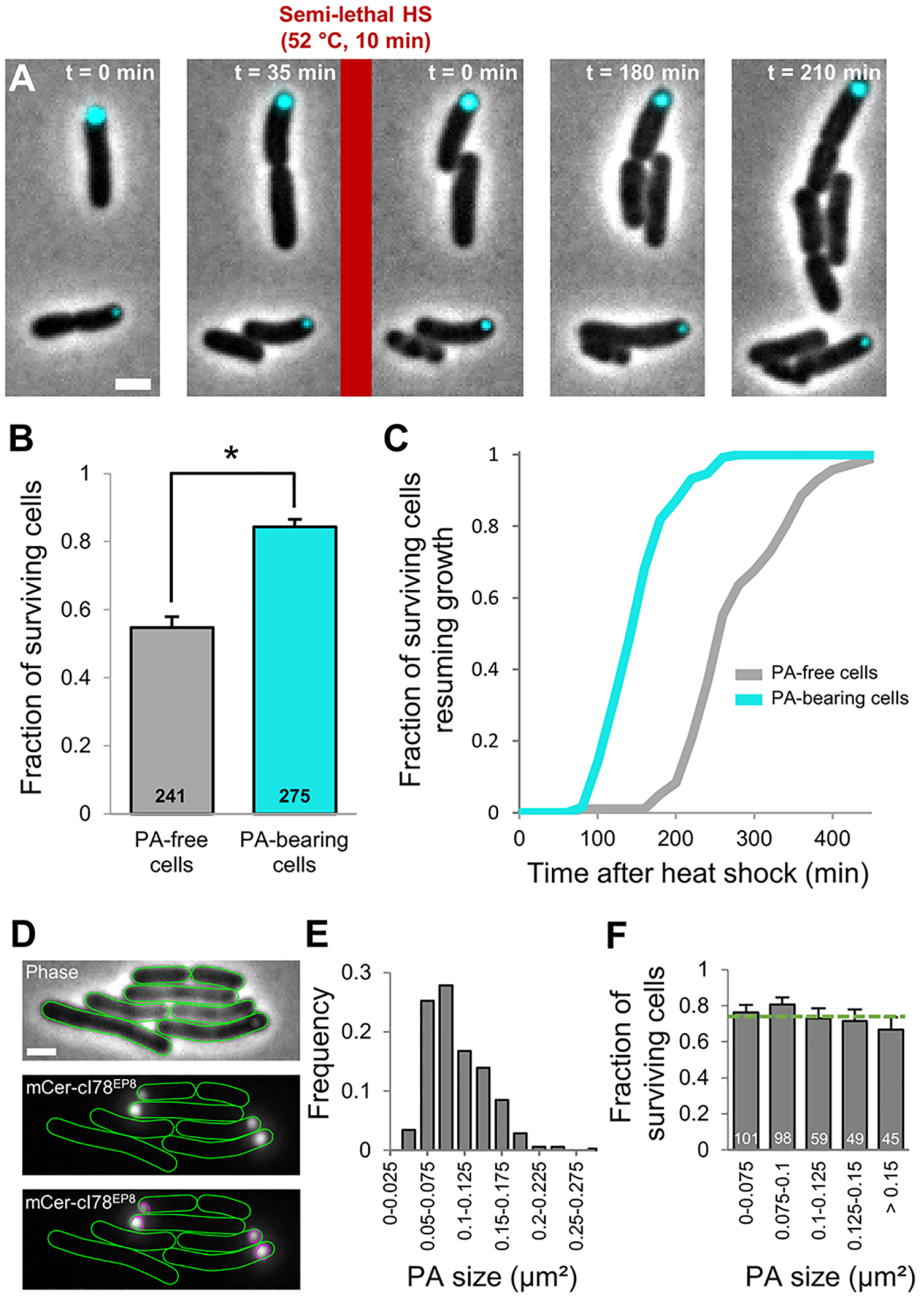
Rapid manifestation of PA-induced heterogeneity in terms of survival probability and resuscitation time. (A) Representative phase contrast images superimposed with CFP epifluorescence images (reporting mCer-cI78^EP8^ concentration and localization) of a TLFM image sequence of MG1655 Δ*lacY* pTrc99A-*mCer-cI78*^*EP8*^ cells before and after application of a semilethal HS (52 °C, 10 min). Scale bar corresponds to 2 μm. (B) Survival probability of PA-free and PA-bearing cells within sibling pairs (*n* = 258 pairs) containing at least 1 PA upon application of the HS (52 °C, 10 min). Asterisk indicates a significant difference could be detected between the survival probability of both classes (Student *t* test, *p*-value = 1.77 × 10^−13^). Numbers in black indicate the number of cells included in each group. (C) Cumulative resuscitation time distributions of PA-bearing and PA-free MG1655 Δ*lacY* pTrc99A-*mCer-cI78*^*EP8*^ cells (*n* = 133 and *n* = 96, respectively) surviving the heat treatment (52 °C, 10 min). The time individual surviving cells needed to resume growth was determined and binned to create the resuscitation time distributions. Surviving PA-bearing cells displayed significantly shorter resuscitation times than their surviving PA-free counterparts (K-S test, *p*-value = 1.17 × 10^−31^). (D) PAs were detected as non-diffraction-limited spots in the CFP epifluorescence image, their size was determined and binned to create the PA size distribution. Representative phase contrast, CFP epifluorescence, and CFP epifluorescence images with detected regions are shown. Scale bar corresponds to 2 μm. (E) Distribution of PA sizes. (F) Survival probability of cells (stemming from the PA-bearing cells shown in (B)) harboring PAs of different sizes (*n* = 352 cells). No significant difference in survival probability could be detected when compared to the average cellular survival (indicated by the dotted green line; Fisher’s exact test). Numbers in white indicate the number of cells included in each bin. The numerical data underlying this figure can be found in S1 Data. CFP, cyan fluorescent protein; HS, heat shock; K-S, Kolmogorov-Smirnov; mCer, monomeric cerulean; PA, protein aggregate; TLFM, time-lapse fluorescent protein.

Upon exposure of these growing microcolonies to a heat shock killing approximately half of the population (52 °C, 7 min), the synthetic PAs were found to confer a similar protective effect as seen with the IbpA-msfGFP-labeled stress-induced PAs (Fig 6E–6I). While average cellular survival did not significantly differ between PA-harboring (MG1655 Δ*lacY* pTrc99A-*mCer-cI78*^*EP8*^) and PA-free (MG1655 Δ*lacY* pTrc99A-*mCer-cI78*^*WT*^) microcolonies (Fig 6G), PA-bearing cells displayed a significantly higher survival frequency within the former group (Fig 6H and 6I). Remarkably, this synthetic PA-mediated protection already manifested itself after one division (Fig 7A and 7B), underscoring the apparent speed with which this PA-mediated differentiation occurs. Upon further analysis, this experimental setup clearly revealed the dual effect of PA presence on stress management, as PA-bearing cells not only displayed a higher survival frequency, but surviving PA-bearing cells also displayed a significantly reduced resuscitation time as compared to their surviving PA-free counterparts (Fig 7C).

Synthetic PAs appeared as non-diffraction-limited fluorescent spots, which allowed the accurate determination of their size (instead of relying on indirect, and potentially biased, fluorescence intensity measurements, see Materials and methods; Fig 7D and 7E and S8 Fig). These measurements revealed that aggregate size did not appear to correlate with cellular survival, indicating that the amount of aggregated protein species is, at least within the sampled range, irrelevant for the observed phenotype (Fig 7F). Moreover, these measurements allowed us to estimate the copy number of the mCer-cI78^EP8^ molecules in each aggregate. Based on its amino acid sequence, the fusion protein has an approximate molecular weight of 45 kDa and is estimated to occupy a volume of 5.71 × 10^−8^ μm^3^ [51]. Assuming spherical aggregate shape and a pure mCer-cI78^EP8^ composition, individual synthetic aggregates are subsequently roughly estimated to be composed of around 4 × 10^5^ protein molecules.

Cells actively producing misfolded and aggregating protein species became more susceptible to heat stress than those producing similar amounts of soluble protein (S9 Fig). This was illustrated by the decreased survival of MG1655 Δ*lacY* pTrc99A-*mCer-cI78*^*EP8*^ populations as compared to that of MG1655 Δ*lacY* pTrc99A-*mCer-cI78*^*WT*^ populations after exposure to a semilethal heat treatment in inducing conditions (S9 Fig). The protective effect of PAs thus appears limited to cases in which the original conditions giving rise to their emergence have been relieved. Together with the previously described negative impact of PA production on cellular fitness (S7G–S7I Fig), this sensitization toward proteotoxic stress during PA production likely impedes PA formation from being an efficient way to increase average population-level robustness in benign conditions (without a previous sublethal proteotoxic exposure). As such, PAs likely function as true epigenetic memory elements, in which cells must have encountered an environmental condition licensing their emergence.

### PAs confer robustness over a wide range of temperatures, over longer timescales, and against other proteotoxic stressors

**Fig 8:**
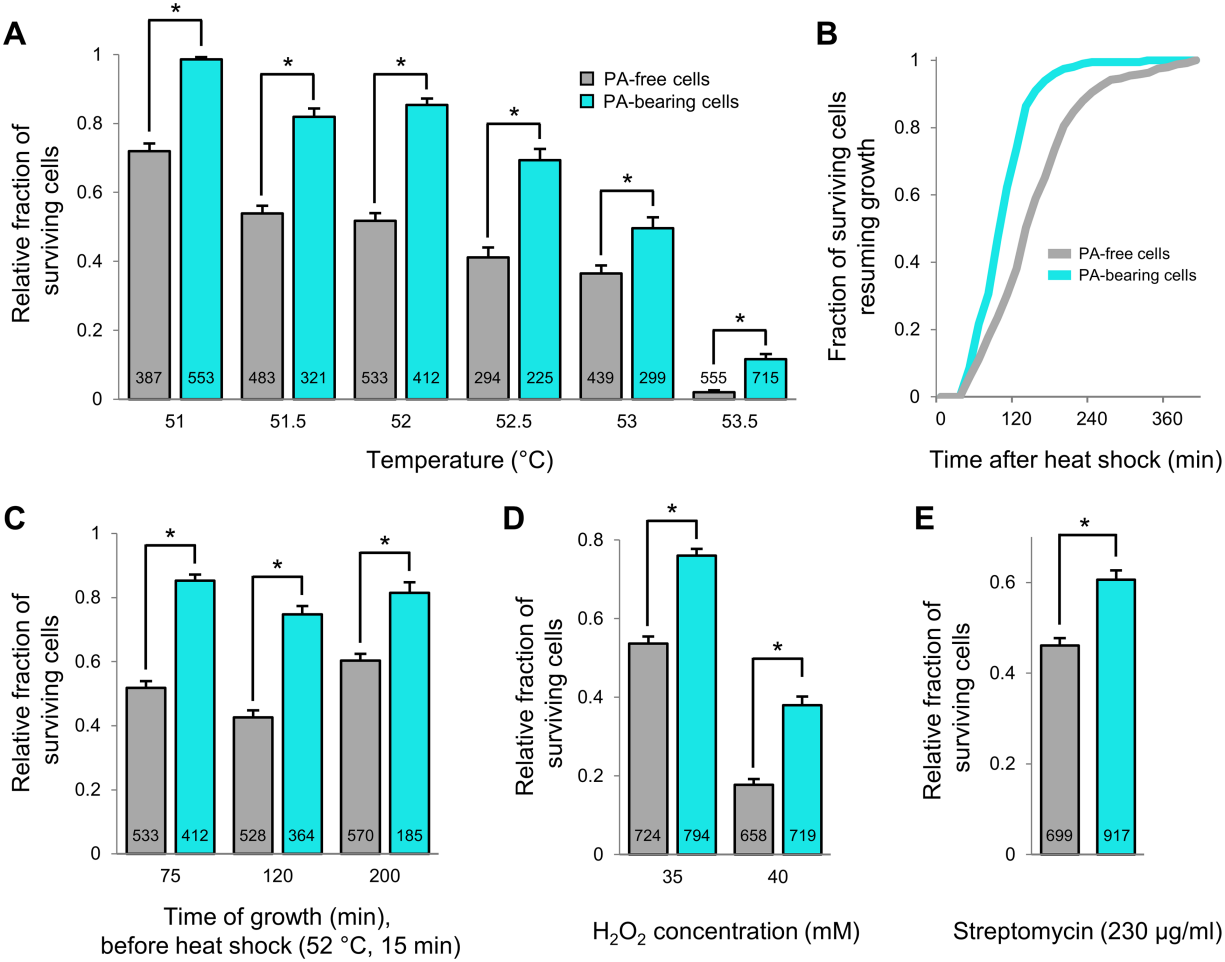
PAs encode robustness to different stress intensities, over longer timescales, and to other proteotoxic stressors. (A) Relative survival probability of PA-free and PA-bearing MG1655 Δ*lacY* pTrc99A-*mCer-cI78*^*EP8*^ cells upon exposure to the indicated range of heat intensities (15 min). Asterisks indicate a significant difference in survival frequency between the two groups (Student *t* test, *p*-values = 2.64 × 10^−25^, 7.97 × 10^−17^, 7.31 × 10^−29^, 5.03 × 10^−10^, 8.39 × 10^−4^, and 2.47 × 10^−9^, respectively). (B) Cumulative resuscitation time distributions of PA-bearing and PA-free MG1655 Δ*lacY* pTrc99A-*mCer-cI78*^*EP8*^ cells (*n* = 199 and *n* = 241, respectively) surviving the heat treatment of 51 °C (15 min). The time individual surviving cells needed to resume growth was determined and binned to create the resuscitation time distributions. Surviving PA-bearing cells displayed significantly shorter resuscitation times than their surviving PA-free counterparts (K-S test, *p*-value = 3.67 × 10^−13^). (C) Relative survival probability of PA-free and PA-bearing MG1655 Δ*lacY* pTrc99A-*mCer-cI78*^*EP8*^ cells upon exposure to a heat shock (52 °C, 15 min) after varying times of growth after PA production was halted. Asterisks indicate a significant difference in survival frequency between the two groups (Student *t* test, *p*-values = 2.64 × 10^−25^, 2.49 × 10^−20^, and 1.99 × 10^−7^, respectively). (D) Relative survival probability of PA-free and PA-bearing MG1655 Δ*lacY* pTrc99A-*mCer-cI78*^*EP8*^ cells upon exposure to hydrogen peroxide (35 and 40 mM, 60 min). Asterisks indicate a significant difference in survival frequency between the two groups (Student *t* test, *p*-values = 5.07 × 10^−15^ and 1.16 × 10^−17^, respectively). (E) Relative survival probability of PA-free and PA-bearing MG1655 Δ*lacY* pTrc99A-*mCer-cI78*^*EP8*^ cells upon exposure to streptomycin (230 μg/ml, 60 min). Asterisk indicates a significant difference in survival frequency between the two groups (Student *t* test, *p*-value = 4.09 × 10^−8^). For (A), (C), (D), and (E), numbers in black indicate the number of cells included in each bin. Error bars indicate bootstrapped estimates of the standard error of the mean fraction of surviving cells. The numerical data underlying this figure can be found in S1 Data. K-S, Kolmogorov-Smirnov; PA, protein aggregate.

In order to further substantiate and characterize the observed PA-mediated robustness in a more versatile fashion, we used an alternative approach to determine survival frequencies of large numbers of PA-bearing and PA-free cells. In essence, after transient induction of synthetic (mCer-cI78^EP8^) PA production, the population continued growth and concomitant asymmetric PA segregation in liquid culture, after which the resulting PA-free and PA-bearing cells were stress-challenged and only then monitored on a single-cell level by TLFM (Fig 8A). By postponing the mounting and incubation of cells under the microscope, this setup allowed us to easily (i) increase the experimental throughput, (ii) vary the number of generations/segregations between PA production and stress challenge, (iii) expose the cells to other stresses than heat, and (iv) expose the cells more homogeneously to a stress as well.

First, we varied the intensity of the heat challenge and exposed clonal populations of PA-free and PA-bearing siblings to heat shocks ranging from 51 to 53.5 °C. As such, we found that PA-bearing cells consistently displayed higher survival frequencies than PA-free cells (Fig 8A), confirming the protective effect of PAs and indicating a wide protective range. Moreover, the relative increase in survival that could be attributed to PA presence generally increased with increasing heat intensity. For example, after an exposure to 53.5 °C (for 15 min), PA-bearing cells displayed an almost 6-fold higher survival frequency than PA-free cells (11.6% versus 2.0%). Even after the mildest exposure (51 °C for 15 min), PA-bearing survivors displayed significantly shorter resuscitation times than their PA-free counterparts (Fig 8B), again underscoring the dual protective effect of PAs.

Subsequently, we used the above-described approach to investigate PA-mediated robustness over longer timescales of asymmetric PA inheritance. While we previously employed timescales corresponding to around 4 generations or fewer (Figs 4, 6 and 7), we could now allow PA-containing populations to grow for longer periods of time before applying a heat challenge (52 °C for 15 min) and quantify the resulting survival of clonal PA-free and PA-bearing siblings. Although the ongoing asymmetric segregation of PAs during prolonged growth inevitably leads to a progressively decreasing fraction of PA-bearing cells, a sufficient number could still be observed to irrefutably demonstrate that the protective effect of PAs persists over longer timescales (Fig 8C). Even after 200 min of growth (corresponding to approximately 7–8 generations of growth), the presence of these structures was associated with a similar increase in survival frequency (Fig 8C).

Finally, this setup also enabled us to investigate whether the PA-mediated increase in cellular robustness extended to proteotoxic stresses other than heat. To this end, we exposed clonal populations of PA-free and PA-bearing siblings to semilethal concentrations of either hydrogen peroxide (Fig 8D) or the ribosome-targeting antibiotic streptomycin (Fig 8E). In line with the previous heat shock experiments, we found that cells harboring a PA consistently displayed higher survival frequencies for both challenges (Fig 8D and 8E), suggesting that PAs can confer robustness to a wide variety of proteotoxic stresses.

### Asymmetric segregation of PAs drives asymmetric inheritance of protein quality control components

In a subsequent step, we sought to provide some insight into the molecular mechanisms underlying the observed increase in robustness of PA-bearing cells. Given the apparent absence of a direct link between the identity of the aggregated protein species and the memory encoded by them (as is the case for other examples of protein-based inheritance [52,53]), we chose to characterize and examine the potential role of protein quality control components, as previous work has demonstrated their physical association with (disaggregating) PAs [14,54–56].

In a first step, we constructed deletion strains of *ibpA*, *ibpB*, and the entire *ibpAB* operon and equipped these with our synthetic PA system. We subsequently probed for the existence of PA-mediated robustness in these strains by using a similar setup as in Fig 6, in which growing and PA-containing microcolonies were exposed to a semilethal heat shock (52 °C, 7 min). None of the deletions, however, appeared to affect the increase in heat resistance of PA-bearing cells (S10 Fig), suggesting these small heat shock proteins do not play a role in establishing PA-mediated asymmetry.

In contrast to the small heat shock proteins, deletion of many other protein quality components is known to severely compromise heat survival [57,58]. This is further exemplified by the lack of detectable survivors in a Δ*clpB* strain in our survival assay. (S10 Fig; ClpB is a heat shock protein 100 [Hsp100] AAA+ chaperone involved in protein disaggregation [59]). This reduction in survival frequency impedes direct interpretations concerning the potential role of ClpB (and other protein quality control components) in establishing PA-mediated robustness. To overcome this confounding issue and obtain some insight into the potential role of the proteostasis network, we resorted to the use of fluorescent fusion proteins. We constructed both transcriptional and translational msfGFP fusions to a variety of chaperones and proteases and equipped these strains with the mCherry version of our synthetic PA system (to ensure spectral compatibility with msfGFP). As we expected nonfunctional fusions to decrease heat survival frequency [57,58], we first validated the fusion proteins by exposing the corresponding strains to a heat treatment and comparing their inactivation levels to those of a control without any chaperone or protease fusions (S11A and S11B Fig). This analysis revealed that only the transcriptional P_*clpP*_*-msfgfp* fusion led to a significantly increased inactivation (S11A and S11B Fig) and thus likely exerts a polar effect that perturbs wild-type cellular physiology. We further validated the transcriptional fusions by exposing these strains to a sublethal heat shock and verifying the expected increase in promoter activity ([60]; S11C Fig). Whereas most transcriptional fusions indeed displayed a significant up-regulation directly after heat treatment (S11C Fig), the transcriptional P_*clpP*_*-msfgfp* fusion did not, further supporting the lack of functionality of this fusion. A similar increase in concentration after exposure to a sublethal heat shock could often not be detected on a protein level for these chaperones and proteases (S11D Fig), suggesting the potential existence of posttranscriptional regulation, slow protein folding and maturation, and/or high rates of protein turnover.

In line with the previously noted absence of a heat shock response to the presence of asymmetrically segregating PAs (S4 Fig), we found that none of the tested protein quality control components displayed a transcriptional up-regulation in the presence of PAs (S12A and S12B Fig). Their expression level (indirectly measured through the average cellular GFP concentration) often even appeared lower than that of PA-free cells, although this likely is a consequence of the impermeability of (synthetic) PAs to other cytosolic components (S12C Fig). The presence of PAs therefore appears to lead to an extra addition of cell volume containing no fluorescence, in turn leading to a lower apparent concentration.

**Fig 9:**
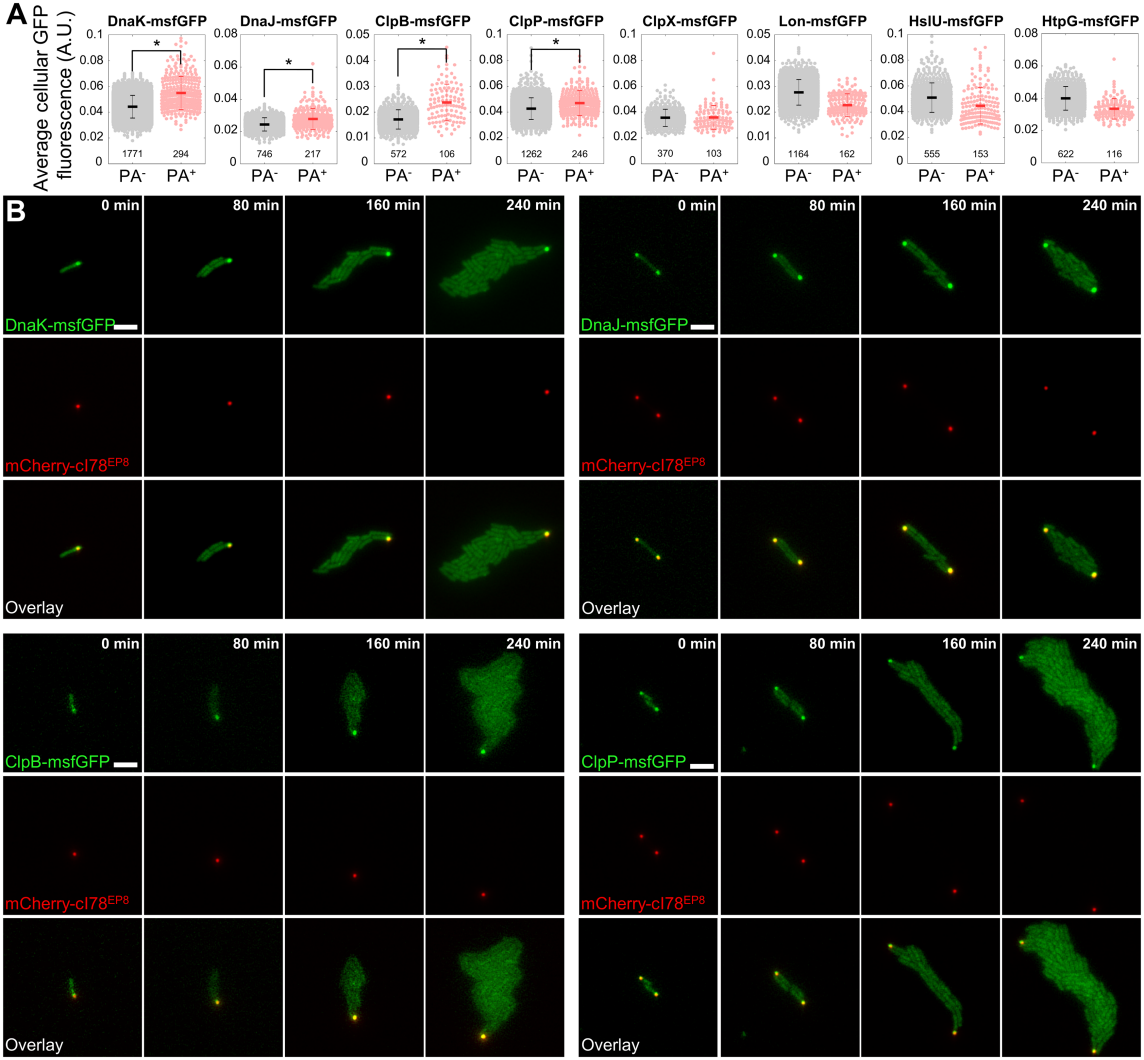
Colocalization and enrichment of specific protein quality control components in PA-bearing cells. (A) Quantification of the concentration of indicated protein quality control components (as measured by average cellular GFP fluorescence) in PA-free (PA^−^) and PA-bearing (PA^+^) MG1655 Δ*lacY* pTrc99A-*mCherry-cI78*^*EP8*^ cells harboring the indicated translational reporter, 3 h after PA production was halted. Asterisks indicate a significant increase in average protein concentration in PA-bearing cells (directional Student *t* test, *p*-values = 8.11 × 10^−35^, 4.50 × 10^−12^, 7.79 × 10^−16^, 2.23 × 10^−10^, 3.66 × 10^−1^, 1, 1, and 1, respectively). Numbers in black indicate the number of cells included in each group. Error bars indicate the standard deviation. (B) Representative GFP epifluorescence (reporting localization and concentration of the indicated translational fusion protein), mCherry epifluorescence (reporting mCherry-cI78^EP8^ localization), and superimposed images of a TLFM image sequence of the indicated translational fusion strains growing into microcolonies after PA production was halted. Scale bar corresponds to 5 μm. The numerical data underlying this figure can be found in S1 Data. A.U., arbitrary unit; GFP, green fluorescent protein; msfGFP, monomeric superfolder GFP; PA, protein aggregate; TLFM, time-lapse fluorescence microscopy.

On the protein level, however, specific protein quality control components (the Hsp70 chaperone DnaK, the Hsp40 chaperone DnaJ, the Hsp100 AAA+ chaperone ClpB, and the serine protease ClpP) displayed an increased concentration in PA-bearing cells (Fig 9A). TLFM revealed that the increased concentration was a direct consequence of a remarkable colocalization of these proteins with asymmetrically segregating PAs that persisted over multiple generations (Fig 9B). This observation appears to be in line with the similarly ongoing PA-disaggregation observed earlier (Figs 3A, 3C, 4A and 6A). Other protein quality components (the AAA+ protease subunit ClpX, the serine protease Lon, the AAA+ protease subunit HslU, and the Hsp90 chaperone HtpG) did not display any colocalization or an increased concentration (Fig 9A and S13 Fig). In fact, in similar fashion as for the transcriptional reporters and presumably because of the same PA-mediated addition of nonfluorescent cell volume, a lower apparent concentration was often measured in PA-bearing cells for these chaperones and proteases. Asymmetric segregation of PAs thus appears to drive the specific enrichment of protein quality control components in their host cells. This enrichment might in turn be responsible for the increased robustness of PA-bearing cells toward proteotoxic stresses.

## Discussion

In contrast to the governing view on prokaryotic PAs as inevitably debilitating structures [10–12,14], our findings in the *E*. *coli* model system reveal the potential of PAs to improve cellular robustness, both in terms of increased survival frequencies and decreased resuscitation times, independent of their origin. Moreover, because of their asymmetric segregation and limited disaggregation, these structures resist dilution during cytoplasmic inheritance and are therefore able to persist for many generations as a functional physical relic of an ancestral insult. As such, these observations reveal the existence of stress-induced, long-term epigenetic memory in prokaryotes.

More specifically, such cellular memory appears to be installed through (sublethal) proteotoxic exposures that lead to initial up-regulation of the heat shock response and colocalization of specific chaperones and proteases with the emerging PAs. Growth after relief of the proteotoxic exposure coincides with asymmetric segregation of this PA and its associated chaperones and proteases, while additional heat shock proteins do not seem to be induced upon the mere inheritance of this structure. Whereas the selective redistribution of quality control components to polar PAs in *E*. *coli* during and directly after proteotoxic stress has been observed before [8,10,13,14], our data indicate that this association persists over multiple generations and leads to their specific enrichment in PA-bearing cells. In fact, the slowly ongoing PA-disaggregation during outgrowth might be a consequence of a prolonged association of these components with the PA. Importantly, previous studies have reported that the association of several chaperones and proteases with PAs is dynamic [14], indicating that these proteins are not necessarily trapped within these intracellular structures and might become available as additional protein folding aids that increase cellular robustness in times of stress. Interestingly, this putative mechanism might even extrapolate to other (eukaryotic) microorganisms, since *S*. *cerevisiae* mother cells harboring an age-associated PA were previously reported to clear heat-induced aggregates faster than daughter cells without such an aggregate [61].

However, further research is required to establish a direct causal relation between PA-associated chaperones and proteases and the observed cellular robustness. While the use of individual deletion mutants does not seem a good option because of their severely compromised heat survival [57,58](S10 Fig), obtaining tunable amounts of different protein quality control components and subsequently assessing to what extent this gradually affects PA-mediated robustness might be a more fruitful strategy to establish causality. Furthermore, it will also be interesting to see whether or not PA-mediated protection is consequently limited to those stresses that require protein folding aids to be alleviated.

In essence, epigenetic memory refers to hysteretic behavior in which the physiological state of a cell is determined by its (or its ancestor’s) past experience rather than being dictated by genomic mutations or the current environment it resides in [62,63]. Nonmutational mechanisms allowing the long-term inheritance of a previously acquired physiological state are typically based on the passage of specific protein activities or interactions from one generation to the next. As such, *E*. *coli* cells already expressing the LacY permease in intermediate inducer concentrations will continue doing so for subsequent generations because their ability to import the inducer will lead to continued production of the permease [64,65]. Similarly, cells inheriting a self-propagating prion seed transmit this infectious protein conformation to their progeny, in which this process will repeat itself [66–69]. In contrast to these mechanisms, intracellular PAs essentially lack a self-proliferative effect (i.e., it is the original PA that is passed on from one cell to another) but nevertheless remain inheritable for many generations because the asymmetric segregation and limited disaggregation prevent their dilution. This relates to the mnemons concept posed by [52], in which the conditional self-aggregation of a specific protein (the Whi3 mRNA binding protein of *S*. *cerevisiae*) compromises its functionality and proper segregation to daughter cells. However, since the exact PA origin in our experiments appears to be irrelevant for its phenotypic consequences, prokaryotic PA-mediated memory seems to comprise a completely novel type of protein-based inheritance.

Our single cell–level observations also revealed other intriguing aspects of the process and impact of in vivo protein aggregation. PAs, for example, seem not to emerge by default after exposures to a proteotoxic stress. Instead, their formation appears limited to less severe, sublethal stressful encounters. Although we cannot currently provide a conclusive answer, we hypothesize that this observation could reflect different, nonmutually exclusive, cellular strategies. One possibility is that the dosage of misfolded proteins determines the choice between aggregation and refolding/degradation, given that a largely aggregated proteome would trivially lead to defects in cellular function. Another possibility is that the formation of these large intracellular structures requires a certain factor or multiple factors (in line with the observation in fission yeast that Hsp16 mediates PA fusion [70]), but this factor is allocated to repair or prevention of aggregation of other critical cellular components under more severe stress conditions. Alternatively, PA formation could pose too much of a risk under severe heat stress, as aggregation could potentially lead to coaggregation and trapping of other critical cellular components [71], themselves destabilized and misfolded under these more severe stress conditions.

Aside from their remarkable pattern of emergence, the slow and limited poststress disaggregation of PAs over multiple generations also represents cellular behavior that previously remained undetected using population-level determinations of aggregated protein fractions [8,54,72]. Although many of the molecular players and steps involved in the disaggregation process have already been identified and studied in vitro [54,56,73], our observations underscore that the in vivo implementation and kinetics of this process require further study. The resulting persistence and asymmetric inheritance of PAs over multiple generations gives rise to a progressively decreasing fraction of memory cells as population numbers increase. This heterogeneity is reminiscent of a bet-hedging strategy typically employed by microbial populations to mitigate costs or trade-offs associated with the installment of given phenotypes. However, PA-mediated fitness defects could not be detected in terms of cellular growth rate, in agreement with recent findings in fission yeast [74], suggesting such trade-offs either remain undetected in our setup (and are yet to be discovered) or are simply not present. In this light, the formal distinction between protein aggregation (believed to be deleterious) and quinary assembly formation (believed to be adaptive) [22,23] becomes more vague as well.

Importantly, microorganisms can encounter many forms of proteotoxic stress throughout their habitats, and our data indicate that sublethal exposures to streptomycin and hydrogen peroxide not only give rise to similar emergence and inheritance of PAs but that the PA-mediated robustness extends toward these other proteotoxic stresses as well. These stressors are of specific importance given their roles in curbing and fighting microbial infections in humans. Not only do many antibiotics specifically target protein homeostasis [8]; the mechanism behind the activity of bactericidal antibiotics has also been linked to the production of reactive oxygen species (ROS) [75,76]. Moreover, the generation of ROS has been implicated in microbial killing by phagocytes [77,78]. Consequently, our findings might have broader implications in the context of understanding how bacteria cope with and evade antimicrobial therapeutics and host defense mechanisms.

In conclusion, intracellular prokaryotic PAs appear to be more than inevitable and detrimental cellular garbage bins, as these structures encode epigenetic long-term memory of previous proteotoxic torments that becomes vertically transmitted for a number of generations while conferring an increased robustness to its carrier cell. As such, the formation and conservation of PAs in prokaryotes resembles an adaptive process that aids cellular survival and adaptation in fluctuating environments. Moreover, this paradigm further suggests that other types of cytoplasmically inheritable damaged biomolecules could likewise serve as functional “scar tissue” that improves cellular robustness over multiple generations.

## Materials and methods

### Strains and growth conditions

Bacterial strains, plasmids, and primers used in this study are listed in S2, S3 and S4 Tables, respectively. For culturing of bacteria, mostly lysogeny broth (LB) medium was used as either a broth or solid medium after the addition of 2% agarose (for agarose pads intended for microscopy). In indicated cases, LB medium was replaced by AB medium, supplemented with 0.2% of a carbon source (glucose or glycerol), 0.2% casamino acids, 10 μg/ml thiamine, and 25 μg/ml uracil.

Stationary-phase cultures were obtained by growing *E*. *coli* overnight for approximately 15 h in LB broth at 37 °C under well-aerated conditions (200 rpm on an orbital shaker). Exponential phase cultures were in turn prepared by diluting stationary phase cultures 1/100 in fresh prewarmed broth and allowing further incubation at 37 °C until an OD600 of 0.2–0.6 was reached. When appropriate, the following chemicals (Applichem, Darmstadt, Germany, and Sigma-Aldrich) were added to the medium at the indicated final concentrations: kanamycin (50 μg/ml), ampicillin (100 μg/ml), chloramphenicol (30 μg/ml), 4′,6-diamidino-2-phenylindole (DAPI; 1 μg/ml), IPTG (1–1,000 μM), glucose (0.2%), L-arabinose (0.1%–0.2%), and glycerol (0.2%).

### Construction of mutant strains

The construction of mutants in *E*. *coli* is greatly facilitated by lambda Red-mediated recombination. The protein products of the *red* genes (Gam, Exo, and Beta) enable the highly efficient recombination of PCR products (containing a selectable antibiotic cassette, optionally flanked by other genes of interest such as those encoding fluorescent proteins) flanked by short (50 bp) nucleotide sequences, homologous to the target sequence [79]. These antibiotic cassettes are usually flanked by *frt* sites, which allow the excision of the cassette by site-specific recombination between two *frt* sites.

All constructed mutants were initially confirmed by PCR with primer pairs attaching outside of the region where homologous recombination occurred. Correct deletion or integration of PCR products was further verified by sequencing (Macrogen, the Netherlands).

The different C-terminal translational fusions of IbpA and IbpB were constructed by recombineering PCR fragments obtained from plasmid pDHL1029 and its derivatives pDHL-*venus*, pDHL-*mVenus*, pDHL-*mCherry*, and pDHL-*mCer* (the original pDHL1029 plasmid contains a *msfgfp-frt-nptI-frt* cassette [36]; its derivatives were constructed during this work and contain sequences encoding other fluorescent proteins: Venus, mVenus [monomerized Venus by introducing V206K mutation], mCherry, mCerulean3) using primer pairs SG1-2 (IbpA) and SG3-4 (IbpB), in MG1655 creating a C-terminal fusion of IbpA and IbpB to the different fluorescent proteins. The resistance cassette was subsequently excised by transiently equipping this strain with plasmid pCP20 expressing the Flp site-specific recombinase [80], resulting in the desired strain containing a fluorescent IbpA/IbpB fusion protein.

C-terminal translational fusions of protein quality control components (DnaK/DnaJ/ClpB/ClpP/ClpX/HtpG/HslU/Lon) were constructed in similar fashion using primer pairs SG23-38. The transcriptional fusions probing their expression level were constructed by first obtaining a *msfgfp-frt-nptI-frt* amplicon from plasmid pDHL1029 using primer pairs SG39–SG54. The respective fluorescent transcriptional reporter strains were then created by recombineering this amplicon 5 bp after the stop codon of the gene of interest, creating an artificial operon and ensuring cotranscription. To maximize cotranslational activity, the gene encoding *msfgfp* was preceded by a strong synthetic ribosome binding site (BBa_B0034; sequence AAAGAGGAGAA [81]). The resistance cassette was subsequently excised by transiently equipping this strain with plasmid pCP20 expressing the Flp site-specific recombinase [80], resulting in the desired fluorescent transcriptional reporter strains.

The C-terminal translational fusion of HupA to Venus was constructed using primer pair SG5-6 and pGBKD-venus as a template.

Deletion strains were constructed using an amplicon prepared on pKD13 using the primers listed in the study by Baba and colleagues [79,82] for recombineering. This procedure replaced the genes of interest with an *frt*-flanked kanamycin resistance cassette, which could subsequently be excised by transiently equipping this strain with plasmid pCP20 [80], resulting in the desired deletion strain.

### Construction of plasmids

All constructed plasmids were verified by both PCR and sequencing (Macrogen, the Netherlands). Plasmids were introduced into their respective host strains by transformation and selection for antibiotic resistance encoded by the plasmid.

Plasmids pGBKD-*mCherry* and pGBKD-*venus* were constructed by integrating an *mCherry*/*venus* amplicon, generated from pDHL-*mCherry* and pDHL-*venus* with primer pairs SG7-8 (*mCherry*) and SG9-10 (*venus*), into pGBKDparSpMT1 [83] using EcoRI and BamHI restriction sites. In addition to adding the respective restriction sites to the end of the amplicon, these primer pairs also add a flexible linker (encoding GSGSGS; [84]), facilitating folding of fluorescent fusion proteins constructed with these sequences.

Plasmid pSG1 was constructed by first inserting an amplicon—obtained from pGBKD-*mCherry* using primer pair SG11-12—into MG1655, creating a transcriptional *ibpA* fusion in which the native *ibpA* gene was completely replaced by *mCherry*. From this, an amplicon (using primer pair SG55-56) was obtained containing the entire 5′-upstream region of *ibpA* (including its promoter and 5′-UTR) in front of *mCherry*, which was subsequently blunt-end ligated into a pACYC184 vector ([85]; opened by PCR amplifying the entire plasmid using primer pair SG13-14). The resulting plasmid, pSG1, was initially transformed to MG1655, in which it functioned as a transcriptional reporter for *ibpA* expression. The plasmid was validated by exposing MG1655 pSG1 cells to a sublethal heat treatment that, as expected, led to an increase in cellular mCherry fluorescence.

Plasmid pTrc99A-*mCer-cI78*^*WT*^ was constructed by first making an *mCer* amplicon from pDHL-*mCer* with primer pair SG15-16. These primers amplified the *mCer* encoding gene without a start or a stop codon and added a NcoI and BamHI restriction site to the ends of the amplicon. Digestion of the amplicon and pTrc99A vector [86] with these restriction enzymes allowed the subsequent ligation of the amplicon in the expression vector. After being verified by both PCR and sequencing, the created construct was digested again with BamHI and SalI restriction enzymes. Another amplicon containing a fragment of the lambda prophage repressor protein was generated from an in-house *E*. *coli* strain harboring the lambda prophage, using primer pair SG17-18. These primers amplify a fragment of the lambda repressor protein named cI78 (as it does not contain the first 77 amino acids of the normal full-length protein) and added a BamHI and SalI restriction site as well as a flexible linker (coding for GSGS) to the end of the amplicon. Subsequent digestion of this amplicon allowed its ligation into the previously constructed and digested construct. The resulting plasmid, pTrc99A-*mCer-cI78*^*WT*^, expresses a C-terminal fusion of mCerulean3 to cI78 under control of an IPTG-inducible promoter.

Plasmid pTrc99A-*mCer-cI78*^*EP8*^ is a plasmid expressing a misfolding and aggregating cI78 mutant, identified in a screen specifically aimed at obtaining such mutants. To find mutations that contribute to misfolding, we first performed random error-prone PCR mutagenesis [87] on cI78 (dubbed cI78^WT^) and ligated the obtained mutant library behind *mCer* in pTrc99A (in similar fashion as cI78^WT^). We screened for misfolding cI78 variants by examining individual mutants under the microscope for alterations of the normally diffuse fluorescent localization pattern. From this, a cI78 mutant, cI78^EP8^ (the term “EP” stemming from the error-prone PCR method used for its construction, and 8 stemming from the number of the isolated mutant), was identified, which displayed strict punctate and polarly located fluorescence, characteristic of disrupted folding and subsequent aggregation. cI78^EP8^ harbors 1 synonymous (180C>T) and 3 nonsynonymous mutations (38A>T, 135T>A, and 343T>C). Plasmid pTrc99A-*mCherry-cI78*^*EP8*^ was created by replacing the *mCer* gene by *mCherry*.

Plasmid pBAD33-*ibpA-msfgfp* was constructed by first generating an *ibpA-msfgfp* amplicon from MG1655 *ibpA-msfgfp* cells using primer pair SG19-20. These primers amplify the entire gene fusion and add a strong synthetic ribosome binding site (BBa_B0034; sequence AAAGAGGAGAA [81]) preceding the *ibpA* start codon. The amplicon was subsequently blunt-end ligated into a pBAD33 vector ([88]; opened by PCR amplifying the entire plasmid, using primer pair SG21-22). The resulting plasmid was then transformed to MG1655, in which it was used to induce expression of the fusion protein to further probe native IbpA (i.e., unlabeled) behavior (of which the resulting strain thus also carries a copy).

### TLFM

For TLFM, cell suspensions were diluted appropriately, transferred to agarose pads (containing the appropriate medium), placed on a microscopy slide, and mounted with a cover glass. A Gene Frame (Thermo Scientific) was used to hold the cover glass on the microscopy slide. TLFM was performed with a temperature controlled (Okolab, Ottaviano, Italy; 37 °C) Ti-Eclipse inverted microscope (Nikon, Champigny-sur-Marne, France) equipped with a 60× objective, a TI-CT-E motorized condenser, a YFP filter (Ex 500/24, DM 520, Em 542/27), a CFP filter (Ex 438/24, DM 458, Em 483/32), a GFP filter (Ex 472/30, Dm 495, Em 520/35), an mCherry filter (Ex 562/40, Dm 593, Em 641/75), a DAPI filter (Ex 377/50, DM 409, Em 447/60), and a CoolSnap HQ2 FireWire CCD-camera. Images were acquired at user-chosen time intervals using NIS-elements software (Nikon). During the acquisition of TLFM recordings, care was taken to prevent potential photobleaching of fluorescent molecules (i.e., the photochemical alteration of a fluorophore molecule by, for example, the prolonged exposure light of excitation wavelengths, such that it permanently is unable to fluoresce) by minimizing excitation light intensity and enlarging time intervals in between acquisition of fluorescent images. The resulting images were further handled with open source software ImageJ.

### Image analysis and quantitative analysis of single cell–level growth

Characteristics (e.g., length, area, fluorescence) of individual cells growing in/into microcolonies (and of the microcolonies themselves) were acquired using the MicrobeTracker software [89]. In order to obtain robust results, manual curation was necessary to improve automatic segmentation and tracking. The data generated by this analysis were fed into a relational database enabling its subsequent transformation (e.g., calculation of certain cellular characteristics [growth rate], establishment of genealogical relationships between cells) and mining. The distribution of fluorescent PA foci was obtained by detecting their relative localization along the cell axis using the SpotFinder tool of MicrobeTracker [89]. Heat maps displaying the distribution of intracellular PA localization were generated using MicrobeJ, an ImageJ plugin [90].

Given the relative constancy of cell width during cell cycle progression in a given environment, cell length was employed to quantify cellular growth of individual cells. Growth rates of individual cells were determined by exponential fits of cell length over time. To examine the effect of the presence of PAs on cellular growth, only growth rates of fourth- and fifth-generation cells were considered, as these cells have grown a sufficient number of generations after the removal of the corresponding PA-inducing agent (heat or 1 mM IPTG to induce expression of cI78^EP8^) but are not yet suffering from any (local) nutrient-depletion effects. To examine the effect of cI78^EP8^ production on cellular fitness, microcolony growth rates were determined in different induction regimes and compared to those of microcolonies producing cI78^WT^. Growth rates of microcolonies were determined by exponential fits of microcolony area over time (time interval: 1–3 h after beginning of recording).

The presence of cellular aging was examined by quantifying the fitness defect of the oldest cells within the fourth and fifth generation compared to all other cells of those generations. Cell age was inferred from old pole generations as introduced by Stewart and colleagues [17]. Consequently, each generation within a microcolony contained 2 oldest cells (i.e., the cells inheriting the cell original cell poles of the “founder” cell of the microcolony). Violin plots illustrating the extent of this effect were generated using a custom script in R.

### Determination of PA size

Given that incorporation of fluorescent proteins into a PA potentially compromises their structure and fluorescence [91], we determined PA sizes directly using the ObjectDetection module within the Oufti software [92], which allows the detection of non-diffraction-limited fluorescent regions. The mCerulean3 fluorescent protein emits at 475 nm, leading to an Abbe diffraction limit of 475 / (2 * 1.4) = 169.6 nm (the value 1.4 corresponds to the numerical aperture of the microscope objective). The minimum size of circular spots that can subsequently be reliably detected is π * (169.6/2)^2^ = 22591.3 nm^2^ (or approximately 0.02 μm^2^), which is significantly smaller than the smallest measured size of PAs (0.037 μm^2^). From this, it is clear that PAs, produced by the synthetic mCer-cI78^EP8^ model system, occur as non-diffraction-limited spots within individual cells.

These measurements also allowed us to directly investigate the potential correlation between PA size and average cellular fluorescence (S8 Fig). Although an overall good correlation could be observed, this correlation appears nonlinear and noisy, especially for smaller and larger PAs.

### Permutation test

The permutation test to investigate potential contributions of PAs to the observed fitness defect of their host cells (next to the age of these cells) was performed by randomly sampling PA-free cells of similar age structure (100,000 times/permutations) and comparing their average fitness to that of all other PA-free cells. A *p*-value was subsequently calculated as the proportion of sampled permutations in which the absolute difference in fitness was greater than or equal to the observed fitness defect of PA-bearing cells (as compared to all PA-free cells). For the synthetic PA system, a similar approach could unfortunately not be employed. The large fraction of old cells also bearing a PA (more than half the number of old cells) made it impossible to disentangle the potential individual contribution of both phenomena to cellular fitness defects using this approach.

### Sublethal heat treatment of liquid cultures

Fifty μl of exponential phase cells was transferred aseptically to a sterile PCR tube and heat treated for 15 min at indicated temperatures in a thermocycler (Westburg, Leusden, the Netherlands). Control samples were also transferred to PCR tubes but were kept at room temperature for 15 min. After heat treatments, samples were aseptically retrieved from the PCR tubes and subjected to TLFM as described above.

### Semilethal heat treatment of microcolonies

To examine the effect of PAs on survival frequency in heat shock experiments, the same cells were microscopically examined before and after heat treatment. To accomplish this, cells were first mounted on a microscopy slide as described above and allowed to grow for an indicated number of generations while their spatial coordinates on the slide were noted. Subsequently, the slide as a whole was subjected to a heat shock (for indicated times at indicated temperatures; heat shock durations and intensities were chosen so to inactivate approximately half of the cellular population) by taping the slide to the lid of a thermocycler (Westburg, Leusden, the Netherlands), after which the spatial coordinates were used to trace back and microscopically follow up the same cells on the heat-treated slide.

### Semilethal treatments of liquid cultures

For experiments in which cells were precultured and challenged in liquid cultures, and survival, together with potential presence of PAs, was examined microscopically, MG1655 Δ*lacY* pTrc99A-*mCer-cI78*^*EP8*^ cells were grown to exponential phase (OD600 = 0.2–0.6) in AB medium with 0.2% glycerol. PA production was subsequently induced by adding 1 mM IPTG to the medium for 1.5 h, after which the cells were harvested and washed into AB medium with 0.2% glucose. After washing, cells were incubated at 37 °C for 75 min (or longer times when indicated) and exposed to heat, peroxide, or streptomycin stress for the indicated period of time. The fractions of surviving PA-bearing and PA-free cells were subsequently determined at the single-cell level by TLFM (after washing away the peroxide or streptomycin in cases in which these stressors were employed). As PA production appeared to occasionally (10%–20%) give rise to likely anucleate, PA-bearing cells (observable as small cells with PAs filling almost their entire cytoplasm; S14 Fig), the fraction of surviving cells for both cellular classes (PA-bearing and PA-free) was compared to the fraction of outgrowing cells in unstressed control conditions to determine the relative fraction of surviving cells for each class.

### Population-level semilethal heat treatment

To examine the effect of ongoing PA production on stress sensitivity, MG1655 Δ*lacY* pTrc99A-*mCer-cI78*^*WT*^ and MG1655 Δ*lacY* pTrc99A-*mCer-cI78*^*EP8*^ cells were exposed to a semilethal heat shock (49 °C, 15 min) during mCer-CI78^WT^ (soluble) and mCer-CI78^EP8^ (i.e., PA) production (AB medium with 0.2% of glycerol in the presence of 1 mM IPTG). The surviving fraction of cells was determined through spot-plating experiments in which the appropriate dilutions of a sample were prepared in PBS and subsequently spot-plated (5 μl) on LB agar. After 24 h of incubation at 37 °C, the plates were counted, and the number of survivors in CFU per ml was determined.

### Sublethal streptomycin and hydrogen peroxide treatment

Streptomycin (10 μg/ml for 60 min or 15 μg/ml, final concentrations, for 30 min) and H_2_O_2_ (6 mM, final concentration, for 90 min) were directly added to exponentially growing MG1655 *ibpA-msfgfp* cultures. After treatments, streptomycin and H_2_O_2_ were washed away, and samples were diluted and prepared for microscopy as described above.

### Determination of viability and resuscitation time measurements

Cellular viability (i.e., the relative number of cells surviving the heat sock) was determined by TLFM. Cells that could be observed to grow and divide within an 8 h time frame after heat treatment were scored as surviving cells.

Cell meshes generated by the MicrobeTracker program were used to determine resuscitation times of individual cells, as described previously [15]. Since bacterial cells typically only elongate in the longitudinal direction, resuscitation times were measured by looking at the length increase of individual cells over time. First, an initial length was calculated as the mean of the first 3 measurements for each individual cell. The length of that cell in the subsequent frames was then compared to this initial length, and the resuscitation time was defined as the time corresponding to the frame in which cell length had increased over 10% compared to its initial length, plus the time between the end of the heat treatment and the beginning of microscopy recording (typically around 10 min). This 10% increase in initial length was taken as a threshold to prevent random measurement fluctuations from influencing the results and ensure that only resuscitation times of cells that had fully committed to growth were measured. In addition, only resuscitation times of surviving cells were measured, i.e., cells that subsequently committed to growth and division.

### Validation of transcriptional and translational msfGFP fusions of protein quality control components

As we expected nonfunctional fusions to compromise cellular heat survival, the respective fusion proteins were validated by exposing PA-containing populations (MG1655 Δ*lacY* pTrc99A-*mCherry-cI78*^*EP8*^ cells, with the respective fusions) to a heat treatment and comparing inactivation levels to that of PA-containing populations of unlabeled cells (without any additional fluorescent fusions). For this, cells were first grown to exponential phase (OD600 = 0.2–0.3) in AB medium with 0.2% glycerol. PA production was subsequently induced by adding 1 mM IPTG to the medium for 1.5 h, after which the cells were harvested and washed into AB medium with 0.2% glucose. After washing, cells were exposed to a heat shock (52 °C, 15 min), and inactivation was determined through spot-plating experiments. In these experiments, the appropriate dilutions of a sample were prepared in 0.85% KCl and subsequently spot-plated (5 μl) on AB glucose agar. After 24 h of incubation at 37 °C, the number of survivors was scored, compared to that of untreated controls, and total inactivation was determined.

We further validated the transcriptional and translational fusions by exposing them to a sublethal heat shock (47 °C, 15 min), conditions known to activate the heat shock regulon of which the investigated proteins are a part of [93]. We quantified promoter activity and protein concentrations for the tested chaperones and proteases before and after heat treatment. For the transcriptional fusions, average cellular GFP fluorescence (corresponding to promoter activity) was determined directly after heat treatment. For the translational fusions, average cellular GFP fluorescence (corresponding to protein concentration) was determined after 30 min of incubation at 37 °C after heat treatment (allowing additional time for fusion protein folding and maturation).

Closer examination of the translational DnaK-msfGFP reporter strain revealed that this fusion protein occasionally displayed small and transient foci in unstressed PA-free cells (Fig 9B). Whether these foci reflect native, functionally relevant DnaK behavior or are artefactual (label-induced, but without severely compromising DnaK functioning) remains to be established.

### Bootstrapped estimation of the standard error of the mean fraction of surviving cells

To assess the variability in surviving cellular fractions, the original sample size was bootstrapped (sampled with replacement) 3,000 times, and the mean fraction of surviving cells was calculated for each of these samples. The bootstrapped estimation of the standard error of the mean fraction of surviving cells was subsequently calculated by taking the standard deviation of the bootstrapped means.

## Supporting information

S1 Fig Label-induced mislocalization of IbpA in stationary phase and IbpB in exponential and stationary phase *E*. *coli* cells. (A) Representative phase contrast, epifluorescence (reporting IbpA expression/production and localization), and superimposed images of *E*. *coli* MG1655 cells containing the indicated IbpA fluorescent fusion proteins. Scale bars correspond to 2 μm. (B) Measured distribution of punctate and diffuse fluorescence intensity for the indicated fusion proteins. The means of 3 independent experiments are shown, with error bars representing the standard deviation between experiments. The fluorescence intensity distribution of 30 individual cells was determined per experiment. (C) The average number of observed foci per cell for the indicated fusion proteins. The means of 3 independent experiments are shown, with error bars representing the standard deviation between experiments. Per experiment, at least 100 cells were examined to determine the average number of cellular foci. (D) Representative phase contrast, epifluorescence (reporting IbpB expression/production and localization), and superimposed images of *E*. *coli* MG1655 cells containing the indicated IbpB fluorescent fusion proteins. Scale bars correspond to 2 μm. (E) Calculated distribution of punctate and diffuse fluorescence intensity for the indicated fusion proteins. The means of 3 independent experiments are shown, with error bars representing the standard deviation between experiments. The fluorescence intensity distribution of 15 individual cells was determined per experiment. (F) The average number of observed foci per cell for the indicated fusion proteins. The means of 3 independent experiments are shown, with error bars representing the standard deviation between experiments. Per experiment, at least 72 cells were examined to determine the average number of cellular foci. The numerical data underlying this figure can be found in S2 Data. IbpA, inclusion body binding protein A; IbpB, inclusion body binding protein B.
(TIF)

S2 Fig Temperature-induced changes in IbpA expression and localization in *E*. *coli* MG1655 *ibpA-msfgfp* cells and bacterial aging in growing, PA-containing microcolonies. (A-B) Phase contrast, GFP epifluorescence (reporting IbpA expression/production and localization), and superimposed images of the same (A) control MG1655 *ibpA-msfgfp* cells before and (B) directly after exposure to a sublethal heat shock (47 °C, 15 min). Scale bars correspond to 2 μm. (C-D) Representative phase contrast, GFP epifluorescence (reporting IbpA expression/production and localization), and superimposed images of (C) control and (D) streptomycin-exposed (10 μg/ml, 1 h) MG1655 *ibpA-msfgfp* cells. Scale bars correspond to 2 μm. Green arrows indicate visible inclusion bodies. (E) Histograms showing the distribution of the average cellular GFP fluorescence of control and streptomycin-treated (10 μg/ml, 1 h) cells, derived from 3 independent experiments (*n* ≥ 61 cells per independent experiment). (F-H) Representative phase contrast, GFP epifluorescence, and images of MG1655 *ibpA-msfgfp* cells equipped with (F) pTVP1LAC, (G) pTVP1RFP, and (H) pMAL LRRK2. For each of the expression constructs, expression was induced by the addition of 1 mM IPTG. pTVP1LAC produces an engineered *E*. *coli* β-galactosidase fused to the aggregation-prone FMDV VP1 capsid protein [94]. pTVP1RFP is a similar construct, in which the β-galactosidase is replaced by an RFP [94,95]. Consequently, an extra panel displaying inclusion body–localized RFP fluorescence is also shown. pMAL LRRK2, on the other hand, produces large quantities of the human LRRK2, the protein that represents the most common monogenetic cause of Parkinson disease [96]. Scale bars correspond to 2 μm. Green arrows indicate visible inclusion bodies. (I) Histograms showing the distribution of the average cellular GFP fluorescence of control MG1655 *ibpA-msfgfp* cells and MG1655 *ibpA-msfgfp* cells expressing the various aggregating proteins. The distributions of average cellular fluorescence of cells derived from 3 independent experiments per strain are shown (*n* ≥ 60 per independent experiment). (J-K) The effect of bacterial aging on the fitness of proliferating fourth- and fifth-generation cells was examined by comparing the growth rate of the oldest cells, defined as those inheriting the oldest cell poles [17], to that of the remainder of the population. (J) Violin plots comparing the distribution of growth rates of the oldest MG1655 *ibpA-msfgfp* cells to that of the remainder of the population (*n*_microlonies_ = 10, *n*_cells_ = 478) during growth after application of a sublethal heat shock (47 °C, 15 min). Asterisk indicates that a significant difference could be detected between the average growth rate of both classes (Student *t* test, *p*-value = 6.82 × 10^−8^). A similar fitness defect (9.36%, Student *t* test, *p*-value = 8.96 × 10^−7^) could be detected when only PA-free cells were considered. (K) Similar plot for MG1655 Δ*lacY* pTrc99A-*mCer-cI78*^*EP8*^ cells (*n*_microlonies_ = 8, *n*_cells_ = 384) during growth after expression of the aggregating protein was halted. Asterisk indicates that a significant difference could be detected between the average growth rate of both classes (Student *t* test, *p*-value = 2.73 × 10^−4^). The numerical data underlying this figure can be found in S2 Data. FMDV, foot-and-mouth disease virus; GFP, green fluorescent protein; IbpA, inclusion body binding protein A; LRRK2, leucine-rich repeat kinase 2; RFP, red fluorescent protein.
(TIF)

S3 Fig Asymmetrically inherited IbpA-msfGFP foci represent natively occurring structures. (A-B) Representative phase contrast and GFP epifluorescence (reporting IbpA expression/production and localization) images of a TLFM microscopy image sequence of growing (A) MG1655 pBAD33-*ibpA-msfgfp* cells after exposure to a sublethal heat shock (47 °C, 15 min) or (B) unstressed control cells in the presence of 0.15% L-arabinose. Before TLFM, cells were grown to exponential phase in LB medium supplemented with 0.2% glucose to repress expression of the fusion protein. Scale bars correspond to 5 μm. (C) Representative phase contrast and GFP epifluorescence images illustrating the typical microcolonies emerging from unstressed MG1655 pBAD33-*ibpA-msfgfp* control cells (upper panels) and MG1655 pBAD33-*ibpA-msfgfp* cells exposed to a sublethal heat treatment (47 °C, 15 min; lower panels), after subsequent growth in LB supplemented with the indicated amount of L-arabinose for 100 min. Scale bar corresponds to 5 μm. GFP, green fluorescent protein; IbpA, inclusion body binding protein A; LB, lysogeny broth; msfGFP, monomeric superfolder GFP; TLFM, time-lapse fluorescence microscopy.
(TIF)

S4 Fig The IbpA-msfGFP concentration gradient is not a consequence of transcriptional *ibpA* up-regulation in PA-bearing cells. (A) Correlation between *ibpA* promoter activity (as measured by average cellular mCherry fluorescence) and IbpA concentration (as measured by average cellular GFP fluorescence) for individual MG1655 *ibpA-msfgfp* pSG1 cells (Pearson’s r = 0.5325, *p*-value = 3.96 × 10^−22^) in unstressed control populations (*n* = 291 cells). (B) Representative phase contrast, mCherry epifluorescence (reporting *ibpA* promoter activity), and GFP epifluorescence (reporting IbpA concentration and localization) images of a TLFM image sequence of MG1655 *ibpA-msfgfp* pSG1 cells after exposure to a sublethal heat shock (47 °C, 15 min). Scale bar corresponds to 2 μm. (C) Correlation between *ibpA* promoter activity (as measured by average cellular mCherry fluorescence) and IbpA concentration (as measured by average cellular GFP fluorescence) for individual MG1655 *ibpA-msfgfp* pSG1 cells (*n* = 361 cells, Pearson’s r = 0.1025, *p*-value = 5.96 × 10^−2^) 130 min after exposure to a sublethal heat shock (47 °C, 15 min). Although for most cells, as expected, a relatively good correlation can be observed, PA-bearing cells (as indicated by cells having a significantly above average cellular GFP fluorescence) do not display a significantly increased *ibpA* expression level (as indicated by their not significantly increased average cellular mCherry fluorescence). The latter leads to the observed insignificant correlation, on average, between the two variables. The numerical data underlying this figure can be found in S2 Data. GFP, green fluorescent protein; IbpA, inclusion body binding protein A; msfGFP, monomeric superfolder GFP; PA, protein aggregate; TLFM, time-lapse fluorescence microscopy.
(TIF)

S5 Fig Asymmetric segregation of PAs raised by exposure to a sublethal streptomycin or hydrogen peroxide treatment. (A-B) Representative phase contrast, GFP epifluorescence (reporting IbpA concentration and localization), and superimposed images of a TLFM image sequence of an MG1655 *ibpA-msfgfp* cell growing into microcolonies after exposure to a sublethal (A) streptomycin treatment (15 μg/ml, 30 min) or (B) H_2_O_2_ treatment (6 mM, 90 min). Scale bars correspond to 2 μm. GFP, green fluorescent protein; PA, protein aggregate; TLFM, time-lapse fluorescence microscopy.
(TIF)

S6 Fig Cell age does not affect survival frequency. For PA-containing microcolonies, the fraction of cells surviving the second heat shock (51 °C, 7 min) is binned by cell age for (A) all cells and (B) all PA-free cells. The dotted line indicates average survival of all cells included in the analysis; no ages that significantly differ in survival frequency could be detected (Fisher’s exact test). Numbers in white indicate the number of cells included in each bin. Error bars indicate bootstrapped estimates of the standard error of the mean fraction of surviving cells. The numerical data underlying this figure can be found in S2 Data. PA, protein aggregate.
(TIF)

S7 Fig Validation of the new synthetic PA model system in *E*. *coli*. (A) Representative phase contrast, CFP epifluorescence (reporting mCer-cI78^EP8^ localization), YFP epifluorescence (reporting HupA-Venus, and thus nucleoid localization), and superimposed images of MG1655 Δ*lacY hupA-Venus* pTrc99A-*mCer-cI78*^*EP8*^ cells. (B-C) Representative images of a TLFM image sequence of (B) a nucleoid-containing and (C) an anucleate MG1655 Δ*lacY* Δ*recA* pTrc99A-*mCer-cI78*^*EP8*^ PA-bearing cell. DAPI epifluorescence images (reporting the nucleoid) and phase contrast images superimposed with CFP epifluorescence images (reporting mCer-cI78^EP8^ behavior) are shown at the indicated times after beginning of time-lapse recording. Please note that CFP epifluorescence bleeds through in the DAPI channel and that, as a consequence, the distinct foci observed in the latter channel thus correspond to mCer-cI78^EP8^ foci and do not contain any DAPI-labeled DNA, nor are they associated with the nucleoid. (D) Cumulative histograms showing the distribution of average cellular mCherry fluorescence of MG1655 Δ*lacY* pTrc99A-*mCer-cI78*^*WT*^ pSG1 and MG1655 Δ*lacY* pTrc99A-*mCer-cI78*^*EP8*^ pSG1 cells under inducing conditions (1 mM IPTG). The combined cellular mCherry fluorescence distribution of 3 independent experiments is shown (*n* ≥ 77 cells per independent experiment). A K-S test (*p*-value = 2.93 × 10^−22^) indicated that expression was significantly increased in cells expressing the EP8 variant. (E-F) Phase contrast, GFP epifluorescence (reporting IbpA concentration and localization), mCherry epifluorescence, and phase contrast images superimposed with GFP and mCherry epifluorescence images of MG1655 *ibpA-msfgfp* pTrc99A-*mCherry-cI78*^*EP8*^ cells showing the colocalization of IbpA-msfGFP and mCherry-cI78^EP8^ foci. All scale bars correspond to 1 μm. (G) Relative fitness of the MG1655 Δ*lacY* pTrc99A-*mCer-cI78*^*EP8*^ strain as compared to the MG1655 Δ*lacY* pTrc99A-*mCer-cI78*^*WT*^ strain under different induction regimes (from left to right: no IPTG, 10, 25, 50, 100, 200, 500, and 1,000 μM IPTG). The means of 3 independent experiments are shown, with error bars representing the standard deviation between experiments. Per experiment, the growth rates of at least 25 microcolonies were examined. (H) Correlation between microcolony growth rate and average microcolony fluorescence in fully induced conditions (1 mM IPTG) for MG1655 Δ*lacY* pTrc99A-*mCer-cI78*^*EP8*^ microcolonies (1 mM IPTG, Spearman’s ranked correlation coefficient ρ = −0.6678, *p*-value = 4.73 × 10^−18^, *n* = 133 cells). (I) Correlation between microcolony growth rate and average microcolony fluorescence in fully induced conditions (1 mM IPTG) for MG1655 Δ*lacY* pTrc99A-*mCer-cI78*^*WT*^ microcolonies (1 mM IPTG, ρ = 0.2176, *p*-value = 1.54 × 10^−2^, *n* = 135 cells). The numerical data underlying this figure can be found in S2 Data. CFP, cyan fluorescent protein; DAPI, 4′,6-diamidino-2-phenylindole; GFP, green fluorescent protein; IbpA, inclusion body binding protein A; IPTG, isopropyl β-D-1-thiogalactopyranoside; K-S, Kolmogorov-Smirnov; mCer, monomeric cerulean; PA, protein aggregate; TLFM, time-lapse fluorescence microscopy; YFP, yellow fluorescent protein.
(TIF)

S8 Fig Correlation between PA size and total cellular fluorescence. Correlation between PA size (determined directly using the objectDetection module within the Oufti software [92]) and total cellular fluorescence (r = 0.7551, *p*-value = 1.66 × 10^−65^) for the MG1655 Δ*lacY* pTrc99A-*mCer-cI78*^*EP8*^ cells (*n* = 352 cells) also used in Fig 7E. The numerical data underlying this figure can be found in S2 Data. PA, protein aggregate.
(TIF)

S9 Fig Ongoing PA production sensitizes cells toward heat stress. Fraction of surviving MG1655 Δ*lacY* pTrc99A-*mCer-cI78*^*WT*^ and MG1655 Δ*lacY* pTrc99A-*mCer-cI78*^*EP8*^ cells after application of a semilethal heat shock (49 °C, 15 min) during induction of PA production (1 mM IPTG). The means of 9 independent experiments are shown with error bars representing the standard error of the mean. A significant difference in survival frequency could be detected (Student *t* test, *p*-value = 3.10 × 10^−3^). The numerical data underlying this figure can be found in S2 Data. IPTG, isopropyl β-D-1-thiogalactopyranoside; PA, protein aggregate.
(TIF)

S10 Fig Investigation of the potential role of the *ibp* operon in establishing the PA-mediated asymmetry in robustness through the use of deletion mutants. Survival probability of PA-free and PA-bearing MG1655 Δ*lacY* pTrc99A-*mCer-cI78*^*EP8*^ cells (designated as WT) and their indicated deletion mutants upon exposure to a heat shock (52 °C, 7 min). Similar experimental setup as in Fig 6E. Asterisks indicate a significant difference in survival frequency between both cellular classes (Student *t* test, *p*-values = 2.83 × 10^−7^, 1.90 × 10^−2^, 1.28 × 10^−4^, and 1.09 × 10^−5^). Numbers in black indicate the number of cells included in each bin. ND = no surviving cells could be detected within the 8 h time frame after heat shock. Error bars indicate bootstrapped estimates of the standard error of the mean fraction of surviving cells. The numerical data underlying this figure can be found in S2 Data. PA, protein aggregate; WT, wild type.
(TIF)

S11 Fig Validation of transcriptional and translational msfGFP fusions of different protein quality control components. (A-B) PA-containing populations of MG1655 Δ*lacY* pTrc99A-*mCherry-cI78*^*EP8*^ (designated as WT) and the indicated (A) transcriptional and (B) translational fusion strains were exposed to a heat treatment (52 °C, 15 min), after which cellular inactivation was determined. Asterisk indicates a significant difference in inactivation of the *clpP* transcriptional fusion strain in comparison to its unlabeled control (Student *t* test, *p*-values = 1.84 × 10^−3^). The means of 3 independent experiments are shown, with error bars representing the standard deviation between experiments. (C) Quantification of the promoter activity of indicated protein quality control components (as measured by average cellular GFP fluorescence) in cells before and directly after a sublethal heat shock (47 °C, 15 min). Asterisks indicate a significant increase in average cellular GFP concentration in heat-shocked cells (directional Student *t* test, respective *p*-values = 1.55 × 10^−58^, 3.30 × 10^−10^, 1.56 × 10^−16^, 9.99 × 10^−1^, 2.66 × 10^−11^, 4.90 × 10^−9^, 2.96 × 10^−8^, and 3.11 × 10^−42^). Numbers in black indicate the number of cells included in each group. Error bars indicate the standard deviation. (D) Quantification of the concentration of indicated protein quality control components (as measured by average cellular GFP fluorescence) in cells before and 30 min after a sublethal heat shock (47 °C, 15 min). Asterisks indicate a significant increase in average cellular GFP concentration in heat-shocked cells (directional Student *t* test, respective *p*-values = 1.73 × 10^−37^, 9.99 × 10^−1^, 1.28 × 10^−30^, 9.99 × 10^−1^, 3.46 × 10^−8^, 9.38 × 10^−1^, 1.04 × 10^−1^, and 9.98 × 10^−1^). Numbers in black indicate the number of cells included in each group. Error bars indicate the standard deviation. The numerical data underlying this figure can be found in S2 Data. GFP, green fluorescent protein; msfGFP, monomeric superfolder GFP; PA, protein aggregate; WT, wild type.
(TIF)

S12 Fig No transcriptional response of protein quality control components to the presence of asymmetrically segregating PAs. (A) Quantification of the promoter activity of indicated protein quality control components (as measured by average cellular GFP fluorescence) in PA-free and PA-bearing MG1655 Δ*lacY* pTrc99A-*mCherry-cI78*^*EP8*^ cells, 3 h after PA production was halted. No significant average up-regulation could be detected in PA-bearing cells (directional Student *t* test, *p*-value = 1 for all transcriptional fusions). Numbers in black indicate the number of cells included in each group. Error bars indicate the standard deviation. (B) Representative GFP epifluorescence (reporting expression level of the indicated gene), mCherry epifluorescence (reporting mCherry-cI78^EP8^ localization), and superimposed images of the indicated transcriptional fusion strains, 3 h after PA production was halted. Scale bar corresponds to 5 μm. (C) Zoomed-in images of the MG1655 Δ*lacY* P_*htpG*_*-msfgfp* pTrc99A-*mCherry-cI78*^*EP8*^ strain illustrating the impermeability of PAs to cytosolic GFP (indicated by the white arrows in the GFP epifluorescence image). Scale bar corresponds to 5 μm. *Please note that the strain carrying the transcriptional *clpP* fusion is likely compromised, and its expression level could thus reflect nonnative behavior. The numerical data underlying this figure can be found in S2 Data. GFP, green fluorescent protein; PA, protein aggregate.
(TIF)

S13 Fig Localization and concentration of ClpX, Lon, HslU, and HtpG in the presence of asymmetrically segregating PAs. Representative GFP epifluorescence (reporting localization and concentration of the indicated translational fusion protein), mCherry epifluorescence (reporting mCherry-cI78^EP8^ localization), and superimposed images of the indicated translational fusion strains, 3 h after PA production was halted. Scale bar corresponds to 5 μm. The numerical data underlying this figure can be found in S2 Data. GFP, green fluorescent protein; PA, protein aggregate.
(TIF)

S14 Fig Synthetic PA production occasionally leads to the formation of nongrowing and likely anucleate cells. (A-B) Representative phase contrast, CFP epifluorescence (reporting mCer-cI78^EP8^ localization), and superimposed images of unstressed MG1655 Δ*lacY* pTrc99A-*mCer-cI78*^*EP8*^ cells after 1.5 h of induction with 1 mM IPTG. While (A) most cells are capable of growth, (B) some smaller, likely anucleate, cells are not seen to initiate or resume growth, even in control conditions in the absence of stress. All images are displayed on the same scale; scale bars correspond to 2 μm. The numerical data underlying this figure can be found in S2 Data. CFP, cyan fluorescent protein; IPTG, isopropyl β-D-1-thiogalactopyranoside; mCer, monomeric cerulean; PA, protein aggregate.
(TIF)

S1 Movie Asymmetric segregation of native PAs raised by exposure to sublethal heat shock. (Left) Phase contrast and (Right) GFP epifluorescence (reporting IbpA expression/production and localization) images in combination with cell outlines showing the growth of an individual MG1655 *ibpA-msfgfp* cell into a microcolony (with 3 PAs) after exposure to a sublethal heat shock (47 °C, 15 min). Time after heat shock is indicated in the top right corner. GFP, green fluorescent protein; IbpA, inclusion body binding protein A; PA, protein aggregate.
(AVI)

S2 Movie Asymmetric segregation of a native PA raised by exposure to sublethal heat shock. (Left) Phase contrast and (Right) GFP epifluorescence (reporting IbpA expression/production and localization) images in combination with cell outlines showing the growth of an individual MG1655 *ibpA-msfgfp* cell into a microcolony (with a single PA) after exposure to a sublethal heat shock (47 °C, 15 min). Time after heat shock is indicated in the top right corner. GFP, green fluorescent protein; IbpA, inclusion body binding protein A; PA, protein aggregate.
(AVI)

S3 Movie Asymmetric segregation of a PA raised by induction of an aggregating protein (mCer-cI78^EP8^). (Left) Phase contrast and (Right) CFP epifluorescence (reporting mCer-cI78^EP8^ localization) images in combination with cell outlines showing the growth of an individual MG1655 Δ*lacY* pTrc99A-*mCer-cI78*^*EP8*^ cell into a microcolony after induction of the aggregation-prone and PA-forming mCer-cI78^EP8^ was halted. Time after halting of induction is indicated in the top left corner. CFP, cyan fluorescent protein; mCer, monomeric cerulean; PA, protein aggregate.
(AVI)

S1 Data Excel file containing all numerical data used to generate the main figures. Each tab corresponds to a different figure.
(XLSX)

S2 Data Excel file containing all numerical data used to generate the supporting figures. Each tab corresponds to a different supporting figure.
(XLSX)

S1 Table Amino acid sequences of fluorescent proteins. (DOCX)

S2 Table Overview of strain used in this study. (DOCX)

S3 Table Overview of plasmids used in this study. (DOCX)

S4 Table Overview of primers used in this study. When relevant, primer attachment sites are indicated in bold, linker sequences in blue, spacer sequences in orange, artificial ribosome binding sites in purple, and restriction sites in red.
(DOCX)

## Abbreviations

A.U.: arbitrary unit
DAPI: 4′,6-diamidino-2-phenylindole
Hsp100: heat shock protein 100
IbpA: inclusion body binding protein A
IbpB: inclusion body binding protein B
IPTG: isopropyl β-D-1-thiogalactopyranoside
K-S: Kolmogorov-Smirnov
LB: lysogeny broth
mCer: monomeric cerulean
msfGFP: monomeric superfolder green fluorescent protein
PA: protein aggregate
P_*ibpA*_: *ibpA* promoter
ROS: reactive oxygen species
TLFM: time-lapse fluorescence microscopy
YFP: yellow fluorescent protein
