# Supplementary figures and images for: Protein aggregates encode epigenetic memory of stressful encounters in individual Escherichia coli cells
Source: PLoS Biol. 2018 Aug 28;16(8):e2003853. doi: 10.1371/journal.pbio.2003853 (PMC6112618; doi:10.1371/journal.pbio.2003853)

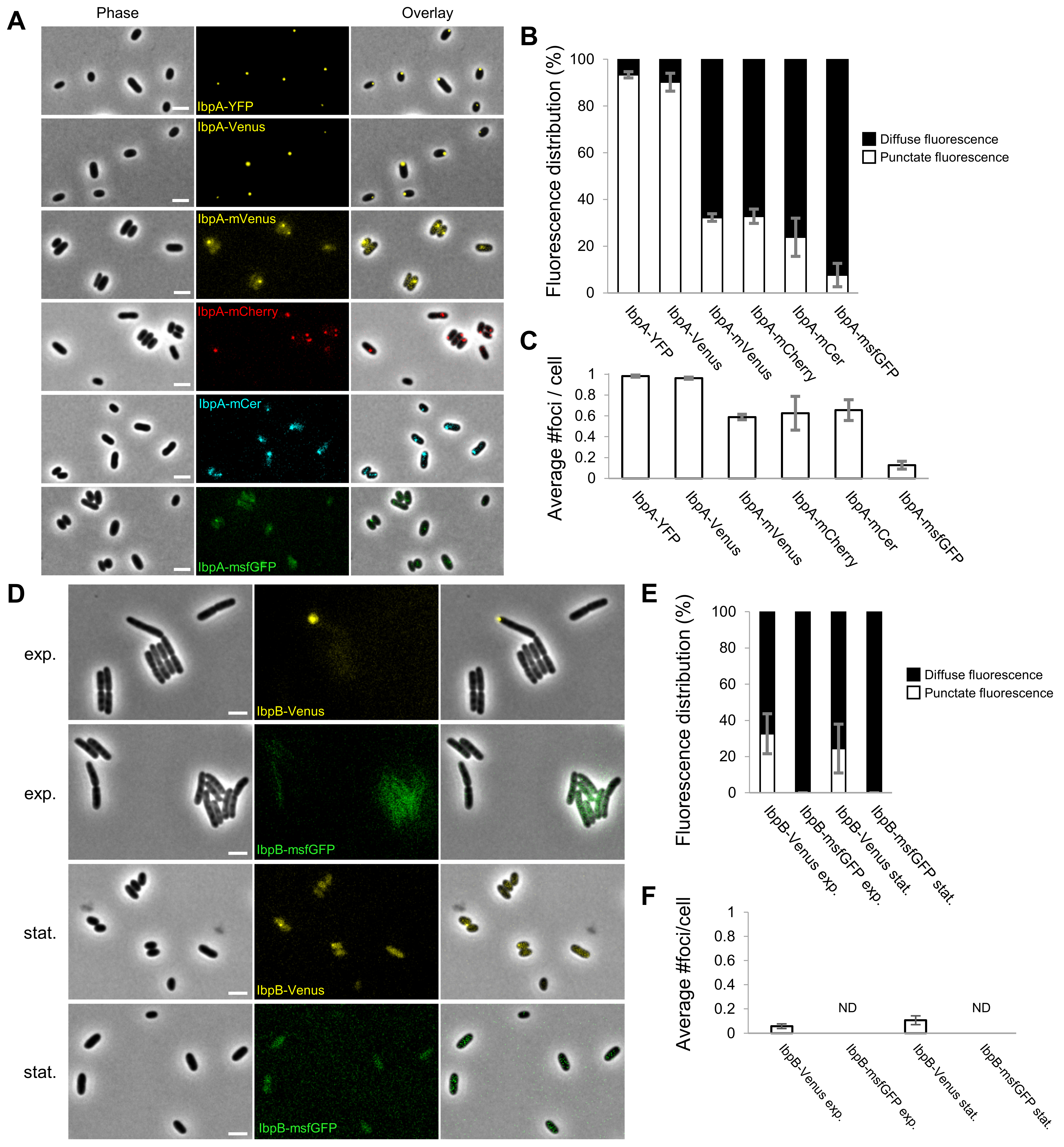

Supplement: S1 Fig — (A) Representative phase contrast, epifluorescence (reporting IbpA expression/production and localization), and superimposed images of E. coli MG1655 cells containing the indicated IbpA fluorescent fusion proteins. Scale bars correspond to 2 μm. (B) Measured distribution of punctate and diffuse fluorescence intensity for the indicated fusion proteins. The means of 3 independent experiments are shown, with error bars representing the standard deviation between experiments. The fluorescence intensity distribution of 30 individual cells was determined per experiment. (C) The average number of observed foci per cell for the indicated fusion proteins. The means of 3 independent experiments are shown, with error bars representing the standard deviation between experiments. Per experiment, at least 100 cells were examined to determine the average number of cellular foci. (D) Representative phase contrast, epifluorescence (reporting IbpB expression/production and localization), and superimposed images of E. coli MG1655 cells containing the indicated IbpB fluorescent fusion proteins. Scale bars correspond to 2 μm. (E) Calculated distribution of punctate and diffuse fluorescence intensity for the indicated fusion proteins. The means of 3 independent experiments are shown, with error bars representing the standard deviation between experiments. The fluorescence intensity distribution of 15 individual cells was determined per experiment. (F) The average number of observed foci per cell for the indicated fusion proteins. The means of 3 independent experiments are shown, with error bars representing the standard deviation between experiments. Per experiment, at least 72 cells were examined to determine the average number of cellular foci. The numerical data underlying this figure can be found in S2 Data. IbpA, inclusion body binding protein A; IbpB, inclusion body binding protein B. (TIF) [file pbio.2003853.s001.tif]

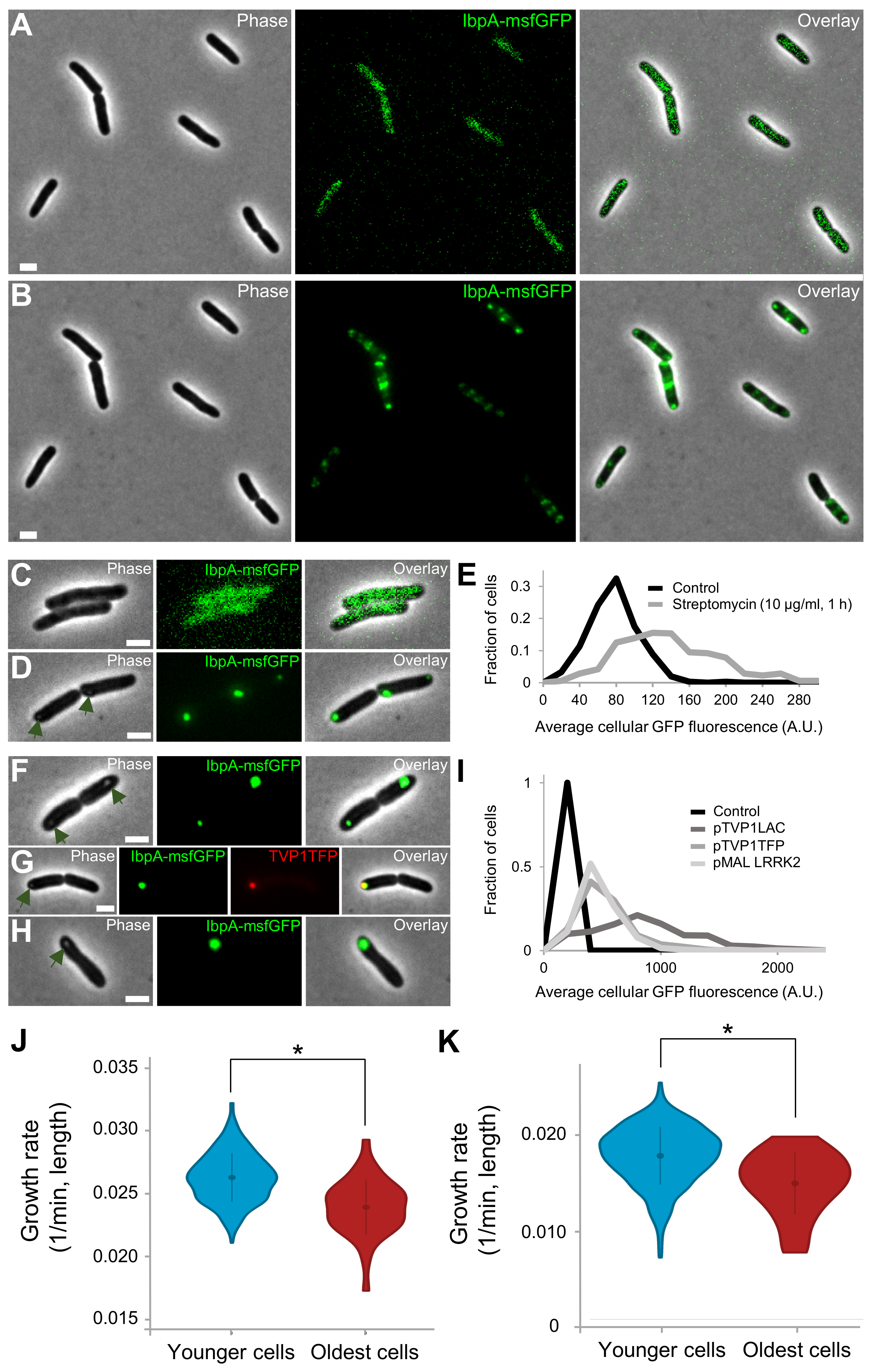

Supplement: S2 Fig — (A-B) Phase contrast, GFP epifluorescence (reporting IbpA expression/production and localization), and superimposed images of the same (A) control MG1655 ibpA-msfgfp cells before and (B) directly after exposure to a sublethal heat shock (47 °C, 15 min). Scale bars correspond to 2 μm. (C-D) Representative phase contrast, GFP epifluorescence (reporting IbpA expression/production and localization), and superimposed images of (C) control and (D) streptomycin-exposed (10 μg/ml, 1 h) MG1655 ibpA-msfgfp cells. Scale bars correspond to 2 μm. Green arrows indicate visible inclusion bodies. (E) Histograms showing the distribution of the average cellular GFP fluorescence of control and streptomycin-treated (10 μg/ml, 1 h) cells, derived from 3 independent experiments (n ≥ 61 cells per independent experiment). (F-H) Representative phase contrast, GFP epifluorescence, and images of MG1655 ibpA-msfgfp cells equipped with (F) pTVP1LAC, (G) pTVP1RFP, and (H) pMAL LRRK2. For each of the expression constructs, expression was induced by the addition of 1 mM IPTG. pTVP1LAC produces an engineered E. coli β-galactosidase fused to the aggregation-prone FMDV VP1 capsid protein [94]. pTVP1RFP is a similar construct, in which the β-galactosidase is replaced by an RFP [94,95]. Consequently, an extra panel displaying inclusion body–localized RFP fluorescence is also shown. pMAL LRRK2, on the other hand, produces large quantities of the human LRRK2, the protein that represents the most common monogenetic cause of Parkinson disease [96]. Scale bars correspond to 2 μm. Green arrows indicate visible inclusion bodies. (I) Histograms showing the distribution of the average cellular GFP fluorescence of control MG1655 ibpA-msfgfp cells and MG1655 ibpA-msfgfp cells expressing the various aggregating proteins. The distributions of average cellular fluorescence of cells derived from 3 independent experiments per strain are shown (n ≥ 60 per independent experiment). (J-K) The effect of bacterial aging on [file pbio.2003853.s002.tif]

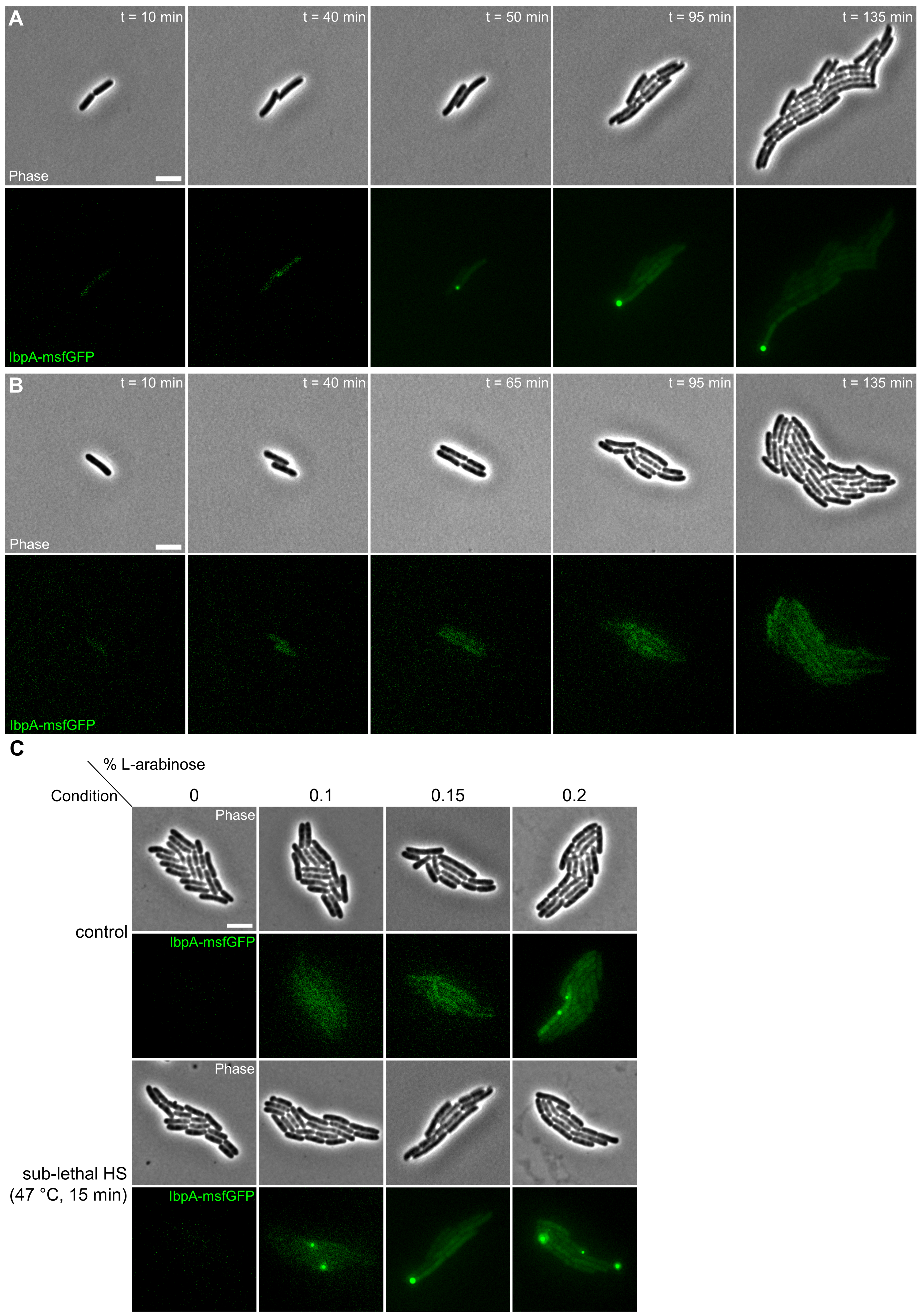

Supplement: S3 Fig — (A-B) Representative phase contrast and GFP epifluorescence (reporting IbpA expression/production and localization) images of a TLFM microscopy image sequence of growing (A) MG1655 pBAD33-ibpA-msfgfp cells after exposure to a sublethal heat shock (47 °C, 15 min) or (B) unstressed control cells in the presence of 0.15% L-arabinose. Before TLFM, cells were grown to exponential phase in LB medium supplemented with 0.2% glucose to repress expression of the fusion protein. Scale bars correspond to 5 μm. (C) Representative phase contrast and GFP epifluorescence images illustrating the typical microcolonies emerging from unstressed MG1655 pBAD33-ibpA-msfgfp control cells (upper panels) and MG1655 pBAD33-ibpA-msfgfp cells exposed to a sublethal heat treatment (47 °C, 15 min; lower panels), after subsequent growth in LB supplemented with the indicated amount of L-arabinose for 100 min. Scale bar corresponds to 5 μm. GFP, green fluorescent protein; IbpA, inclusion body binding protein A; LB, lysogeny broth; msfGFP, monomeric superfolder GFP; TLFM, time-lapse fluorescence microscopy. (TIF) [file pbio.2003853.s003.tif]

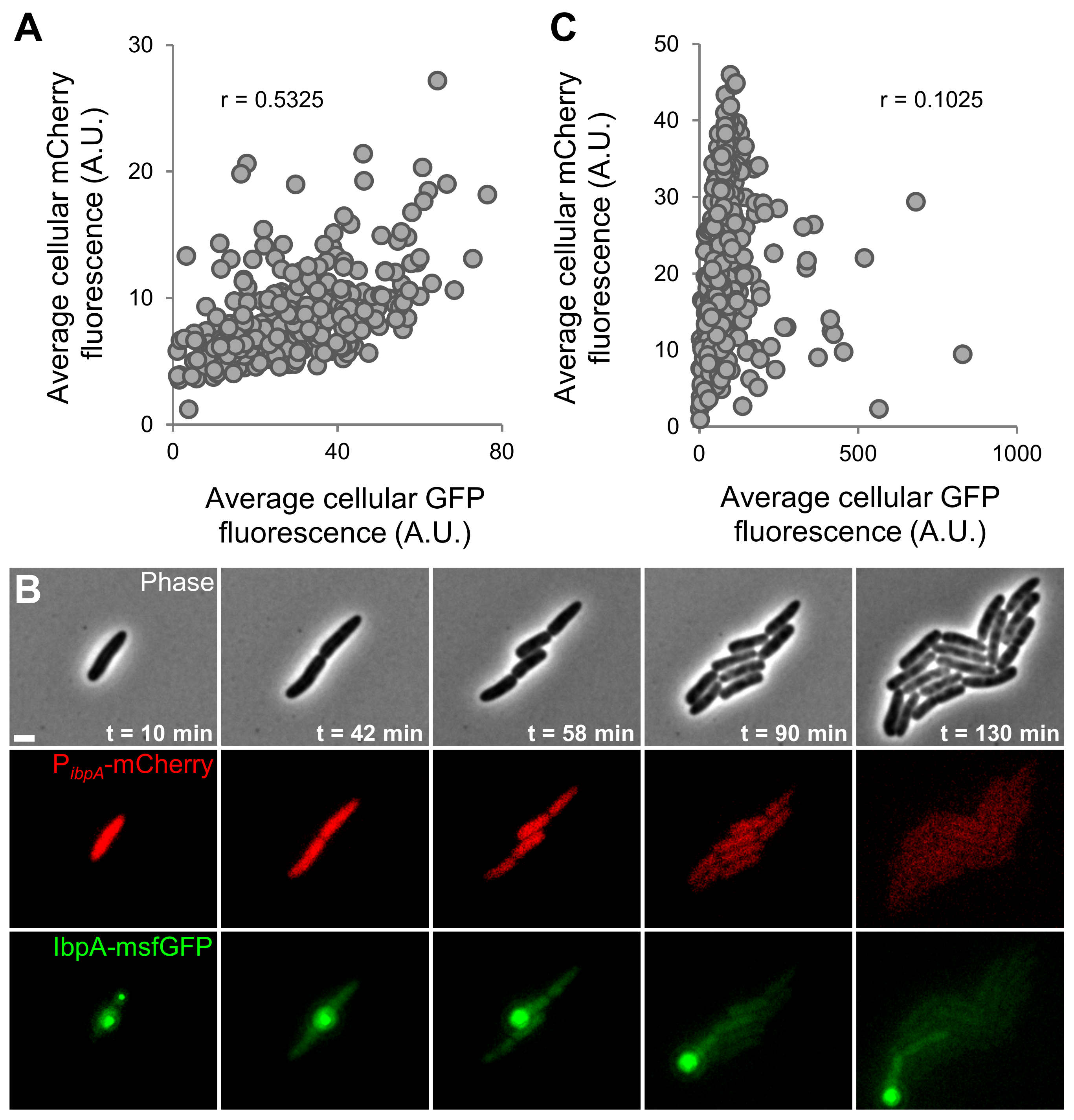

Supplement: S4 Fig — (A) Correlation between ibpA promoter activity (as measured by average cellular mCherry fluorescence) and IbpA concentration (as measured by average cellular GFP fluorescence) for individual MG1655 ibpA-msfgfp pSG1 cells (Pearson’s r = 0.5325, p-value = 3.96 × 10^−22^) in unstressed control populations (n = 291 cells). (B) Representative phase contrast, mCherry epifluorescence (reporting ibpA promoter activity), and GFP epifluorescence (reporting IbpA concentration and localization) images of a TLFM image sequence of MG1655 ibpA-msfgfp pSG1 cells after exposure to a sublethal heat shock (47 °C, 15 min). Scale bar corresponds to 2 μm. (C) Correlation between ibpA promoter activity (as measured by average cellular mCherry fluorescence) and IbpA concentration (as measured by average cellular GFP fluorescence) for individual MG1655 ibpA-msfgfp pSG1 cells (n = 361 cells, Pearson’s r = 0.1025, p-value = 5.96 × 10^−2^) 130 min after exposure to a sublethal heat shock (47 °C, 15 min). Although for most cells, as expected, a relatively good correlation can be observed, PA-bearing cells (as indicated by cells having a significantly above average cellular GFP fluorescence) do not display a significantly increased ibpA expression level (as indicated by their not significantly increased average cellular mCherry fluorescence). The latter leads to the observed insignificant correlation, on average, between the two variables. The numerical data underlying this figure can be found in S2 Data. GFP, green fluorescent protein; IbpA, inclusion body binding protein A; msfGFP, monomeric superfolder GFP; PA, protein aggregate; TLFM, time-lapse fluorescence microscopy. (TIF) [file pbio.2003853.s004.tif]

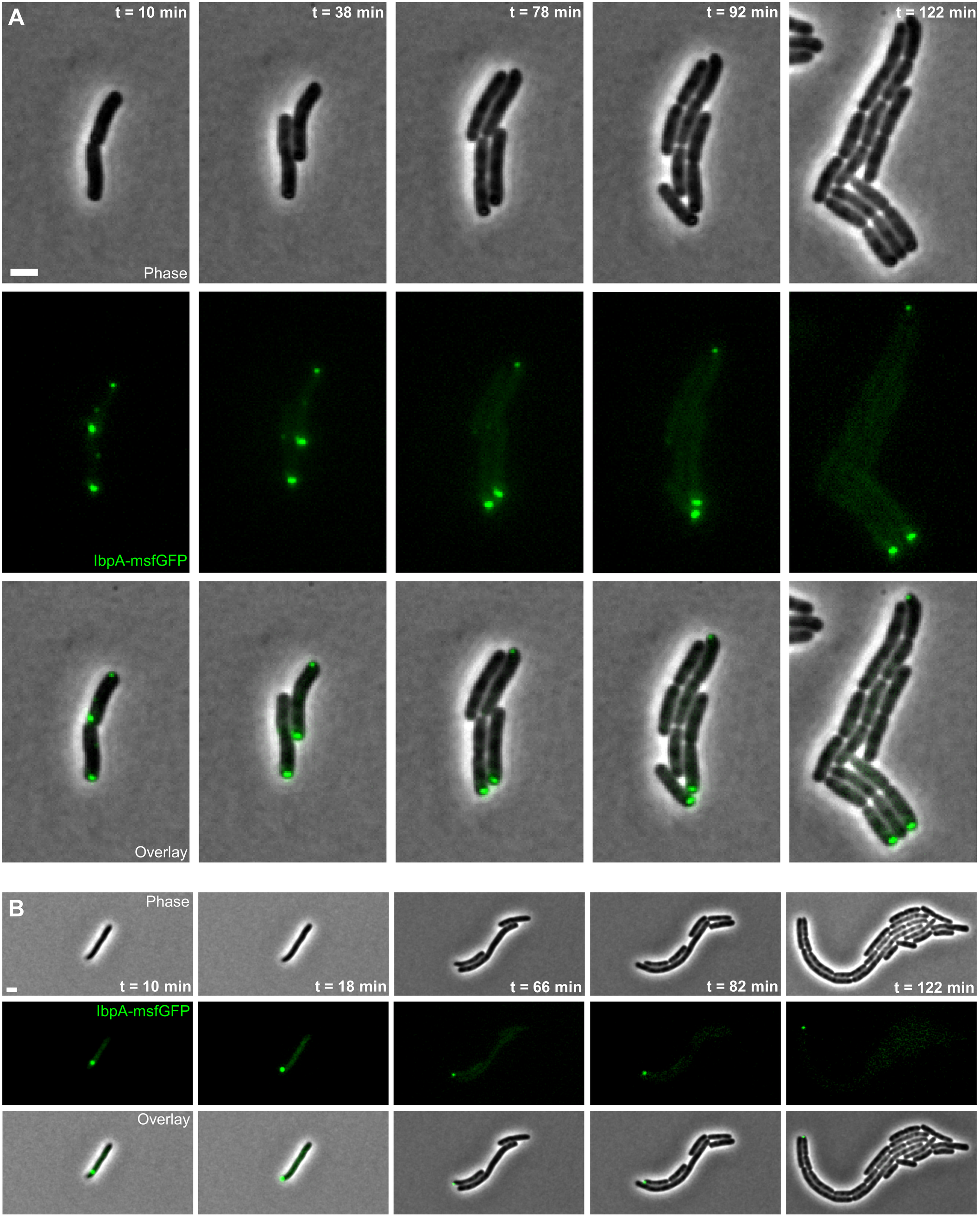

Supplement: S5 Fig — (A-B) Representative phase contrast, GFP epifluorescence (reporting IbpA concentration and localization), and superimposed images of a TLFM image sequence of an MG1655 ibpA-msfgfp cell growing into microcolonies after exposure to a sublethal (A) streptomycin treatment (15 μg/ml, 30 min) or (B) H2O2 treatment (6 mM, 90 min). Scale bars correspond to 2 μm. GFP, green fluorescent protein; PA, protein aggregate; TLFM, time-lapse fluorescence microscopy. (TIF) [file pbio.2003853.s005.tif]

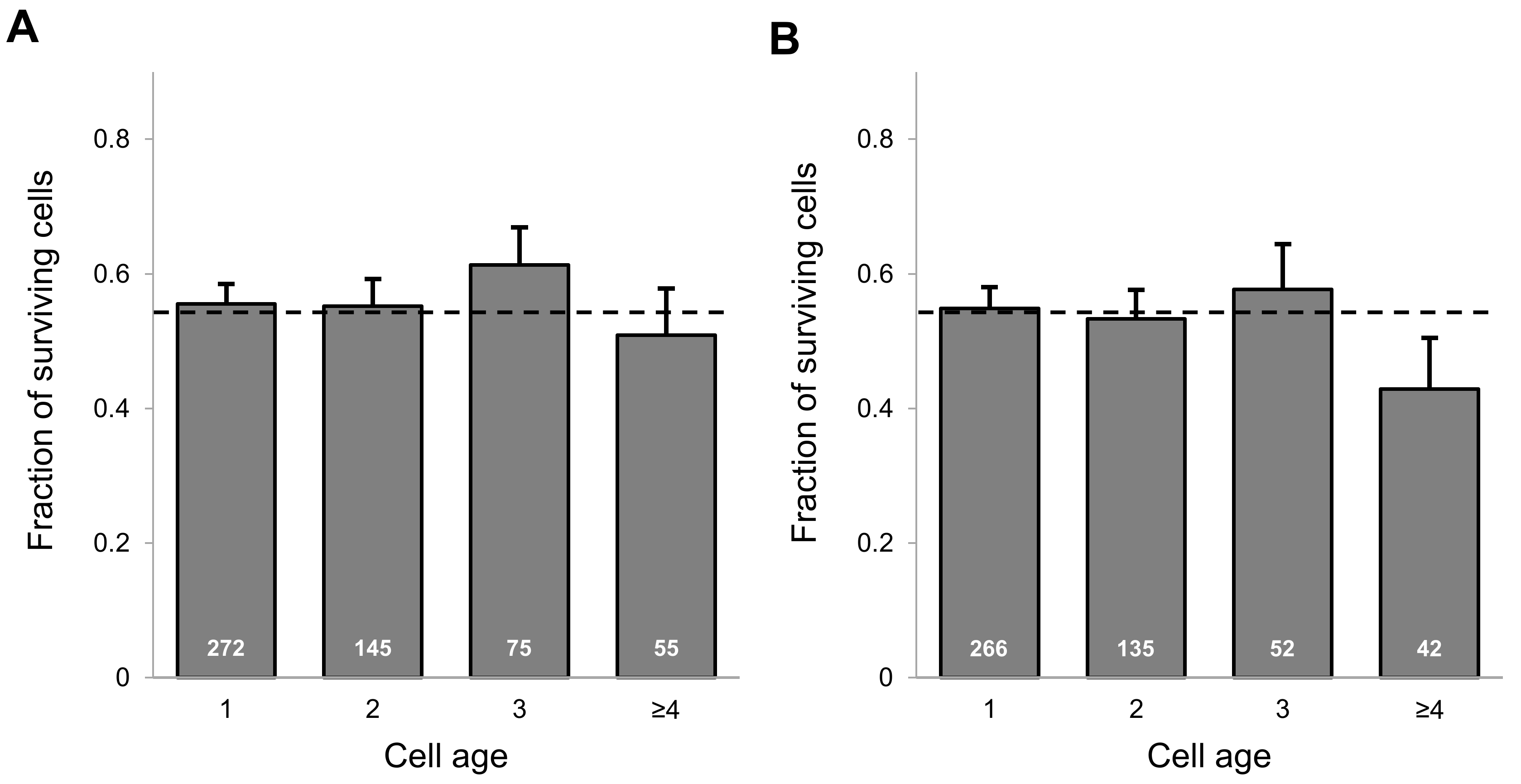

Supplement: S6 Fig — For PA-containing microcolonies, the fraction of cells surviving the second heat shock (51 °C, 7 min) is binned by cell age for (A) all cells and (B) all PA-free cells. The dotted line indicates average survival of all cells included in the analysis; no ages that significantly differ in survival frequency could be detected (Fisher’s exact test). Numbers in white indicate the number of cells included in each bin. Error bars indicate bootstrapped estimates of the standard error of the mean fraction of surviving cells. The numerical data underlying this figure can be found in S2 Data. PA, protein aggregate. (TIF) [file pbio.2003853.s006.tif]

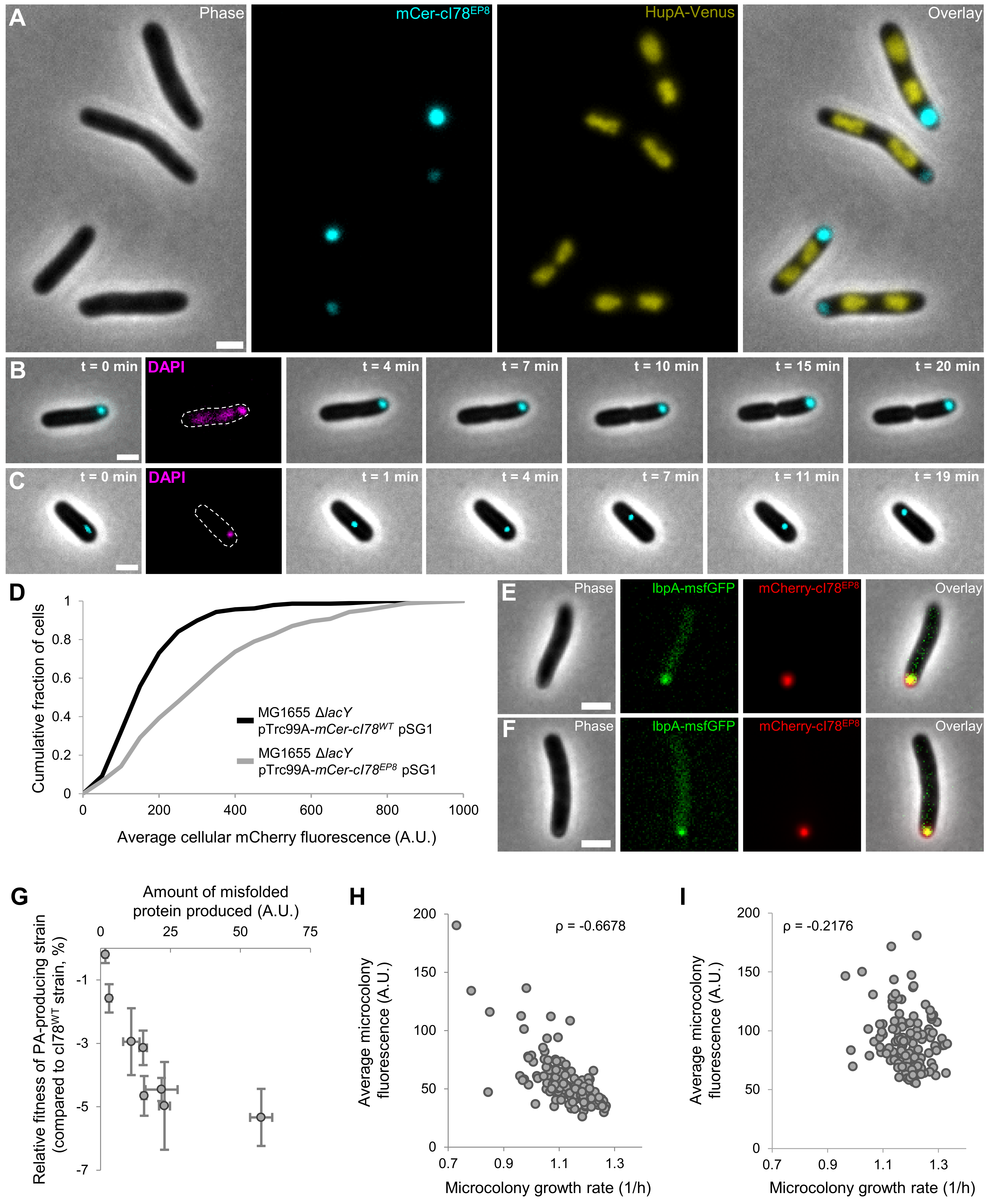

Supplement: S7 Fig — (A) Representative phase contrast, CFP epifluorescence (reporting mCer-cI78EP8 localization), YFP epifluorescence (reporting HupA-Venus, and thus nucleoid localization), and superimposed images of MG1655 ΔlacY hupA-Venus pTrc99A-mCer-cI78EP8 cells. (B-C) Representative images of a TLFM image sequence of (B) a nucleoid-containing and (C) an anucleate MG1655 ΔlacY ΔrecA pTrc99A-mCer-cI78EP8 PA-bearing cell. DAPI epifluorescence images (reporting the nucleoid) and phase contrast images superimposed with CFP epifluorescence images (reporting mCer-cI78EP8 behavior) are shown at the indicated times after beginning of time-lapse recording. Please note that CFP epifluorescence bleeds through in the DAPI channel and that, as a consequence, the distinct foci observed in the latter channel thus correspond to mCer-cI78EP8 foci and do not contain any DAPI-labeled DNA, nor are they associated with the nucleoid. (D) Cumulative histograms showing the distribution of average cellular mCherry fluorescence of MG1655 ΔlacY pTrc99A-mCer-cI78WT pSG1 and MG1655 ΔlacY pTrc99A-mCer-cI78EP8 pSG1 cells under inducing conditions (1 mM IPTG). The combined cellular mCherry fluorescence distribution of 3 independent experiments is shown (n ≥ 77 cells per independent experiment). A K-S test (p-value = 2.93 × 10^−22^) indicated that expression was significantly increased in cells expressing the EP8 variant. (E-F) Phase contrast, GFP epifluorescence (reporting IbpA concentration and localization), mCherry epifluorescence, and phase contrast images superimposed with GFP and mCherry epifluorescence images of MG1655 ibpA-msfgfp pTrc99A-mCherry-cI78EP8 cells showing the colocalization of IbpA-msfGFP and mCherry-cI78EP8 foci. All scale bars correspond to 1 μm. (G) Relative fitness of the MG1655 ΔlacY pTrc99A-mCer-cI78EP8 strain as compared to the MG1655 ΔlacY pTrc99A-mCer-cI78WT strain under different induction regimes (from left to right: no IPTG, 10, 25, 50, 100, 200, 500, and 1,000 μM IPTG). The means o [file pbio.2003853.s007.tif]

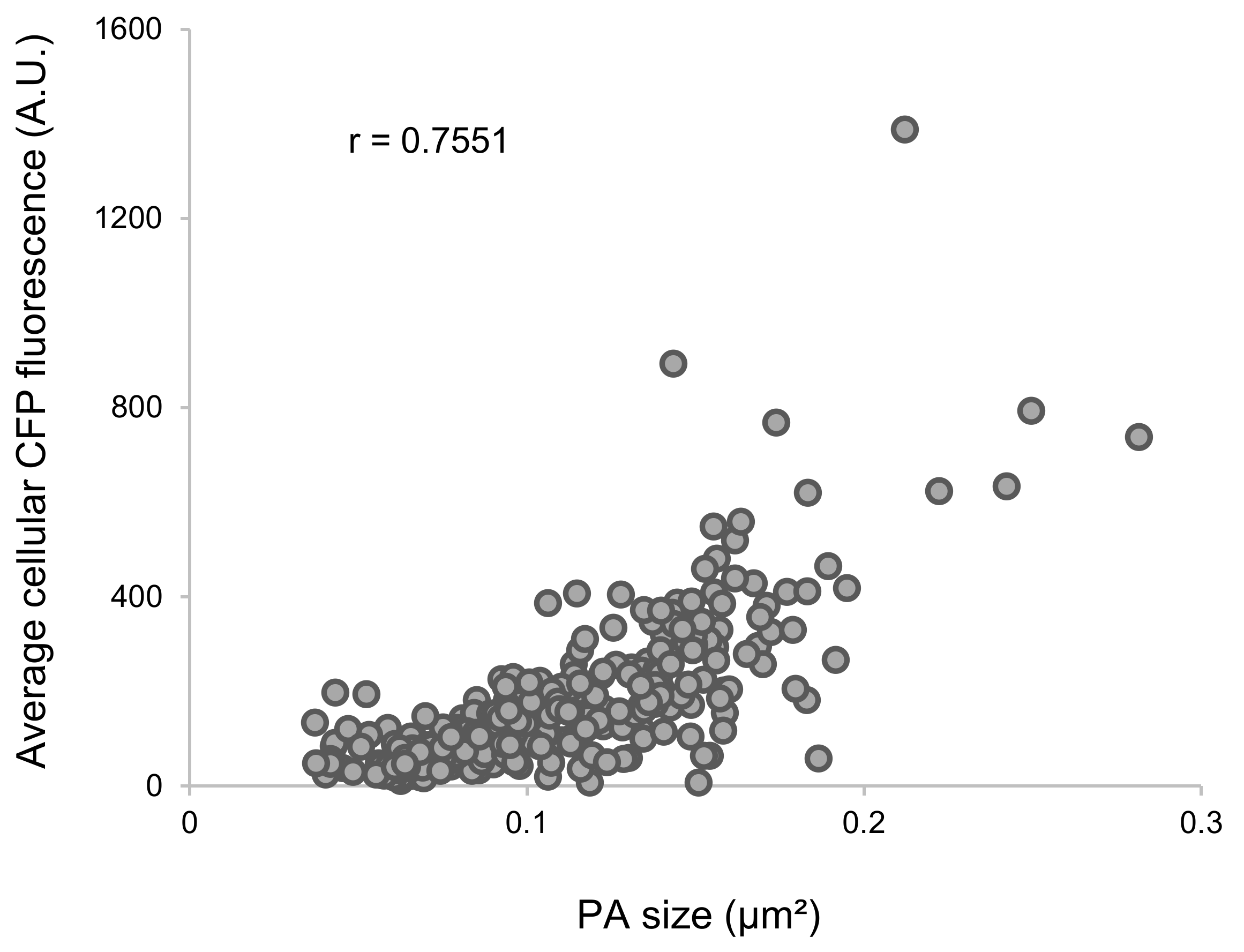

Supplement: S8 Fig — Correlation between PA size (determined directly using the objectDetection module within the Oufti software [92]) and total cellular fluorescence (r = 0.7551, p-value = 1.66 × 10^−65^) for the MG1655 ΔlacY pTrc99A-mCer-cI78EP8 cells (n = 352 cells) also used in Fig 7E. The numerical data underlying this figure can be found in S2 Data. PA, protein aggregate. (TIF) [file pbio.2003853.s008.tif]

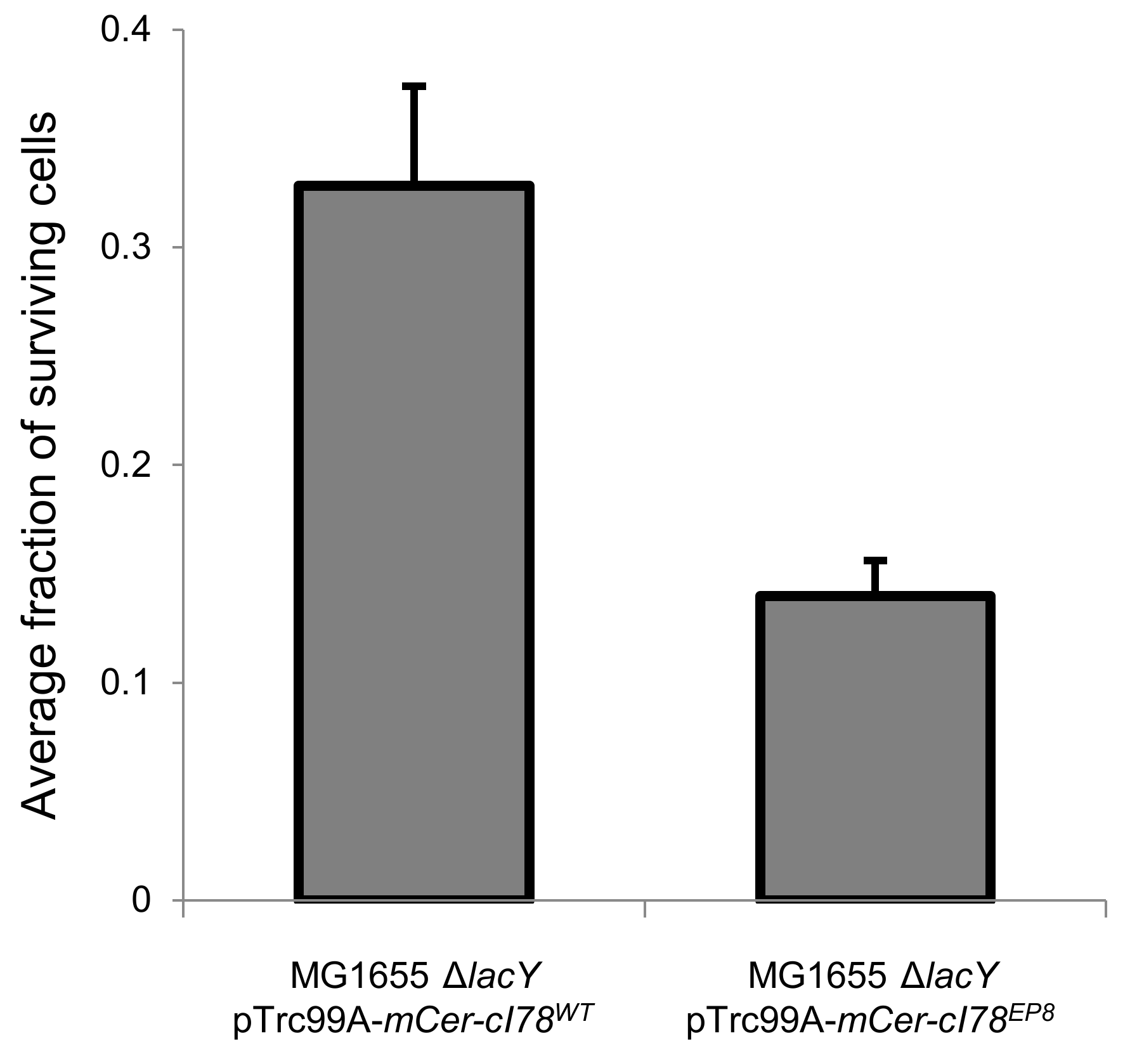

Supplement: S9 Fig — Fraction of surviving MG1655 ΔlacY pTrc99A-mCer-cI78WT and MG1655 ΔlacY pTrc99A-mCer-cI78EP8 cells after application of a semilethal heat shock (49 °C, 15 min) during induction of PA production (1 mM IPTG). The means of 9 independent experiments are shown with error bars representing the standard error of the mean. A significant difference in survival frequency could be detected (Student t test, p-value = 3.10 × 10^−3^). The numerical data underlying this figure can be found in S2 Data. IPTG, isopropyl β-D-1-thiogalactopyranoside; PA, protein aggregate. (TIF) [file pbio.2003853.s009.tif]

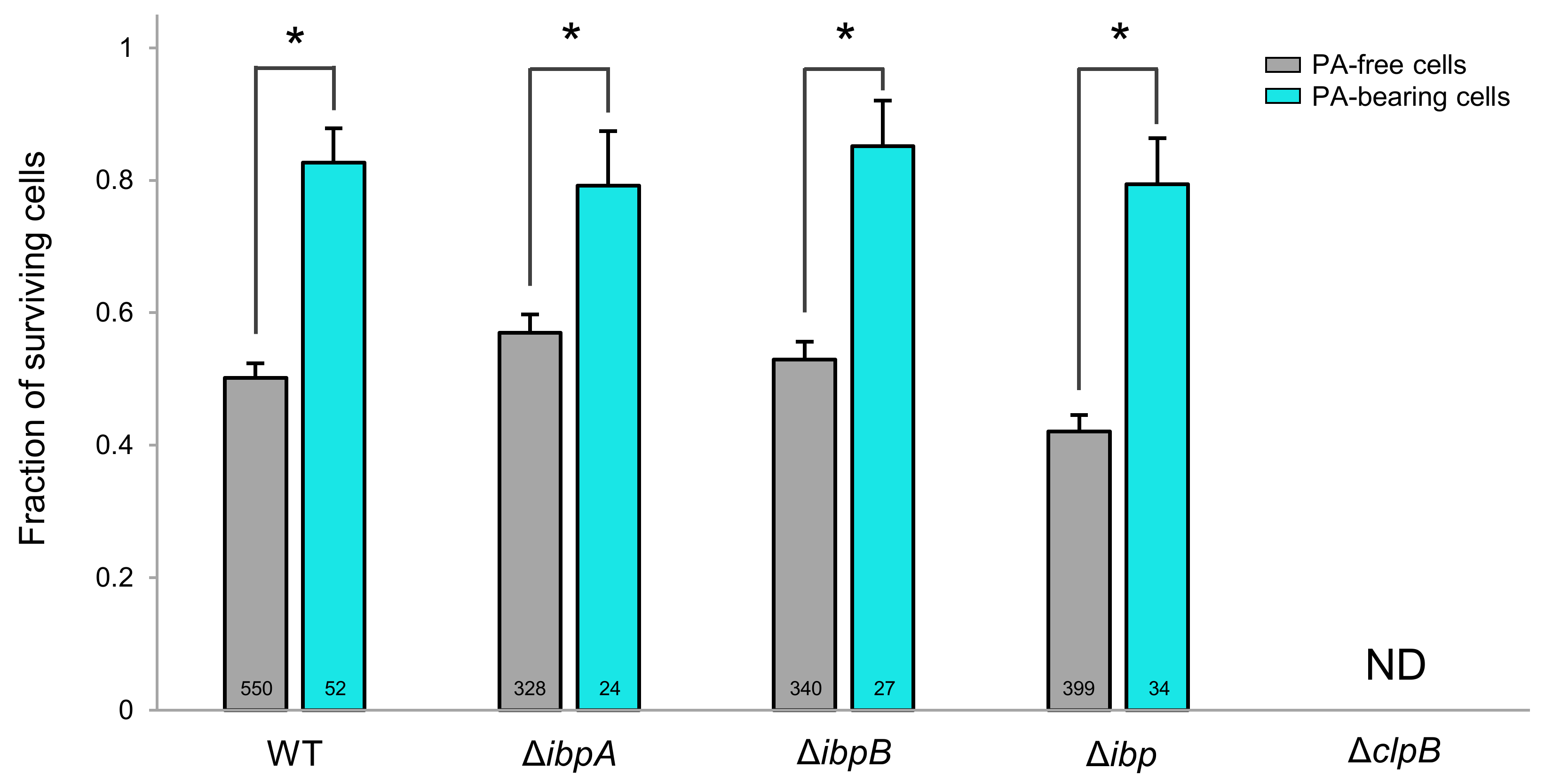

Supplement: S10 Fig — Survival probability of PA-free and PA-bearing MG1655 ΔlacY pTrc99A-mCer-cI78EP8 cells (designated as WT) and their indicated deletion mutants upon exposure to a heat shock (52 °C, 7 min). Similar experimental setup as in Fig 6E. Asterisks indicate a significant difference in survival frequency between both cellular classes (Student t test, p-values = 2.83 × 10^−7^, 1.90 × 10^−2^, 1.28 × 10^−4^, and 1.09 × 10^−5^). Numbers in black indicate the number of cells included in each bin. ND = no surviving cells could be detected within the 8 h time frame after heat shock. Error bars indicate bootstrapped estimates of the standard error of the mean fraction of surviving cells. The numerical data underlying this figure can be found in S2 Data. PA, protein aggregate; WT, wild type. (TIF) [file pbio.2003853.s010.tif]

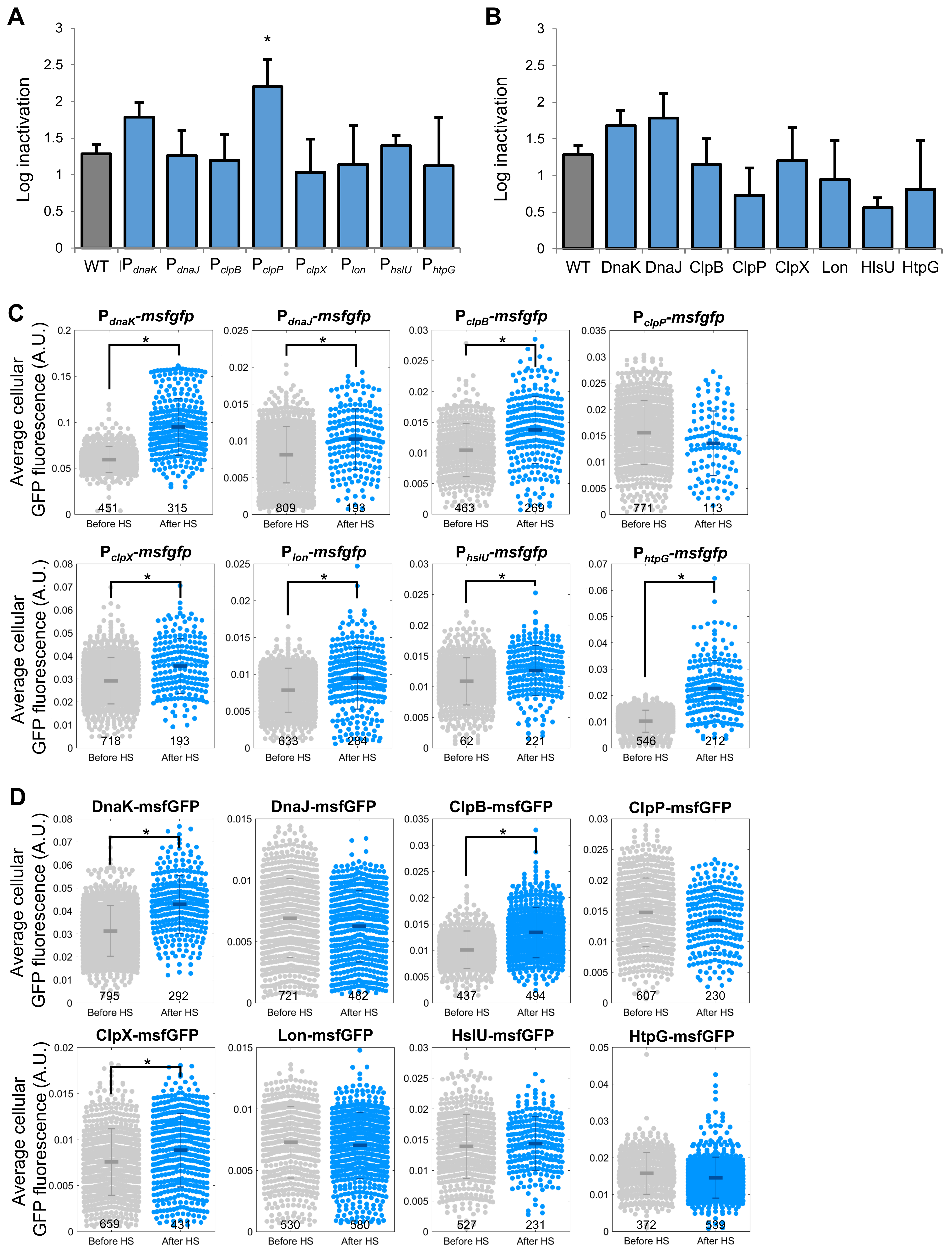

Supplement: S11 Fig — (A-B) PA-containing populations of MG1655 ΔlacY pTrc99A-mCherry-cI78EP8 (designated as WT) and the indicated (A) transcriptional and (B) translational fusion strains were exposed to a heat treatment (52 °C, 15 min), after which cellular inactivation was determined. Asterisk indicates a significant difference in inactivation of the clpP transcriptional fusion strain in comparison to its unlabeled control (Student t test, p-values = 1.84 × 10^−3^). The means of 3 independent experiments are shown, with error bars representing the standard deviation between experiments. (C) Quantification of the promoter activity of indicated protein quality control components (as measured by average cellular GFP fluorescence) in cells before and directly after a sublethal heat shock (47 °C, 15 min). Asterisks indicate a significant increase in average cellular GFP concentration in heat-shocked cells (directional Student t test, respective p-values = 1.55 × 10^−58^, 3.30 × 10^−10^, 1.56 × 10^−16^, 9.99 × 10^−1^, 2.66 × 10^−11^, 4.90 × 10^−9^, 2.96 × 10^−8^, and 3.11 × 10^−42^). Numbers in black indicate the number of cells included in each group. Error bars indicate the standard deviation. (D) Quantification of the concentration of indicated protein quality control components (as measured by average cellular GFP fluorescence) in cells before and 30 min after a sublethal heat shock (47 °C, 15 min). Asterisks indicate a significant increase in average cellular GFP concentration in heat-shocked cells (directional Student t test, respective p-values = 1.73 × 10^−37^, 9.99 × 10^−1^, 1.28 × 10^−30^, 9.99 × 10^−1^, 3.46 × 10^−8^, 9.38 × 10^−1^, 1.04 × 10^−1^, and 9.98 × 10^−1^). Numbers in black indicate the number of cells included in each group. Error bars indicate the standard deviation. The numerical data underlying this figure can be found in S2 Data. GFP, green fluorescent protein; msfGFP, monomeric superfolder GFP; PA, protein aggregate; WT, wild type. (TIF) [file pbio.2003853.s011.tif]

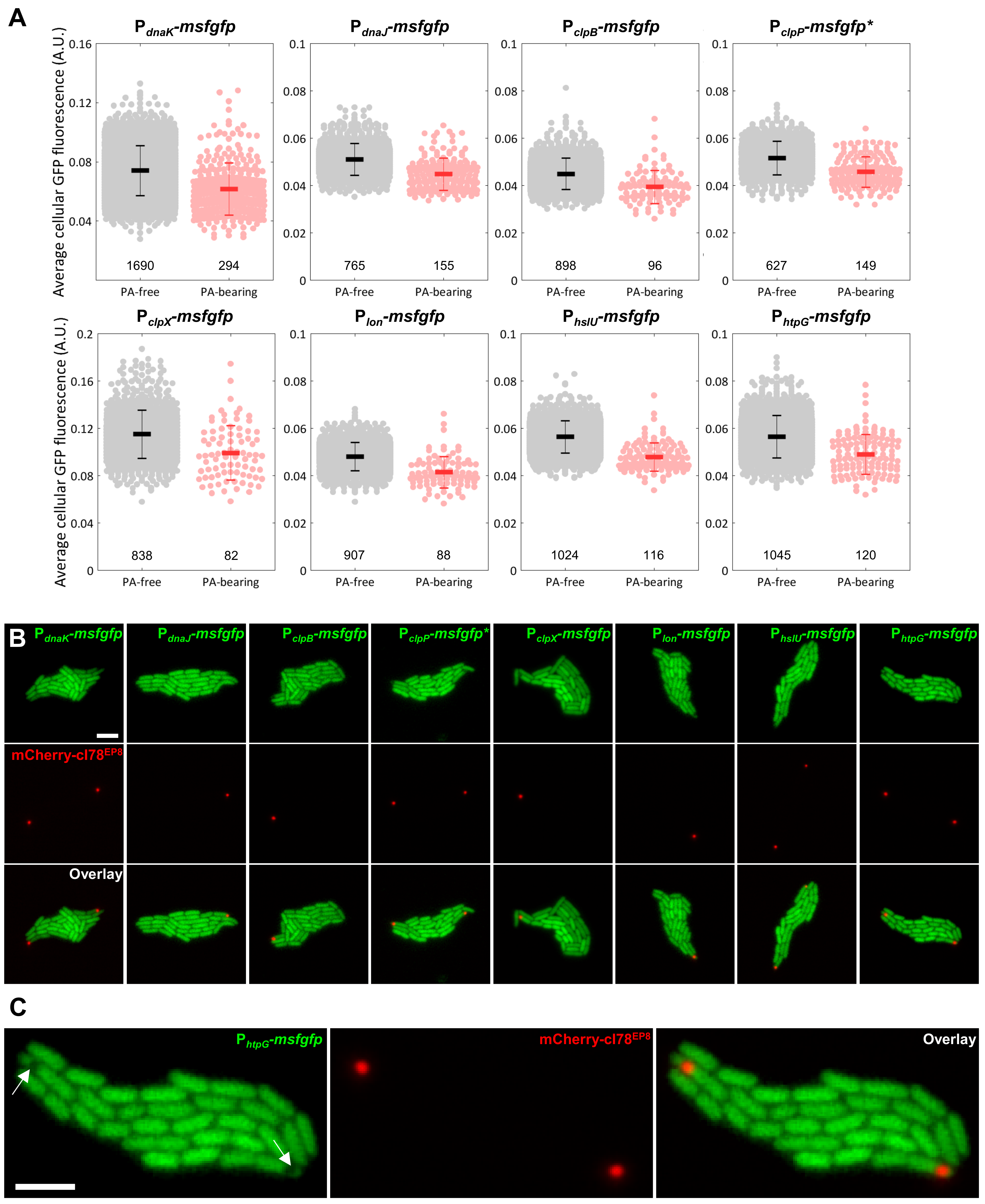

Supplement: S12 Fig — (A) Quantification of the promoter activity of indicated protein quality control components (as measured by average cellular GFP fluorescence) in PA-free and PA-bearing MG1655 ΔlacY pTrc99A-mCherry-cI78EP8 cells, 3 h after PA production was halted. No significant average up-regulation could be detected in PA-bearing cells (directional Student t test, p-value = 1 for all transcriptional fusions). Numbers in black indicate the number of cells included in each group. Error bars indicate the standard deviation. (B) Representative GFP epifluorescence (reporting expression level of the indicated gene), mCherry epifluorescence (reporting mCherry-cI78EP8 localization), and superimposed images of the indicated transcriptional fusion strains, 3 h after PA production was halted. Scale bar corresponds to 5 μm. (C) Zoomed-in images of the MG1655 ΔlacY PhtpG-msfgfp pTrc99A-mCherry-cI78EP8 strain illustrating the impermeability of PAs to cytosolic GFP (indicated by the white arrows in the GFP epifluorescence image). Scale bar corresponds to 5 μm. *Please note that the strain carrying the transcriptional clpP fusion is likely compromised, and its expression level could thus reflect nonnative behavior. The numerical data underlying this figure can be found in S2 Data. GFP, green fluorescent protein; PA, protein aggregate. (TIF) [file pbio.2003853.s012.tif]

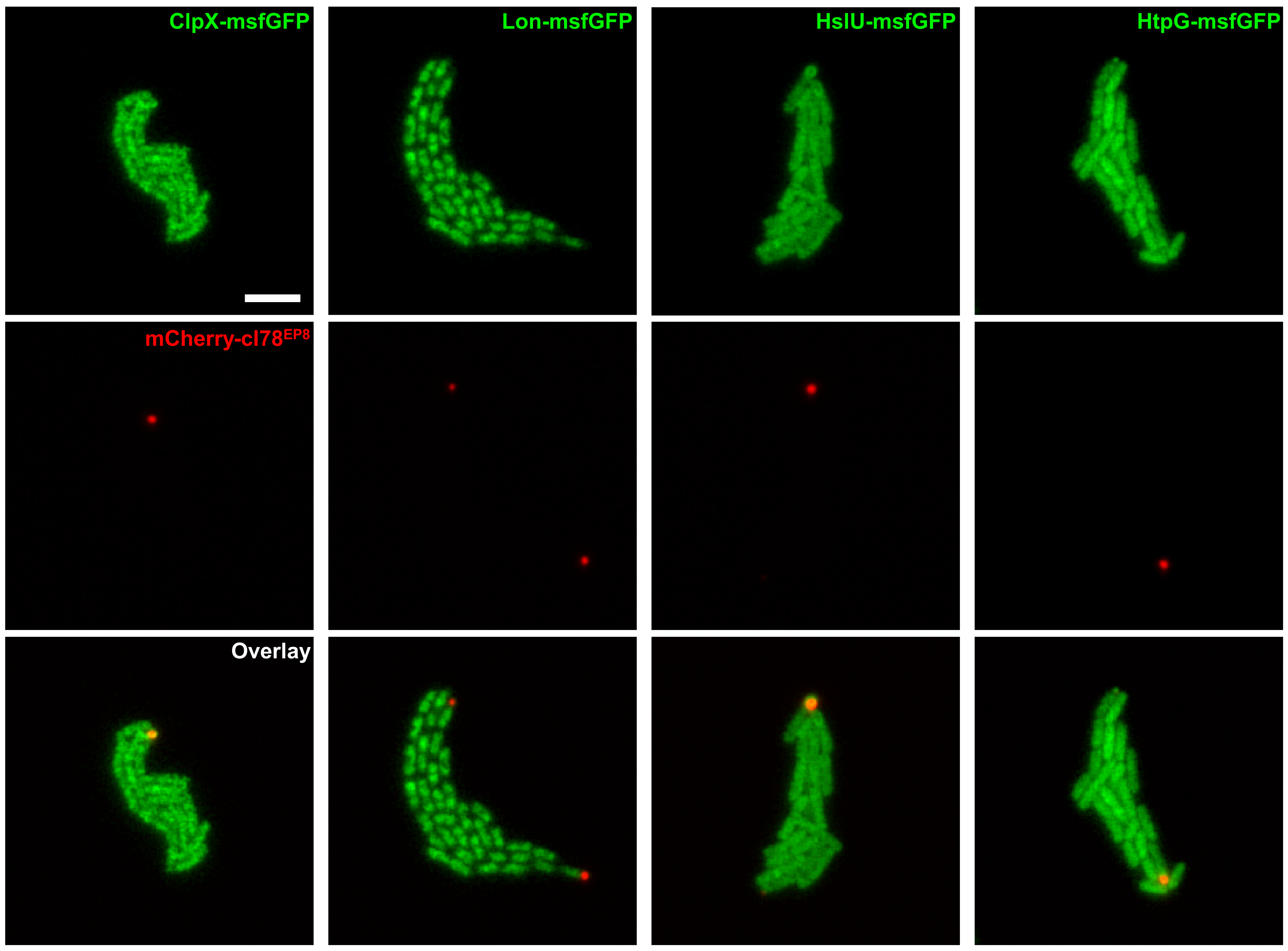

Supplement: S13 Fig — Representative GFP epifluorescence (reporting localization and concentration of the indicated translational fusion protein), mCherry epifluorescence (reporting mCherry-cI78EP8 localization), and superimposed images of the indicated translational fusion strains, 3 h after PA production was halted. Scale bar corresponds to 5 μm. The numerical data underlying this figure can be found in S2 Data. GFP, green fluorescent protein; PA, protein aggregate. (TIF) [file pbio.2003853.s013.tif]

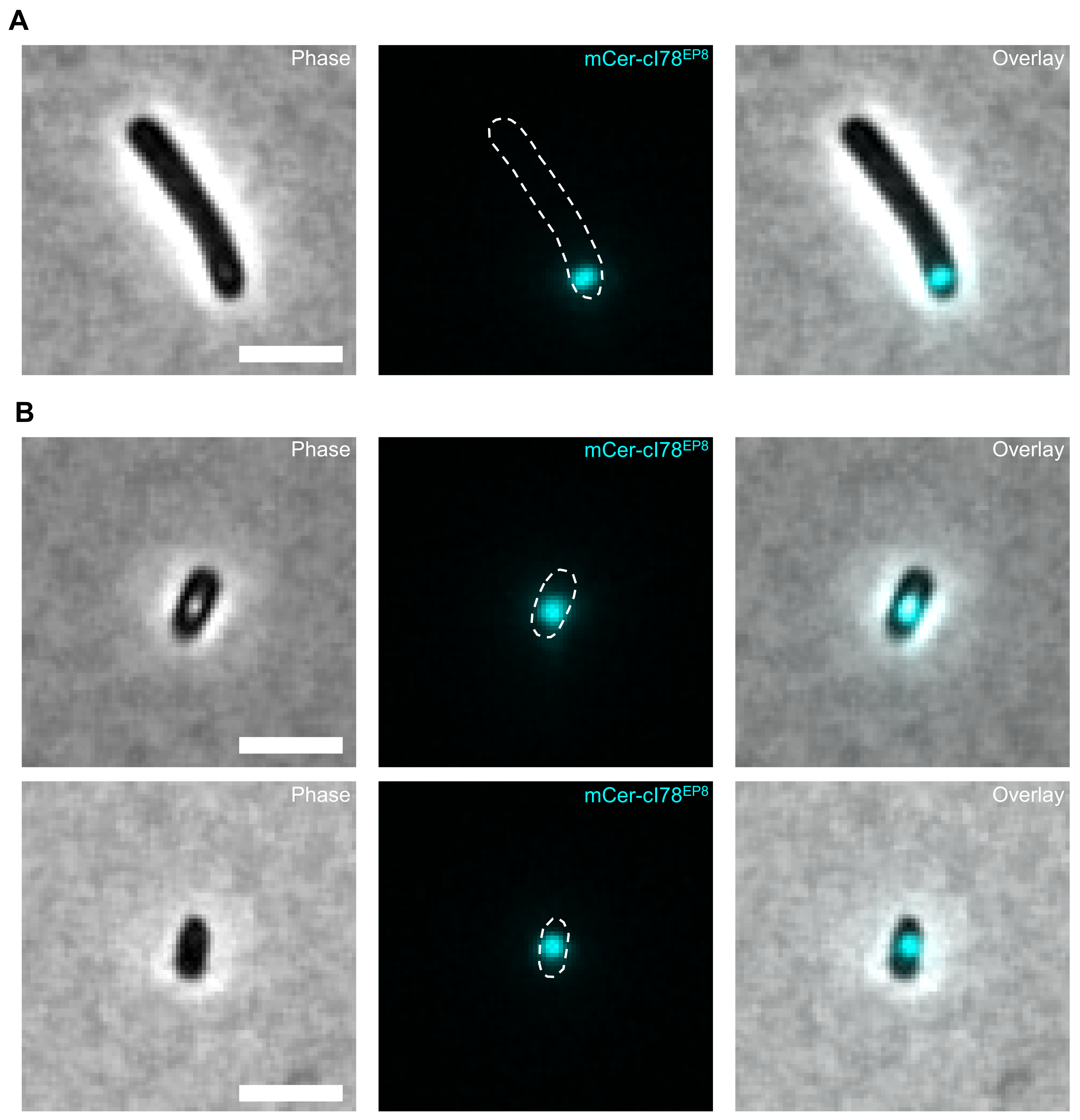

Supplement: S14 Fig — (A-B) Representative phase contrast, CFP epifluorescence (reporting mCer-cI78EP8 localization), and superimposed images of unstressed MG1655 ΔlacY pTrc99A-mCer-cI78EP8 cells after 1.5 h of induction with 1 mM IPTG. While (A) most cells are capable of growth, (B) some smaller, likely anucleate, cells are not seen to initiate or resume growth, even in control conditions in the absence of stress. All images are displayed on the same scale; scale bars correspond to 2 μm. The numerical data underlying this figure can be found in S2 Data. CFP, cyan fluorescent protein; IPTG, isopropyl β-D-1-thiogalactopyranoside; mCer, monomeric cerulean; PA, protein aggregate. (TIF) [file pbio.2003853.s014.tif]
