# Supplementary material for: Protein aggregates encode epigenetic memory of stressful encounters in individual Escherichia coli cells
Source: PLoS Biol. 2018 Aug 28;16(8):e2003853. doi: 10.1371/journal.pbio.2003853 (PMC6112618; doi:10.1371/journal.pbio.2003853)
Supplement: S1 Table — (DOCX) [file pbio.2003853.s020.docx]

**S1 Table. Amino acid sequences of fluorescent proteins.**

| **Fluorescent protein** | **Amino acid sequence** |
| --- | --- |
| Venus [[1](#_ENREF_1)] | MASKGEELFTGVVPILVELDGDVNGHKFSVSGEGEGDATYGKLTLKLICTTGKLPVPWPT  LVTTLGYGLQCFARYPDHMKRHDFFKSAMPEGYVQERTISFKDDGNYKTRAEVKFEGDTL  VNRIELKGIDFKEDGNILGHKLEYNYNSHNVYITADKQKNGIKANFKIRHNIEDGGVQLA  DHYQQNTPIGDGPVLLPDNHYLSYQSALSKDPNEKRDHMVLLEFVTAAGITHGMDELYK |
| mVenus | MASKGEELFTGVVPILVELDGDVNGHKFSVSGEGEGDATYGKLTLKLICTTGKLPVPWPT  LVTTLGYGLMCFARYPDHMKRHDFFKSAMPEGYVQERTIFFKDDGNYKTRAEVKFEGDTL  VNRIELKGIDFKEDGNILGHKLEYNYNSHNVYITADKQKNGIKANFKIRHNIEDGGVQLA  DHYQQNTPIGDGPVLLPDNHYLSYQS**K**LSKDPNEKRDHMVLLEFVTAAGITHGMDELYK |
| mCherry [[2](#_ENREF_2)] | MVSKGEEDNMAIIKEFMRFKVHMEGSVNGHEFEIEGEGEGRPYEGTQTAKLKVTKGGPLP  FAWDILSPQFMYGSKAYVKHPADIPDYLKLSFPEGFKWERVMNFEDGGVVTVTQDSSLQD  GEFIYKVKLRGTNFPSDGPVMQKKTMGWEASSERMYPEDGALKGEIKQRLKLKDGGHYDA  EVKTTYKAKKPVQLPGAYNVNIKLDITSHNEDYTIVEQYERAEGRHSTGGMDELYK |
| mCerulean3 [[3](#_ENREF_3)] | MVSKGEELFTGVVPILVELDGDVNGHKFSVSGEGEGDATYGKLTLKFICTTGKLPVPWPT  LVTTLSWGVQCFARYPDHMKQHDFFKSAMPEGYVQERTIFFKDDGNYKTRAEVKFEGDTL  VNRIELKGIDFKEDGNILGHKLEYNAIHGNVYITADKQKNGIKANFGLNCNIEDGSVQLA  DHYQQNTPIGDGPVLLPDNHYLSTQSKLSKDPNEKRDHMVLLEFVTAAGITLGMDELYK |
| msfGFP[[4](#_ENREF_4)] | MSKGEELFTGVVPILVELDGDVNGHKFSVRGEGEGDATNGKLTLKFICTTGKLPVPWPTL  VTTLTYGVQCFSRYPDHMKQHDFFKSAMPEGYVQERTISFKDDGTYKTRAEVKFEGDTLV  NRIELKGIDFKEDGNILGHKLEYNFNSHNVYITADKQKNGIKANFKIRHNVEDGSVQLAD  HYQQNTPIGDGPVLLPDNHYLSTQSKLSKDPNEKRDHMVLLEFVTAAGITHGMDELYK |

**References**

1. Nagai T, Ibata K, Park ES, Kubota M, Mikoshiba K, et al. (2002) A variant of yellow fluorescent protein with fast and efficient maturation for cell-biological applications. Nat Biotechnol 20: 87-90.

2. Shaner NC, Campbell RE, Steinbach PA, Giepmans BN, Palmer AE, et al. (2004) Improved monomeric red, orange and yellow fluorescent proteins derived from Discosoma sp. red fluorescent protein. Nat Biotechnol 22: 1567-1572.

3. Markwardt ML, Kremers GJ, Kraft CA, Ray K, Cranfill PJ, et al. (2011) An improved cerulean fluorescent protein with enhanced brightness and reduced reversible photoswitching. PLoS One 6: e17896.

4. Ke N, Landgraf D, Paulsson J, Berkmen M (2016) Visualization of Periplasmic and Cytoplasmic Proteins with a Self-Labeling Protein Tag. J Bacteriol 198: 1035-1043.
