# Supplementary material for: Protein aggregates encode epigenetic memory of stressful encounters in individual Escherichia coli cells
Source: PLoS Biol. 2018 Aug 28;16(8):e2003853. doi: 10.1371/journal.pbio.2003853 (PMC6112618; doi:10.1371/journal.pbio.2003853)
Supplement: S2 Table — (DOCX) [file pbio.2003853.s021.docx]

**S2 Table**. **Overview of strain used in this study.**

| **Strain** | **Description** | **Source or reference** |
| --- | --- | --- |
| MG1655 | *E. coli* MG1655 wild-type | Blattner et al., 1997 [[10](#_ENREF_10)] |
| MG1655 *ibpA-yfp* | MG1655 encoding a C-terminal fusion of IbpA to YFP | Lindner et al., 2008 [[11](#_ENREF_11)] |
| MG1655 *ibpA-venus* | MG1655 encoding a C-terminal fusion of IbpA to Venus | This study |
| MG1655 *ibpA-mVenus* | MG1655 encoding a C-terminal fusion of IbpA to mVenus | This study |
| MG1655 *ibpA-mCherry* | MG1655 encoding a C-terminal fusion of IbpA to mCherry | This study |
| MG1655 *ibpA-mCer* | MG1655 encoding a C-terminal fusion of IbpA to mCerulean3 | This study |
| MG1655 *ibpA-msfgfp* | MG1655 encoding a C-terminal fusion of IbpA to msfGFP | This study |
| MG1655 *ibpB-venus* | MG1655 encoding a C-terminal fusion of IbpB to YFP | This study |
| MG1655 *ibpB-msfgfp* | MG1655 encoding a C-terminal fusion of IbpB to msfGFP | This study |
| MG1655 Δ*lacY* | MG1655 with a deletion of *lacY* | This study |
| MG1655 Δ*lacY* Δ*ibpA* | MG1655 Δ*lacY* with a deletion of *ibpA* | This study |
| MG1655 Δ*lacY* Δ*ibpB* | MG1655 Δ*lacY* with a deletion of *ibpB* | This study |
| MG1655 Δ*lacY* Δ*ibp* | MG1655 Δ*lacY* with a deletion of the entire *ibp* operon | This study |
| MG1655 Δ*lacY* Δ*clpB* | MG1655 Δ*lacY* with a deletion of *clpB* | This study |
| MG1655 Δ*lacY* Δ*recA* | MG1655 ibpA-yfp with a deletion of *recA* | This study |
| MG1655 *hupA-venus* | MG1655 encoding a C-terminal fusion of HupA to mCherry | This study |
| MG1655 *dnaK-msfgfp* | MG1655 encoding a C-terminal fusion of DnaK to msfGFP | This study |
| MG1655 *dnaJ-msfgfp* | MG1655 encoding a C-terminal fusion of DnaJ to msfGFP | This study |
| MG1655 *clpB-msfgfp* | MG1655 encoding a C-terminal fusion of ClpB to msfGFP | This study |
| MG1655 *clpP-msfgfp* | MG1655 encoding a C-terminal fusion of ClpP to msfGFP | This study |
| MG1655 *clpX-msfgfp* | MG1655 encoding a C-terminal fusion of ClpX to msfGFP | This study |
| MG1655 *htpG-msfgfp* | MG1655 encoding a C-terminal fusion of HtpG to msfGFP | This study |
| MG1655 *hslU-msfgfp* | MG1655 encoding a C-terminal fusion of HslU to msfGFP | This study |
| MG1655 *lon-msfgfp* | MG1655 encoding a C-terminal fusion of Lon to msfGFP | This study |
| MG1655 P*_dnaK_-msfgfp* | MG1655 encoding a transcriptional fusion of *dnaK* to msfGFP | This study |
| MG1655 P*_dnaJ_-msfgfp* | MG1655 encoding a transcriptional fusion of *dnaJ* to msfGFP | This study |
| MG1655 P*_clpB_-msfgfp* | MG1655 encoding a transcriptional fusion of *clpB* to msfGFP | This study |
| MG1655 P*_clpP_-msfgfp* | MG1655 encoding a transcriptional fusion of *clpP* to msfGFP | This study |
| MG1655 P*_clpX_-msfgfp* | MG1655 encoding a transcriptional fusion of *clpX* to msfGFP | This study |
| MG1655 P*_htpG_-msfgfp* | MG1655 encoding a transcriptional fusion of *htpG* to msfGFP | This study |
| MG1655 P*_hslU_-msfgfp* | MG1655 encoding a transcriptional fusion of *hslU* to msfGFP | This study |
| MG1655 P*_lon_-msfgfp* | MG1655 encoding a transcriptional fusion of *lon* to msfGFP | This study |
