# Supplementary material for: Protein aggregates encode epigenetic memory of stressful encounters in individual Escherichia coli cells
Source: PLoS Biol. 2018 Aug 28;16(8):e2003853. doi: 10.1371/journal.pbio.2003853 (PMC6112618; doi:10.1371/journal.pbio.2003853)
Supplement: S3 Table — (DOCX) [file pbio.2003853.s022.docx]

S3 Table. Overview of plasmids used in this study.

| **Plasmid** | **Description** | **Source or reference** |
| --- | --- | --- |
| pSG1 | pACYC184 vector backbone [[1](#_ENREF_1)] encoding mCherry under control of the *ibpA* promoter | This study |
| pTVP1LAC | Encoding an engineered *E. coli* β-galactosidase fused to the aggregation-prone foot-and-mouth disease virus (FMDV) VP1 capsid protein | Garcia-Fruitos et al., 2005 [[2](#_ENREF_2)] |
| pTVP1RFP | Encoding a red fluorescent protein (RFP) fused to the aggregation-prone foot-and-mouth disease virus (FMDV) VP1 capsid protein | Garcia-Fruitos et al., 2005 [[2](#_ENREF_2)] |
| pMAL LRRK2 | Encoding an N-terminal fusion of the human Leucine-Rich Repeat Kinase 2 (LRRK2) to *E. coli* MalE (maltose-binding protein) | Taymans, J.-M., KU Leuven |
| pCP20 | Encodes Flp for recombining *frt* sites (excising sequences in between) | Cherepanov and Wackernagel, 1995 [[3](#_ENREF_3)] |
| pKD46 | Encodes lambda *red* genes under control of an arabinose-inducible promoter | Datsenko and Wanner, 2000 [[4](#_ENREF_4)] |
| pKD13 | Harbors *frt-nptI-frt* cassette for construction of deletions by recombineering | Datsenko and Wanner, 2000 [[4](#_ENREF_4)] |
| pDHL1029 | Harbors *msfgfp-frt-nptI-frt* for construction of msfGFP fusions | Ke et al., 2016 [[5](#_ENREF_5)] |
| pDHL-*venus* | Harbors *venus-frt-nptI-frt* for construction of Venus fusions | This study |
| pDHL-mV*enus* | Harbors *mVenus-frt-nptI-frt* for construction of mVenus fusions | This study |
| pDHL-*mCherry* | Harbors *mCherry-frt-nptI-frt* for construction of mCherry fusions | This study |
| pDHL-*mCer* | Harbors *mCerulean3-frt-nptI-frt* for construction of mCerulean3 fusions | This study |
| pGBKD-*mCherry* | Harbors *mCherry-frt-cat-frt* for construction of mCherry fusions | This study |
| pGBKD-*venus* | Harbors *venus-frt-cat-frt* for construction of Venus fusions | This study |
| pTrc99A-*mCer-cI78^WT^* | pTrc99A expression vector allowing the inducible expression (P*_trc_* promoter) of an N-terminal fusion of cI78^WT^ (a fragment of the lambda prophage repressor protein) to the mCerulean3 fluorescent protein | This study |
| pTrc99A-*mCer-cI78^EP8^* | pTrc99A expression vector allowing the inducible expression (P*_trc_* promoter) of a N-terminal fusion of cI78^EP8^ (a mutagenized fragment of the lambda prophage repressor protein) to the mCerulean3 fluorescent protein | This study |
| pTrc99A-*mCherry-cI78^EP8^* | pTrc99A expression vector allowing the inducible expression (P*_trc_* promoter) of a N-terminal fusion of cI78^EP8^ (a mutagenized fragment of the lambda prophage repressor protein) to the mCherry fluorescent protein | This study |
| pBAD33-*ibpA-msfgfp* | pBAD33 expression vector allowing the inducible expression (P*_BAD_* promoter) of the IbpA-msfGFP fusion protein. | This study |

**References**

1. Chang AC, Cohen SN (1978) Construction and characterization of amplifiable multicopy DNA cloning vehicles derived from the P15A cryptic miniplasmid. J Bacteriol 134: 1141-1156.

2. Garcia-Fruitos E, Gonzalez-Montalban N, Morell M, Vera A, Ferraz RM, et al. (2005) Aggregation as bacterial inclusion bodies does not imply inactivation of enzymes and fluorescent proteins. Microb Cell Fact 4: 27.

3. Cherepanov PP, Wackernagel W (1995) Gene disruption in Escherichia coli: TcR and KmR cassettes with the option of Flp-catalyzed excision of the antibiotic-resistance determinant. Gene 158: 9-14.

4. Datsenko KA, Wanner BL (2000) One-step inactivation of chromosomal genes in Escherichia coli K-12 using PCR products. Proc Natl Acad Sci U S A 97: 6640-6645.

5. Ke N, Landgraf D, Paulsson J, Berkmen M (2016) Visualization of Periplasmic and Cytoplasmic Proteins with a Self-Labeling Protein Tag. J Bacteriol 198: 1035-1043.
