# Supplementary material for: Protein aggregates encode epigenetic memory of stressful encounters in individual Escherichia coli cells
Source: PLoS Biol. 2018 Aug 28;16(8):e2003853. doi: 10.1371/journal.pbio.2003853 (PMC6112618; doi:10.1371/journal.pbio.2003853)
Supplement: S4 Table — When relevant, primer attachment sites are indicated in bold, linker sequences in blue, spacer sequences in orange, artificial ribosome binding sites in purple, and restriction sites in red. (DOCX) [file pbio.2003853.s023.docx]

S4 Table. Overview of primers used in this study. When relevant, primer attachment sites are indicated in bold, linker sequences in blue, spacer sequences in orange, artificial ribosome binding sites in purple and restriction sites in red.

| **Primer name** | **Sequence** |
| --- | --- |
| SG1 | TCGAACGCGTGATTCCGGAAGCGAAAAAACCGCGCCGTATCGAAATCAAC**AGCGGTGGCGGTGGC** |
| SG2 | CCTGACGGCGAGCATGGAGATGTCAGGCCGCGCCAGGCGGCCTTAGGGAATTAGTTGATT**ATTCCGGGGATCCGTCGACC** |
| SG3 | CCATCGCAGCGCAGCGTATCGCTATCAGCGAACGTCCCGCGTTAAATAGC**AGCGGTGGCGGTGGC** |
| SG4 | GTAAGACAAAAAAAGCCCCACCAGAATGGCGGGGCAAAGAGAATAGCTAG**ATTCCGGGGATCCGTCGACC** |
| SG5 | CTAACGTACCGGCATTTGTTTCTGGCAAGGCACTGAAAGACGCAGTTAAG**GGCAGCGGCAGCGGCA** |
| SG6 | AAAAGGGGTGAAACCACCCCTTCGTTAAAACTGTTCACTGCCACGCAATC**GTGTAGGCTGGAGCTGCTTC** |
| SG7 | AGAATTCGGCAGCGGCAGCGGCAGC**GTGAGCAAGGGCGAGGA** |
| SG8 | AGGATCCTTACTTGTACAGCTCGTCCA |
| SG9 | AGAATTCGGCAGCGGCAGCGGCAGC**GCTAGCAAAGGAGAAGAACT** |
| SG10 | AGGATCCTTATTTGTAGAGCTCATCCATG |
| SG11 | GTCCATTGTGGAAGGTCTTACATTCTCGCTGATTTCAGGAGCTATTGATTATG**GTGAGCAAGGGCGAGGAG** |
| SG12 | CCTGACGGCGAGCATGGAGATGTCAGGCCGCGCCAGGCGGCCTTAGGGAATTAGTTGATT**GTGTAGGCTGGAGCTGCTTC** |
| SG13 | **GCAAAGTGCGTCGGGTGAT** |
| SG14 | **GCCGAATAAATACCTGTGACG** |
| SG15 | TGCCATGGCA**GTGAGCAAGGGCGAGGAG** |
| SG16 | AGGATCC**CTTGTACAGCTCGTCCATGC** |
| SG17 | AGGATCCGGCAGCGGCAGC**AGCCCTTCAATCGCCAGAGA** |
| SG18 | TGAGTCGAC**TCAGCCAAACGTCTCTTCAGGC** |
| SG19 | AAAGAGGAGAATACTAG**ATGCGTAACTTTGATTTATCCCCGCT** |
| SG20 | TTATTTGTAGAGTTCATCCATGCCGT |
| SG21 | CTCGGTACCCGGGGATCCTC |
| SG22 | CTCGAATTCGCTAGCCCAAA |
| SG23 | AAGATGACGATGTTGTCGACGCTGAATTTGAAGAAGTCAAAGACAAAAAA**AGCGGTGGCGGTGGC** |
| SG24 | AGGAAATTCCCCTTCGCCCGTGTCAGTATAATTACCCGTTTATAGGGCGA**ATTCCGGGGATCCGTCGACC** |
| SG25 | CAAAGAGCTTCTTTGATGGTGTGAAGAAGTTTTTTGACGACCTGACCCGC**AGCGGTGGCGGTGGC** |
| SG26 | ACGCACCCTATTTTTACCCAGGCCTGCCCACGGGCAGGCTTTTGGGGAGG**ATTCCGGGGATCCGTCGACC** |
| SG27 | GTAAAGTGATTCGCCTGGAAGTTAATGAAGACCGGATTGTCGCCGTCCAG**AGCGGTGGCGGTGGC** |
| SG28 | TCCGTCTAACTTATAGACAAAAACGAGCCCCGAAGGGCTCGTTTTATCAT**ATTCCGGGGATCCGTCGACC** |
| SG29 | CTGAAGCGGTGGAATACGGTCTGGTCGATTCGATTCTGACCCATCGTAAT**AGCGGTGGCGGTGGC** |
| SG30 | AGCGTTGTGCCGCCCTGGATAAGTATAGCGGCACAGTTGCGCCTCTGGCA**ATTCCGGGGATCCGTCGACC** |
| SG31 | AACCGTTGCTGATTTATGGCAAGCCGGAAGCGCAACAGGCATCTGGTGAA**AGCGGTGGCGGTGGC** |
| SG32 | GGAGATAAAATCCCCCCTTTTTGGTTAACTAATTGTATGGGAATGGTTAA**ATTCCGGGGATCCGTCGACC** |
| SG33 | TGGAAGATCCGAACCTGTTTATTCGTCGTATGAACCAGCTGCTGGTTTCC**AGCGGTGGCGGTGGC** |
| SG34 | GATGATGAAAAGAAAAATGCCGGATGACACGAAGGTCATCCGGCATTACA**ATTCCGGGGATCCGTCGACC** |
| SG35 | AACATCTGGATGCGTTGGTGGCAGATGAAGATCTGAGCCGTTTTATCCTA**AGCGGTGGCGGTGGC** |
| SG36 | AATGGGGCCTTTCAGCCCCATCAAACAATGATGAAAATGATTGAACGCGA**ATTCCGGGGATCCGTCGACC** |
| SG37 | CTCTGGCGCTGCAAAATGAACCGTCTGGTATGCAGGTTGTGACTGCAAAA**AGCGGTGGCGGTGGC** |
| SG38 | CGAATTAGCCTGCCAGCCCTGTTTTTATTAGTGCATTTTGCGCGAGGTCA**ATTCCGGGGATCCGTCGACC** |
| SG39 | GATGTTGTCGACGCTGAATTTGAAGAAGTCAAAGACAAAAAATAATCGCCAAAGAGGAGAATACTAGATG**AGTAAAGGTGAAGAACTGTTCACCGG** |
| SG40 | CGGAGAGGAAATTCCCCTTCGCCCGTGTCAGTATAATTACCCGTTTATAG**ATTCCGGGGATCCGTCGACC** |
| SG41 | TTCTTTGATGGTGTGAAGAAGTTTTTTGACGACCTGACCCGCTAACCTCCAAAGAGGAGAATACTAGATG**AGTAAAGGTGAAGAACTGTTCACCGG** |
| SG42 | CTTCAACGCACCCTATTTTTACCCAGGCCTGCCCACGGGCAGGCTTTTGG**ATTCCGGGGATCCGTCGACC** |
| SG43 | ATTCGCCTGGAAGTTAATGAAGACCGGATTGTCGCCGTCCAGTAAATGATAAAGAGGAGAATACTAGATG**AGTAAAGGTGAAGAACTGTTCACCGG** |
| SG44 | TCTTTTCCGTCTAACTTATAGACAAAAACGAGCCCCGAAGGGCTCGTTTT**ATTCCGGGGATCCGTCGACC** |
| SG45 | GTGGAATACGGTCTGGTCGATTCGATTCTGACCCATCGTAATTGATGCCAAAAGAGGAGAATACTAGATG**AGTAAAGGTGAAGAACTGTTCACCGG** |
| SG46 | CTTACAGCGTTGTGCCGCCCTGGATAAGTATAGCGGCACAGTTGCGCCTC**ATTCCGGGGATCCGTCGACC** |
| SG47 | CTGATTTATGGCAAGCCGGAAGCGCAACAGGCATCTGGTGAATAATTAACAAAGAGGAGAATACTAGATG**AGTAAAGGTGAAGAACTGTTCACCGG** |
| SG48 | AAAGGGGAGATAAAATCCCCCCTTTTTGGTTAACTAATTGTATGGGAATG**ATTCCGGGGATCCGTCGACC** |
| SG49 | CCGAACCTGTTTATTCGTCGTATGAACCAGCTGCTGGTTTCCTGATGTAAAAAGAGGAGAATACTAGATG**AGTAAAGGTGAAGAACTGTTCACCGG** |
| SG50 | GTGCAGATGATGAAAAGAAAAATGCCGGATGACACGAAGGTCATCCGGCA**ATTCCGGGGATCCGTCGACC** |
| SG51 | GATGCGTTGGTGGCAGATGAAGATCTGAGCCGTTTTATCCTATAATCGCGAAAGAGGAGAATACTAGATG**AGTAAAGGTGAAGAACTGTTCACCGG** |
| SG52 | ATAAAAATGGGGCCTTTCAGCCCCATCAAACAATGATGAAAATGATTGAA**ATTCCGGGGATCCGTCGACC** |
| SG53 | CTGCAAAATGAACCGTCTGGTATGCAGGTTGTGACTGCAAAATAGTGACCAAAGAGGAGAATACTAGATG**AGTAAAGGTGAAGAACTGTTCACCGG** |
| SG54 | AAGCCCGAATTAGCCTGCCAGCCCTGTTTTTATTAGTGCATTTTGCGCGA**ATTCCGGGGATCCGTCGACC** |
| SG55 | **AATAAATTCATCTGTTGATCGTGGGT** |
| SG56 | **TTACTTGTACAGCTCGTCCATGCC** |
